# Design, automated synthesis and immunological evaluation of NOD2-ligand–antigen conjugates

**DOI:** 10.3762/bjoc.10.148

**Published:** 2014-06-26

**Authors:** Marian M J H P Willems, Gijs G Zom, Nico Meeuwenoord, Ferry A Ossendorp, Herman S Overkleeft, Gijsbert A van der Marel, Jeroen D C Codée, Dmitri V Filippov

**Affiliations:** 1 Leiden Institute of Chemistry, Leiden University, P.O. Box 9502, 2300 RA Leiden, The Netherlands; 2 Department of Immunohematology and Blood Transfusion, Leiden University Medical Centre, P. O. Box 9600, 2300 RC Leiden, The Netherlands

**Keywords:** automated synthesis, glycopeptide, innate immunity, muramyl dipeptide, NOD2 receptor, solid phase synthesis

## Abstract

The covalent attachment of an innate immune system stimulating agent to an antigen can provide active vaccine modalities capable of eliciting a potent immune response against the incorporated antigen. Here we describe the design, automated synthesis and immunological evaluation of a set of four muramyl dipeptide–peptide antigen conjugates. Muramyl dipeptide (MDP) represents a well-known ligand for the intracellular NOD2 receptor and our study shows that covalently linking an MDP-moiety to an antigenic peptide can lead to a construct that is capable of stimulating the NOD2 receptor if the ligand is attached at the anomeric center of the muramic acid. The constructs can be processed by dendritic cells (DCs) and the conjugation does not adversely affect the presentation of the incorporated SIINFEKL epitope on MHC-I molecules. However, stimulation of the NOD2 receptor in DCs was not sufficient to provide a strong immunostimulatory signal.

## Introduction

In recent years the study of pattern recognition receptors (PRRs) and associated ligands has evolved tremendously [1]. The discovery of Toll-like receptors (TLRs) [2] in the late 1990s has had a major impact on the field of immunology. This is reflected in the exploration of conjugates consisting of a PRR-ligand (PRR-L) covalently bound to antigenic proteins and oligopeptides in the development of new (semi)synthetic vaccine modalities [3–9]. For instance, the group of Boons investigated a three-component conjugate containing a tumor-associated glycopeptide and a T cell epitope covalently bound to a TLR2 ligand [10–11]. Previously we have reported on the design, synthesis and immunological evaluation of constructs of TLR ligands covalently linked to a synthetic long peptide harboring a major histocompatibility complex (MHC) class I specific epitope [12]. Either the structurally defined TLR2-ligand Pam_3_CSK_4_ [13–14], TLR7-ligand 7-hydroxy-8-oxoadenine [15] or TLR9-ligand CpG DNA [13] were covalently bound to a model antigen, an ovalbumin derived peptide comprising the MHC I epitope SIINFEKL, embedded in a longer peptide motif (DEVSGLEQLESIINFEKLAAAAAK, DEVA_5_K) [13,16]. We revealed that conjugates, in which Pam_3_CSK_4_ or CpG DNA were incorporated, showed an increased uptake of conjugated peptide. Increased DC maturation and enhanced antigen presentation was achieved in comparison to the mixture of the single peptide and ligands [13–14].

**Figure 1:**
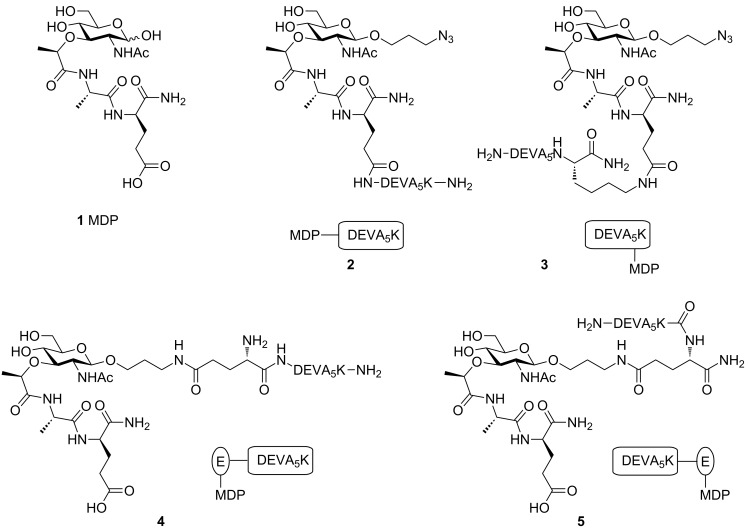
MDP-antigen conjugates **2**–**5**. DEVA_5_K = DEVSGLEQLESIINFEKLAAAAAK.

In the late 1990s the cytosolic nucleotide-binding oligomerization domain (NOD) receptors [17] NOD1 [18] and NOD2 [19] were discovered to be intracellular PRRs [20–21]. NOD1 and NOD2 recognize specific parts of peptidoglycan (PG), found in the bacterial cell wall [22–23]. PG consists of a polysaccharide chain of β-(1–4) linked *N*-acetylglucosamine (GlcNAc) and *N*-acetylmuramic acid (MurNAc) of which the lactic acid is connected to a peptide [24]. Where NOD1 recognizes diaminopimelic acid containing peptides [25–26], the minimal structural element of PG required for activation of the intracellular protein NOD2 is *N*-acetylmuramyl-L-alanine-D-isoglutamine (MDP, Figure 1, **1**) [27–30]. Based on the effectiveness of the above mentioned TLR2/9-ligand conjugates, we hypothesized that constructs consisting of a NOD2 ligand covalently bound to the ovalbumin derived model peptide (DEVA_5_K) could lead to similar enhanced immunological activity. Important issues in the design of such conjugates are the selection of a suitable MDP derivative and the position of the covalent linkage between the NOD2-ligand and the peptide epitope [31–33]. Besides, the nature of the interaction between MDP and NOD2 receptor at the molecular level has not been established yet. Therefore we considered different linkages between MDP and the antigenic peptide (Figure 1) and we decided to hook up the NOD2 ligand to either the N- or C-terminus. As a point of attachment on the ligand we opted for a linker connected to the anomeric center of the MDP or the carboxylic acid function of isoglutamine as viable conjugation sites. It is known that the size and orientation of the aglycon installed at the anomeric center of MDP influences its immunological activity [18,34] and therefore we selected an 3-azidopropanol spacer as conjugation handle with minimal steric bulk. With respect to the second conjugation site, i.e., the isoglutamine residue, it has been reported that condensation of this acid with an unnatural amine does not affect the immunological properties of MDP [34]. A conjugate of the anticancer drug Paclitaxel with MDP has been described using this conjugation locus, which not only showed antitumor activity but also immunostimulatory effects [35]. Also, the commercially available immunomodulator Murabutide, a glutamine *n*-butyl ester derivative of MDP, supports the notion that modifications on the isoglutamine are allowed [36]. Thus, MDP-antigen conjugates **2**–**5** were selected as target molecules (Figure 1). In conjugate **2** the carboxylic acid function of the isoglutamine of the MDP is linked to the N-terminal amine of the antigenic peptide. In conjugate **3** the same acid function of the MDP connects to the C-terminal lysine of the antigenic peptide. The 3-azidopropanol spacers at the anomeric center of MurNAc in conjugate **2** and **3** remain unmodified. In conjugates **4** and **5** on the other hand the 3-azidopropanol is functionalized with glutamic acid allowing conjugation to the antigenic peptide at the N-terminus for conjugate **4** and at the C-terminus for conjugate **5**.

We here describe the assembly of MDP building blocks, suitable for automated solid phase peptide synthesis (SPPS), their use in the assembly of the four MDP-antigen conjugates **2**–**5** and the immunological evaluation of the constructs.

## Results and Discussion

### Synthesis of the conjugates

**Scheme 1:**
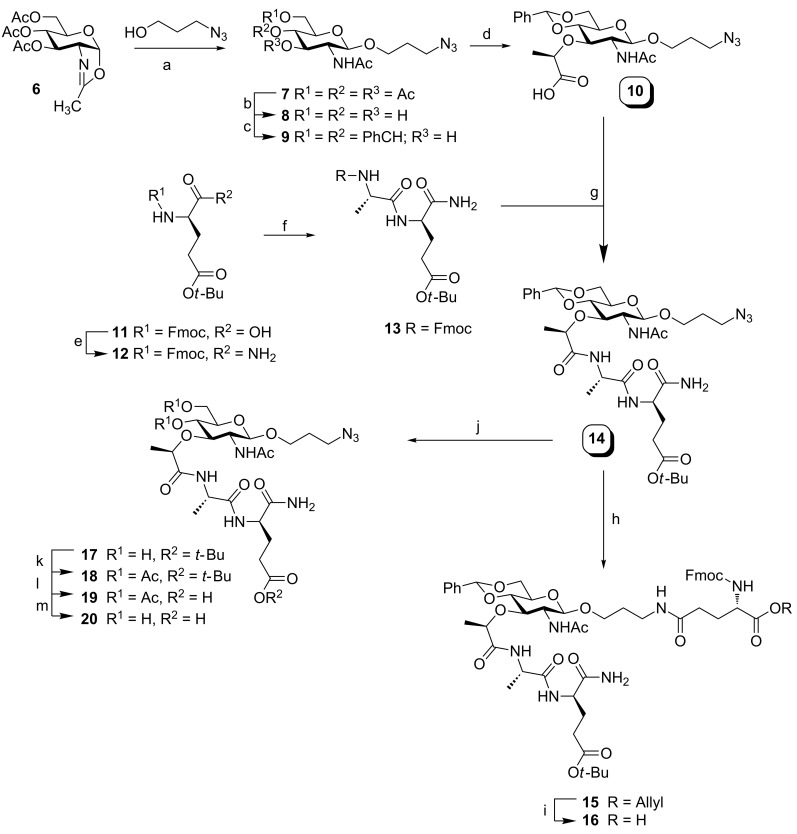
a) TMSOTf, DCM, 83%; b) cat. NaOMe, MeOH, quant.; c) CSA, PhCH(OMe)_2_, MeCN, DMF, 86%; d) (*S*)-2-chloropropionic acid, NaH, 1,4-dioxane, 93%; e) Boc_2_O, NH_4_HCO_3_, pyridine, 1,4-dioxane, 99%; f) 1) DBU, HOBt, DCM; 2) Fmoc-L-Ala-OH, EDC, DIPEA, DCM, 82%; g) 1) **13**, DBU, HOBt, DCM; 2) **12**, HATU, DIPEA, DCM, 70%; h) 1) Me_3_P (solution in THF), DMF, THF, 80%; 2) Fmoc-Glu-(OH)-OAllyl, HATU, DIPEA, DMF, 57%; i) Pd(PPh_3_)_4_, Bu_3_SnH, AcOH, DMF, 72%; j) 60% AcOH, H_2_O, neopentylglycol, 88%; k) Ac_2_O, pyridine; l) 20% TFA, DCM, 82% (2 steps); m) NH_4_OH, MeOH, 87%.

The MDP-antigen conjugates **2**–**5** were prepared using an automated solid-phase peptide synthesis (SPPS) protocol. In all these syntheses commercially available Tentagel S RAM resin and amino acids were applied. The synthesis of the required MDP building blocks **10** and **16** is depicted in Scheme 1. The 3-azidopropanol spacer was attached to the anomeric center of the glucosamine moiety using oxazoline **6** [37–39]. Deacetylation and subsequent installation of the benzylidene protective group then gave alcohol **9**. Coupling of **9** with (*S*)-2-chloropropanoic acid in the presence of sodium hydride resulted in the formation of protected MurNAc **10** in 93% yield [39–40]. Fully protected MDP **14** was obtained by condensation of acid **10** and dipeptide **13**. To this end, Fmoc-protected *tert*-butyl glutamic acid **11** was reacted with di-*tert-*butyl dicarbonate and transformed into **12** [41]. In a one-pot procedure compound **12** was deprotected with DBU and after quenching the reaction mixture with HOBt, the free amine was condensed with Fmoc-protected alanine to give compound **13** in 82% yield. The same procedure was applied to condense MurNAc **10** with dipeptide **13**, using the more reactive coupling reagent HATU. Despite the low solubility of MurNAc **10** and dipeptide **13**, building block **14** was obtained in 70% yield. Isolation and purification of key intermediate **14** was substantially facilitated by the finding that the compound could be efficiently precipitated from a MeOH/DCM/diethyl ether solvent mixture. The synthesis of building block **16** started with the reduction of the azide in compound **14** with PMe_3_ and subsequently the primary amine and Fmoc protected glutamic acid allyl ester were condensed under influence of HATU and DiPEA to give the orthogonally protected compound **15** in 57% yield. To make **15** suitable for SPPS, the allyl protective group was removed with Bu_3_SnH and Pd(PPh_3_)_4_ under acidic conditions yielding compound **16** in 72% [42].

For the immunological evaluation of conjugates **2**–**5** relevant reference compounds are needed, and to this end we assembled MDP derivative **20**. On paper, acidic removal of the Boc and benzylidene protecting groups in **14** could lead to reference compound **20** in a single step. However, treatment of **14** with a solution of 20% TFA in DCM was accompanied by hydrolysis of the glycosidic linkage. To suppress acid-mediated hydrolysis a stepwise deprotection procedure was followed in which the benzylidene group was first replaced by electron-withdrawing acetyl groups. This protective group pattern provides higher acid stability of the glycosidic linkage and permits removal of the Boc group using more stringent conditions. Thus, treatment of **14** with 60% aqueous acetic acid in the presence of two equivalents of neopentyl glycol at 60 °C and careful monitoring of the reaction progress led to compound **17** in 88% yield. Acetylation of **17** then afforded compound **18**. Stabilization of the glycosidic linkage by the installment of electron-withdrawing groups proved to be successful, and the treatment of **18** with 20% TFA in DCM resulted in compound **19** in 82% yield. During the reaction only a minimal amount of hydrolysis was observed. The synthesis was continued by the treatment of **19** with ammonia in MeOH and ensuing purification of the crude product using HW40 gel filtration to result in reference compound **20** in 87% yield.

**Scheme 2:**
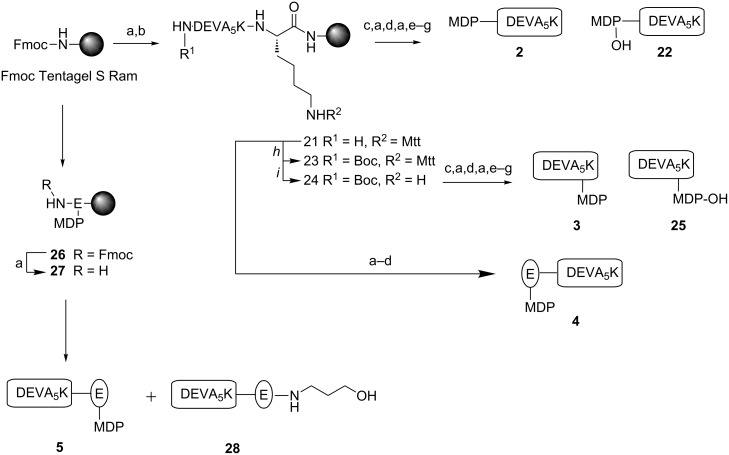
a) 20% piperidine, NMP; b) Fmoc SPPS DEVA_5_K; c) Fmoc-D-isoGln-OH, HCTU, DIPEA, NMP; d) Fmoc-L-Ala-OH, HCTU, DIPEA, NMP; e) **10**, HATU, DIPEA, NMP; f) 95% TFA, 2.5% H_2_O, 2.5% TIS; g) Boc_2_O, NMP, DIPEA; h) 3% TFA, DCM. i) **16**, HATU, DIPEA, NMP; j) **16**, DIPEA, HCTU, DMSO, DMF.

With MurNAc building block **10** and **16** in hand, the solid-phase peptide synthesis of the MDP-antigen conjugates **2–5** was undertaken (Scheme 2). Commercially available Fmoc protected amino acids equipped with standard acid labile protective groups were used. The side chain of the C-terminal lysine of the antigenic peptide was protected with the methyl trityl (Mtt) protective group, allowing the modification of both the N- or C-terminal end at the final stage of the synthesis. In a standard elongation cycle using HCTU as a coupling reagent, acetic anhydride as capping reagent and piperidine to remove the Fmoc-group, immobilized peptide **21** was assembled using fully automated peptide synthesis (Scheme 2). To obtain conjugate **2** with MDP on the N-terminus of the peptide, peptide **21** was consecutively elongated with Fmoc-D-isoglutamine, Fmoc-L-alanine and MurNAc **10**. The coupling of MurNAc **10** involved a double coupling protocol, using HATU instead of HCTU as condensing agent. The obtained fully protected and immobilized precursor was treated with a cocktail of 95% TFA, 2.5% TIS and 2.5% H_2_O to give, after precipitation in Et_2_O, conjugate **2** and the hydrolyzed conjugate **22** in a 1:1 ratio. Purification by RP-HPLC resulted in the isolation of pure conjugate **2** (2% overall yield).

In the next target conjugate **3**, MDP occupies the C-terminal position of the antigenic peptide. To obtain **3**, the N-terminus of immobilized peptide **21** was protected with a Boc-group by treatment of **21** with 1 M Boc_2_O in NMP and two equivalents of DIPEA (Scheme 2). Next, the resin was treated with a solution of 3% TFA in DCM to selectively remove the Mtt protective group from the side chain of the C-terminal lysine. The resulting free amine was consecutively elongated with Fmoc-D-isoglutamine, Fmoc-L-alanine and MurNAc **10** as described for conjugate **2**. Subsequently, the resin was subjected to the cleavage cocktail (95% TFA, 2.5% TIS and 2.5% H_2_O) to give conjugate **3** and the hydrolyzed conjugate **25** in a 1:1 ratio. Purification by RP-HPLC gave pure conjugate **3** (2% overall yield).

Conjugate **4** was obtained by the application of advanced building block **16** (Scheme 1). En route to **4**, peptide **21** was elongated with **16** in a coupling cycle using HATU as a coupling reagent (Scheme 2). The solid-phase synthesis scheme was concluded with the removal of the Fmoc protective group resulting in the partially protected immobilized precursor of **4**. Acidic cleavage of all protecting groups and release of the target conjugate from the resin gave, after precipitation of the crude product from Et_2_O at –20 °C and RP-HPLC purification, pure conjugate **4** in 2% overall yield.

To synthesize the C-terminal functionalized conjugate **5**, Tentagel S RAM resin was first condensed with building block **16**, which turned out to be challenging. Changing the solvent mixture from pure NMP to 20% DMSO in NMP led to **26** with a moderate coupling efficiency of 56% as judged from an Fmoc-cleavage test of an aliquot of resin. The Fmoc protective group in **26** was removed and the resin was elongated by automated SPPS with the DEVA_5_K motif resulting in the immobilized fully protected conjugate. The same deprotection and cleavage conditions as described for **4** were used to isolate conjugate **5**. Treatment of the resin with the cleavage cocktail for a shorter reaction time reduced the anomeric hydrolysis of the conjugate. Purification by RP-HPLC gave pure conjugate **5** in 6% overall yield.

### Immunological evaluation of the MDP-antigen conjugates

The immunostimulatory activity of conjugates **2**–**5** was assessed in three different assays. In these assays, besides reference compound **20**, also the unconjugated peptide, DEVA_5_K (**29**), and the Pam3Cys-antigen conjugate **30** were used as relevant control compounds [13]. In the first assay the NOD2 stimulatory potential of the conjugates was assessed. In the second and third assay the conjugates were evaluated for their potential to induce maturation of dendritic cells (DCs) and their ability to support uptake followed by antigen processing and presentation by DCs. The MHC class I molecule K^b^ on DCs derived from C57BL/6 mice is able to present the SIINFEKL epitope (OVA_257-264_), which is embedded in the antigenic long peptides. Presentation of the SIINFEKL-antigen can be measured by adding a CD8^+^ T cell hybridoma (B3Z), which is specific for the SIINFEKL epitope. This hybridoma contains a lacZ reporter construct linked to the nuclear factor of activated T cells (NFAT), enabling colorimetric measurement of the level of T cell receptor mediated activation of this T cell clone.

**Figure 2:**
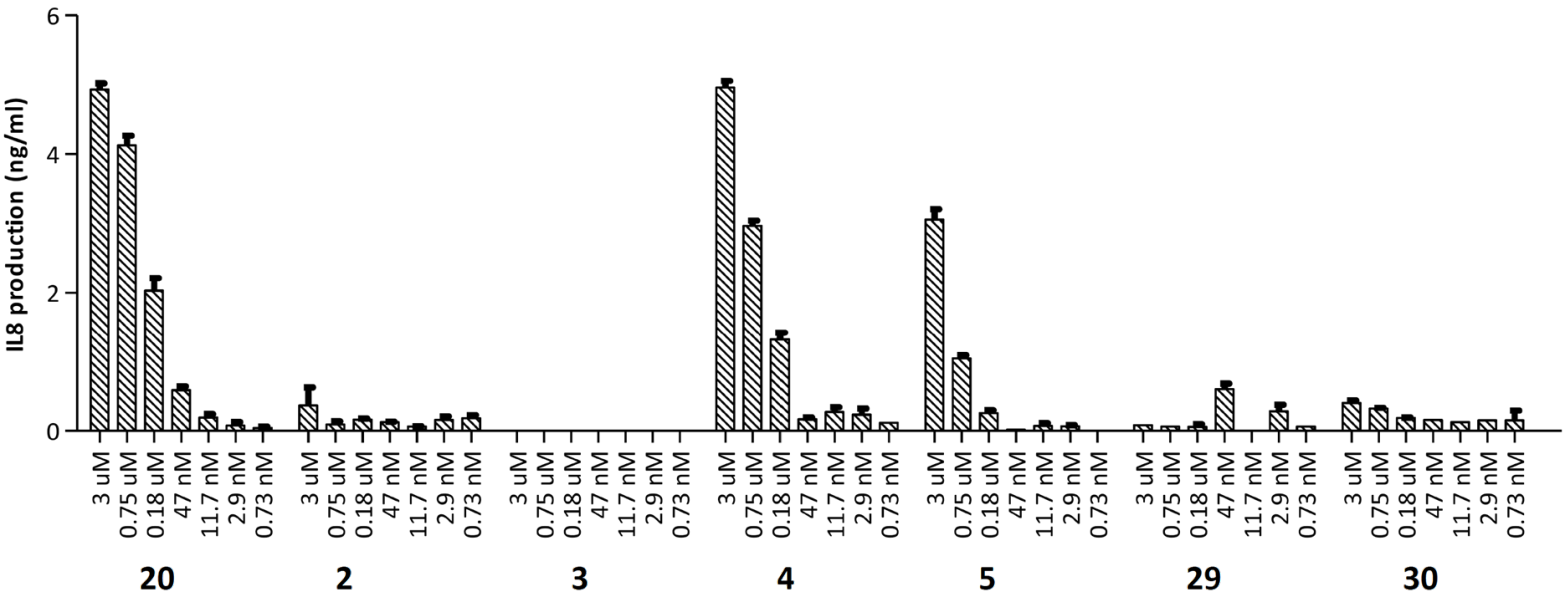
Potency of the NOD2-L antigen conjugates **2**–**5** in NOD2 transfected HEK cells.

The NOD2 activating capacity of conjugates **2**–**5** was tested using a stable NOD2 transfected human embryonic kidney (HEK293) cell line, in which the level of interleukin 8 (IL-8) production reflects the NOD2 stimulatory capacity of the compounds (Figure 2). MDP derivative **20** showed a substantial amount of IL-8 production, confirming that the β-azidopropanol modification on the anomeric position is allowed [43–44]. Of conjugates **2**–**5** the conjugates **2** and **3** showed activity close to background levels. In contrast, conjugates **4** and **5** induced IL-8 production at a level comparable to MDP derivative **20**. The difference in the activity of conjugates **2** and **3** compared to **4** and **5** indicates that the attachment point of the MDP to the antigenic peptide is important. Conjugation at the GlcNAc anomeric center of MDP as in conjugates **4** and **5** is more favorable than conjugation to the side chain of the L-isoglutamic acid residue (**2**, **3**). The negative controls, peptide **29** and conjugate **30**, showed no activity indicating that the activities of **4** and **5** are a consequence of the presence of the MDP in these conjugates. Notably, the location of the ligand on either the C- or N-terminus of the peptide (**4** vs **5**) does not seem to affect the NOD2 stimulating activity significantly, although the N-terminal conjugate appears slightly more active than the C-terminal conjugated MDP.

**Figure 3:**
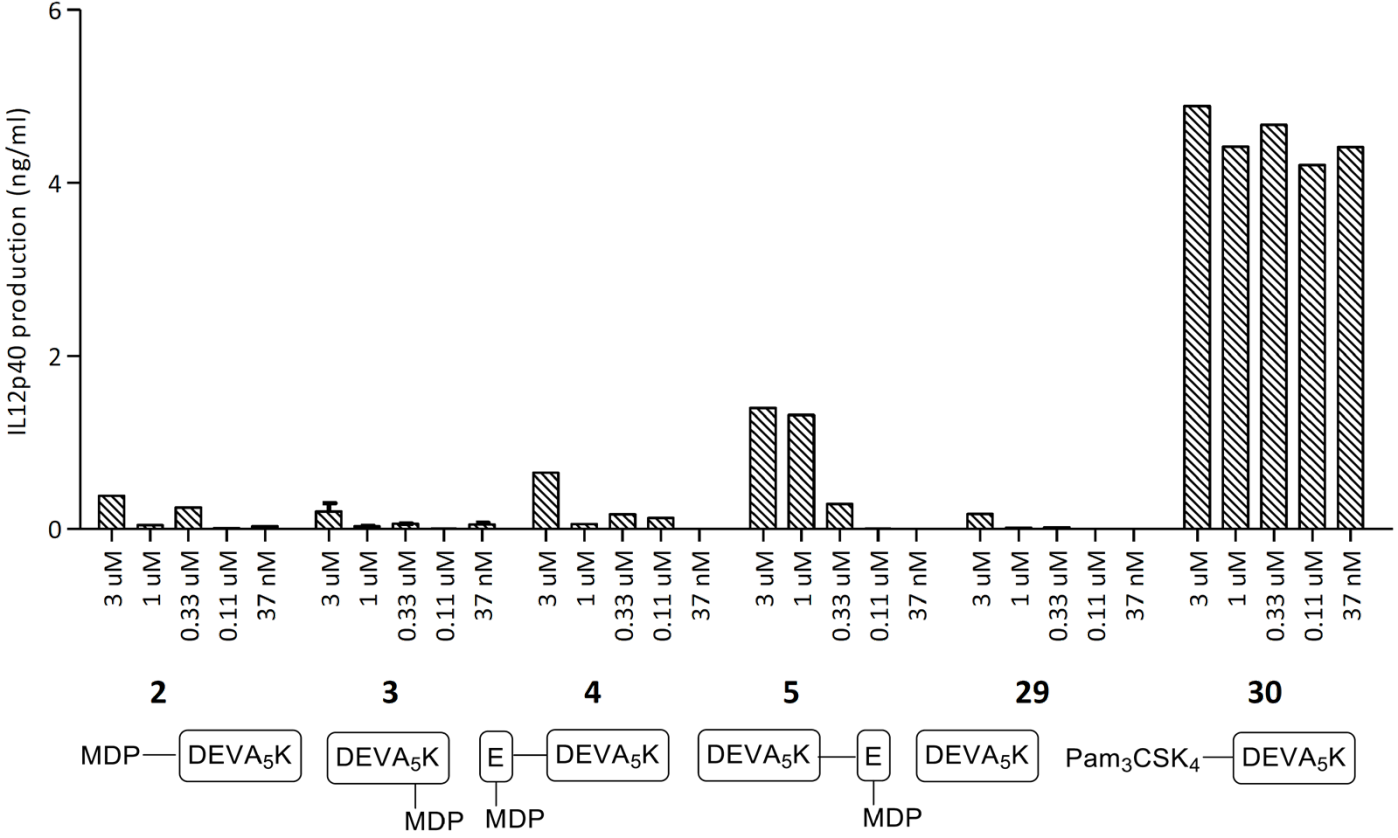
DC activation of the conjugates **2**–**5** and reference compounds **29** and **30**.

The ability of conjugates **2**–**5** to induce DC maturation was evaluated by measuring interleukin 12 (IL-12p40) production by the cultured murine DC line, D1 [45]. IL-12 secretion by DCs is a good indicator for the immunostimulatory potential of a compound, because this cytokine is essential for T cell stimulation and differentiation upon presentation by these antigen presenting cells. In Figure 3 it is shown that conjugates **2**–**5** produce amounts of IL-12 that are close to background level. In agreement with the results of the NOD2 transfected HEK cells conjugates **4** and especially **5** are more active than **2** and **3** indicating that the MDP ligation locus affects the activity of the constructs. Notably, reference Pam3Cys-conjugate **30** is much more potent than MDP-conjugates **4** and **5**. A possible explanation for the lack of strong maturating activity of the conjugates **4** and **5** is that these conjugates are not effectively internalized and therefore only a very low concentration reaches the cytosol resulting in a poor stimulation of the NOD2 receptor [46].

**Figure 4:**
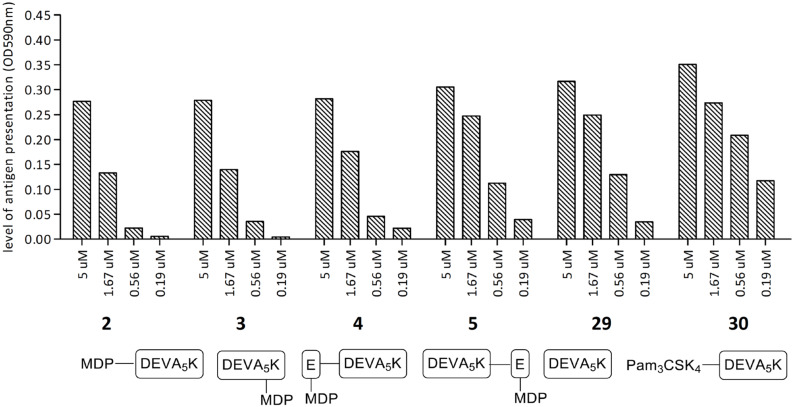
Antigen presentation of the conjugates **2**–**5** and reference compounds **29** and **30**.

Finally, the influence of the MDP-modification on the MHC I-mediated presentation of the antigenic peptide was investigated using the SIINFEKL-specific T-cell hybridoma (B3Z) assay. The DCs were treated with constructs **2**–**5** and reference compounds **29** and **30** (Figure 4). All constructs gave rise to antigen presentation, which indicates that the presentation is not affected by the condensation of an MDP to either C- or N-terminus of the antigenic peptide. Known conjugate **30** containing TLR-2 ligand Pam_3_CSK_4_ showed, as previously reported, an increased level of antigen presentation in comparison to peptide **29** [13].

Collectively, the data obtained from the assays described indicate that the MDP-based conjugates **2–5** are poor maturating agents for murine dendritic cells while antigen presentation from the peptide part of the constructs is retained. Since the NOD2 activity of constructs **4** and **5** is intact as determined by the assay using the NOD-2 transfected cell line, the overall failure of the described construct to mature DCs is probably not caused by the lack of receptor binding. The NOD2 receptor is located in the cytosol and the mechanisms underlying NOD2 ligand internalization and processing, before reaching the receptor in the cytosol are not yet fully understood [46]. There are indications that PG fragments are internalized via endocytosis and subsequently processed and transported to the cytosol. Because the antigen presentation assay indicates that the NOD2 modified peptides are processed correctly and presented by the MHC class I molecules, we assume that the poor activity of the MDP-conjugates is not (solely) due to the inability of these to reach the cytosol of the DC. Presumably the NOD2 triggering by MDP in the used murine DC cell line is comparatively ineffective as compared to the NOD2 transfected HEK cells.

## Conclusion

We have described the assembly of two MDP building blocks, suitable for automated solid-phase peptide synthesis, and their application in the construction of a set of four MDP-peptide conjugates. These conjugates comprised a C- or N-terminal ligation of the MDP to the antigenic SIINFEKL peptide via either the L-isoglutamine function or the C-1 of MDP. During the synthesis the glycosidic bond of MDP turned out to be sensitive to acidic deprotection conditions, resulting in partial hydrolysis of the aglycon. Nevertheless, pure MDP derivatives and conjugates could be isolated. The NOD2 stimulatory potential critically depended on the mode of ligation. Where the constructs that were conjugated via the anomeric center of MDP showed good NOD2 activation, the conjugates linked to the antigenic peptide through the L-isoglutamine residue were devoid of such stimulating capacity. Conjugation of the MDP-ligands to the antigenic peptide did not hamper the antigen presentation, although the conjugates only showed a marginal DC maturation potential. This indicates that the conjugates can be taken up by DCs and properly processed, but that the NOD2 ligand does not trigger the receptor effectively enough to induce maturation of these murine DCs. It is known however, that there are differences in NOD sensitivity between human and murine DCs. To increase the potency of NOD2 ligands, lipophilic MDP derivatives have been developed and it might well be that these can display a more favorable uptake profile than the relatively polar MDP-conjugates (**2**–**5**) described here. Conjugates of these lipophilic MDPs can therefore be attractive vaccine modalities and efforts are currently underway in our laboratory to investigate these.

## Supporting Information

File 1 Full experimental details and characterization of all new compounds.

File 2 NMR spectra.
