# Supplementary material for: Design, automated synthesis and immunological evaluation of NOD2-ligand–antigen conjugates
Source: Beilstein J Org Chem. 2014 Jun 26;10:1445–53. doi: 10.3762/bjoc.10.148 (PMC4077378; doi:10.3762/bjoc.10.148)
Supplement: File 1 — Full experimental details and characterization of all new compounds. [file Beilstein_J_Org_Chem-10-1445-s001.pdf]

Supporting Information File 1

for

Design, automated synthesis and immunological evaluation of NOD2-ligand–antigen conjugates

Marian M. J. H. P. Willems[1], Gijs G. Zom[2], Nico Meeuwenoord[1], Ferry A. Ossendorp[2], Herman S. Overkleeft[1], Gijsbert A. van der Marel[1], Jeroen D. C. Codée[1*] and Dmitri V. Filippov[1*]

Address: [1]Leiden Institute of Chemistry, Leiden University, P.O. Box 9502, 2300 RA Leiden, The Netherlands and [2]Department of Immunohematology and Blood Transfusion, Leiden University Medical Centre, P. O. Box 9600, 2300 RC Leiden, The Netherlands

Email: Jeroen D. C. Codée - jcodee@chem.leidenuniv.nl; Dmitri V. Filippov - filippov@chem.leidenuniv.nl

*Corresponding author

Full experimental details and characterization of all new compounds

Experimental section

All reagents and solvents used in the solid phase peptide synthesis were purchased from Bachem and Biosolve and used as received. Palmitoyl-Cys((*RS*)-2,3-di(palmityloxy)propyl)-OH was purchased from Bachem, Fmoc-amino acids from

Novabiochem and HATU from Tebu Bio. Tentagel based resins were ordered from Rapp Polymere. Light petroleum ether with a boiling range of 40–60 °C was used. All other solvents used under anhydrous conditions were stored over 4 Å molecular sieves except for methanol, which was stored over 3 Å molecular sieves. Solvents, used for work–up and silica gel column chromatography were of technical grade and distilled before use. All other solvents were used without further purification. Reactions were monitored by TLC–analysis or LC/MS analysis. LC/MS was conducted on a JASCO system using an Alltima C_{18} analytical column (4.6 × 50 mm, 5 µm particle size, flow 1.0 mL/min), Alltima CN analytical column (4.6 × 50 mm, 3 µm particle size, flow 1.0 mL/min) or a Alltima C_4 analytical column (4.6 × 50 mm, 5 µm particle size, flow 1.0 mL/min). Absorbance was measured at 214 and 256 nm. Solvent system: A: 100% water, B: 100% MeCN, C: 1% aq. TFA. Gradients of MeCN in 10% C were applied over 15 minutes unless stated otherwise. Purifications were conducted on the Gilson GX-281 preparative RP-HPLC system, supplied with a semipreparative Alltima CN column (10 × 250 mm, 5 µm particle size, flow 5.0 mL/min.) or semi preparative Alltima C_4 column (10 × 250, 5 µm particle size, flow 5.0 mL/min.). Solvent system: A: 0.1% aq. TFA and B: MeCN. Gradients of 10–90% MeCN were applied over 3 CV over 15 min unless stated otherwise. The UV absorption was measured at 214 and 256 nm. High resolution mass spectra were recorded by direct injection (2 µL of a 2 µM solution in H_2O/MeCN; 50/50: v/v and 0.1% formic acid) on a mass spectrometer Thermo Finnigan LTQ Orbitrap equipped with an electrospray ion source in positive mode (source voltage 3.5 kV, sheath gas flow 10, capillary temperature 523 K) with resolution R = 60000 at m/z 400 (mass range *m*/*z* = 150–2000) and dioctylphthalate (*m*/*z* = 391.28428) as lock mass. Optical rotations were measured on a Propol automatic polarimeter (sodium D–line, λ = 589 nm). Specific rotations $[\alpha]_D$ are given in degree per centimeter and the concentration *c* is given in mg/mL in the specific solvent. Maturation and B3Z presentation results were analyzed with Graphpad Prism version 5.01 for Windows, GraphPad Software, San Diego California USA. IR spectra were recorded on a Perkin Elmer Paragon 1000 FTIR spectrometer.

3-Azidopropyl 2-acetamido-3,4,6-tri-*O*-acetyl-2-deoxy-β-D-glucopyranoside (7)

Oxazoline **6** (4.4 g, 10 mmol) and azidopropanol (4.6 g, 46 mmol) were dissolved in freshly distilled DCM (75 mL, 0.13 M) and stirred over molecular sieves under argon

atmosphere at rt for 40 minutes. TMSOTf (1.7 mL, 9.4 mmol) was added portion-wise over 5 days. To monitor the progress of the reaction a sample of the crude reaction mixture was concentrated and taken up in $CDCl_3$ to analyse the sample by NMR spectroscopy. Upon completion, the reaction was quenched with TEA, filtered over Celite® and purified by flash chromatography (1:1 → 7:3 EtOAc:PE) to yield compound **7** as a white amorphous solid (3.72 g, 8.63 mmol, 83%). R_f = 0.4 (9 : 1 EtOAc : MeOH); $[\alpha]_D$ = -0.05 (c = 1, DCM); ^{1}H NMR (400 MHz, $CDCl_3$) δ 5.37 (t, J = 9.9 Hz, 1H, H-3), 5.06 (t, J = 9.6 Hz, 1H, H-4), 4.82 (d, J = 8.3 Hz, 1H, CH, H-1), 4.30 (dd, J = 12.2, 5.0 Hz, 1H, CH_2, H-6), 4.21 – 4.07 (m, 1H, CH_2, H-6), 4.01 – 3.88 (m, 2H, CH_2, $C_3H_6N_3$, CH, H-2), 3.87 – 3.83 (m, 1H, CH_2, $C_3H_6N_3$), 3.70 – 3.65 (m, , 1H, CH, H-5), 3.40 (t, J = 6.6 Hz, 2H, CH_2, $C_3H_6N_3$), 2.13 – 1.95 (m, 12H, CH_3, Ac, CH_3, NAc), 1.93 – 1.83 (m, J = 6.4 Hz, 2H, CH_2, $C_3H_6N_3$);^{13}C NMR (100 MHz, $CDCl_3$) δ 170.4 (C=O), 170.3 (C=O), 170.1 (C=O), 169.0 (C=O), 100.4 (CH, C-1), 72.1 (CH, C-3), 71.1 (CH, C-5), 68.6 (CH, C-4), 65.9 (CH_2, $C_3H_6N_3$), 61.9 (CH_2, C-6), 53.9 (CH, C-2), 47.6 (CH_2, $C_3H_6N_3$), 28.5 (CH_2, $C_3H_6N_3$), 20.7 (CH_3, NAc), 20.3 (CH_3, Ac), 20.2 (CH_3, Ac), 20.2 (CH_3, Ac); IR (cm^{-1}) 3275, 2098, 1745, 1639, 1224; HRMS [M+H^+] Calcd. for $C_{17}H_{26}N_4O_9$ 431.17705, found 431.17725.

3-Azidopropyl 2-acetamido-2-deoxy-β-D-glucopyranoside (8)

Compound **7** (31.6 g, 73.4 mmol) was dissolved in MeOH (750 mL, 0.1 M) and NaOMe (0.71 g, 13 mmol) was added. The resulting solution was stirred at rt for 20 h. The reaction mixture was quenched with Amberlite® H^+ resin. Filtration and concentration in vacuo yielded compound **10** as a white amorphous solid (22 g, 73 mmol). R_f = 0.1 (9 : 1 EtOAc : MeOH); $[\alpha]_D$ = -0.07 (c = 1, MeOH);
^{1}H NMR (400 MHz, MeOD) δ 4.35 (d, J = 8.4 Hz, 1H, CH, H-1), 3.94 – 3.81 (m, 2H, CH_2 H-6, CH_2, $C_3H_6N_3$), 3.63 – 3.47 (m, 3H, CH, H-3, CH_2 H-6, CH_2, $C_3H_6N_3$), 3.40-3.18 (m, 5H, CH, H-4, CH, H-5, CH, H-2, CH_2, $C_3H_6N_3$) 1.95 (s, 3H, CH_3, NAc), 1.78 – 1.75 (m, 2H, CH_2, $C_3H_6N_3$); ^{13}C NMR (100 MHz, MeOD) δ 173.7 (C=O), 102.8 (CH, C-1), 77.9 (CH, C-5), 76.0 (CH, C-3), 72.1 (CH, C-4), 67.1 (CH_2O, $C_3H_6N_3$), 62.8 (CH_2, C-6), 57.3 (CH, C-2), 49.0 (CH_2, CH_2N_3), 30.1 (CH_2, $C_3H_6N_3$), 23.0 (CH_3, NAc); IR (cm^{-1}): 3255, 2092, 1651, 1552; HRMS Calcd. for $[C_{11}H_{20}N_4O_6+ H]^+$ 305,14556, found 305,14575.

3-Azidopropyl 2-acetamido-4,6-*O*-benzylidene-2-deoxy-β-D-glucopyranoside (9)

Compound **8** (2.7 g, 8.6 mmol) was co-evaporated with DMF and dissolved in dry MeCN/DMF (3:1, 90 mL, 0.1 M). Benzaldehyde dimethyl acetal (1.9 mL, 13 mmol) and CSA (0.40 g, 1.7 mmol) were added and the resulting solution was stirred at rt for 18 h. The reaction was quenched with TEA, concentrated in vacuo and crystallized (DCM, MeOH, PE). Compound **9** was obtained as a white solid (2.90 g, 7.39 mmol, 86%); R_f = 0.6 (1 : 9 MeOH : DCM); $[\alpha]_D$ = -0.27 (c = 1, 1 : 1 DCM : MeOH); ^{1}H NMR (400 MHz, DMSO-D_6) 7.86 (d, J = 8.8. Hz, NH), 7.49 – 7.35 (m, 5H, CH, Ar), 5.60 (s, 1H, CH, benzylidene acetal), 5.33 (d, J = 4.9 Hz, 1H, NH), 4.46 (d, J = 8.4 Hz, 1H, H-1), 4.20 (dd, J = 10.1, 4.8 Hz, 1H, H-6), 3.81 – 3.68 (m, 2H, CH_2, H-6, CH_2, $C_3H_6N_3$), 3.66 – 3.28 (m, 6H, CH, H-3, CH, H-4, CH, H-5, CH_2, $C_3H_6N_3$), 1.83 (s, 3H, CH_3, NAc), 1.77 – 1.67 (m, 2H, CH_2, $C_3H_6N_3$); ^{13}C NMR (100 MHz, DMSO-D_6) δ 169.4 (C=O), 137.8 (C_q, CHPh), 128.1 (CH, CHPh), 126. 5 (CHPh), 101.7 (CH, CHPh), 100.8 (CH, C-1), 81.3 (CH, C-3), 70.5 (CH, C-5), 67.9 (CH_2, C-6), 66.0 (CH, C-4), 65.7 (CH_2, $C_3H_6N_3$), 56.2 (CH, C-2), 47.5 (CH_2, $C_3H_6N_3$), 28.6 (CH_2, $C_3H_6N_3$), 23.1 (CH_3, NAc); IR (cm^{-1}): 3275, 2870, 2100, 1624, 1552; HRMS Calcd. for $[C_{18}H_{24}N_4O_6 + H]^+$ 393.17686 found 393.17673.

3-Azidopropyl 2-acetamido-4,6-*O*-benzylidene-2-deoxy-3-*O*-((*R*)-1-carboxyethyl)-β-D-glucopyranoside (10)

Compound **9** (0.8 g, 2.0 mmol) was suspended in 1,4-dioxane (30 mL, 0.07 M) and dissolved upon heating to 95 °C. NaH (0.32 g, 60% in oil, 8.0 mmol) was added and the resulting solution was stirred at 95 °C for 1 h. The solution was cooled to 65 °C and a stock solution of (*S*)-2-chloropropanoic acid (0.26 mL, 3.0 mmol, in 5 mL 1,4-dioxane) was added. The solution was stirred at 65 °C for 1.5 h. The reaction was quenched with MeOH and the pH was adjusted to pH ~ 3 with 1 M HCl. The product was extracted with DCM, dried ($MgSO_4$) and concentrated in vacuo. Crystallization ($CHCl_3$/EtOAc/PE) yielded compound **10** as an off-white solid (0.64 g, 1.4 mmol, 69%). R_f = 0.43 (1 : 9 MeOH : EtOAc); $[\alpha]_D$ = -0.21 (c = 1, MeOH); ^{1}H NMR (400 MHz, 1 : 1 MeOD : $CDCl_3$) δ 7.51 – 7.45 (m, 2H, CH, Ar), 7.41 – 7.45 (m, 3H, CH, Ar), 5.59 (s, 1H, CH, benzylidene acetal), 4.57 (d, J = 8.4 Hz, 1H, CH, H-1), 4.45 (dd, J = 7.2 Hz, 14.0 Hz, 1H, CH, lactic acid), 4.34 (dd, J = 4.8 Hz, 10.4 Hz, 1H, CH_2, H-6), 3.97 -3.91 (m, 1H, CH_2, $C_3H_6N_3$), 3.86 – 3.78 (m, 2H, H3, H6), 3.72 – 3.68 (m,

2H, CH, H2, H4), 3.61 – 3.56 (m, 1H, CH_2, $C_3H_6N_3$), 3.48 – 3.41 (m, 1H, CH, H-5), 3.40 (t, J = 6.4 Hz, 2H, CH_2, $C_3H_6N_3$), 2.03 (s, 3H, CH_3, NAc), 1.89 – 1.78 (m, 2H, CH_2, $C_3H_6N_3$), 1.40 (d, J = 6.8 Hz, 3H, CH_3, lactic acid); ^{13}C NMR (100 MHz) δ 175.8 (C=O), 172.4 (C=O), 136.9 (C_q, Ar), 128.8 (CH, Ar), 128.0 (CH, Ar), 125.6 (CH, Ar), 102.0 (CH, benzylidene acetal), 101.0 (CH, C-1), 82.1 (CH, C-4), 77.1 (CH, C-3), 75.4 (CH, Lactic acid), 68.4 (CH_2, C-6), 66.2 (CH_2, $C_3H_6N_3$), 65.9 (CH, C-5), 55.4 (CH, C-2), 47.7 (CH_2, $C_3H_6N_3$), 28.7 (CH_2, $C_3H_6N_3$), 22.6 (CH_3 NAc), 18.5 (CH_3, lactic acid); IR (cm^{-1}): 3275, 2098, 1709, 1656, 1556; HRMS Calcd. for $[C_{21}H_{28}N_4O_8 + H]^+$ 465.19774, found 465.19799.

Fmoc-D-isoGln(O*t*-Bu)-NH_2 (12)

To a stirred solution of Fmoc-D-Glu(O*t*-Bu)-OH (0.9 g, 2.0 mmol) in 1,4-dioxane (20 mL, 0.1M) was added NH_4HCO_3 (0.71 g, 9.0 mmol), Boc_2O (0.58 g, 2.7 mmol) and pyridine (0.25 mL, 3.1 mmol). After 24 h the solution was diluted with EtOAc/H_2O and washed with water. The organic layer was dried ($NaSO_4$) and concentrated in vacuo. Crystallization (MeOH) yielded compound **12** (0.62 g, 1.5 mmol, 73%). R_f = 0.5 (7 : 3 EtOAc : PE); $[\alpha]_D$ = -0,6 (c = 1, $CHCl_3$); ^1H NMR (400 MHz, DMSO-D_6) δ 7.89 (d, J = 7.5 Hz, 2H, CH, Fmoc), 7.75 – 7.72 (m, 2H, CH, Fmoc), 7.43 – 7.28 (m, 4H, CH, Fmoc), 7.06 (s, 1H, NH_2), 4.34 – 4.17 (m, 3H, CH, Fmoc, CH_2, Fmoc), 3.97 – 3.94 (m, 1H, CH, α i-D-Gln), 2.22 (t, J = 7.8 Hz, 2H, CH_2, γ i-D-Gln), 1.94 – 1.84 (m, 1H, CH, β i-D-Gln), 1.78 – 1.67 (m, J = 13.7 Hz, CH, β i-D-Gln), 1.39 (s, 9H, CH_3, ^{t}Bu); ^{13}C NMR (100 MHz, DMSO-D_6) δ 173.4 (C=O), 171.7 (C=O), 156.0 (C=O), 143.8 (C_q, Fmoc), 140.7 (C, Fmoc), 127.7 (CH, Fmoc), 127.1 (CH, Fmoc), 125.4 (CH, Fmoc), 120.1 (CH, Fmoc), 79.7 (C_q, ^{t}Bu), 65.64 (CH_2, Fmoc), 53.7 (CH, α i-D-Gln), 46.7 (CH, Fmoc), 31.5 (CH_2, γ i-D-Gln), 27.8 (CH_3, ^{t}Bu), 27.3 (CH_2, β i-D-Gln); IR (cm^{-1}): 33387, 3329, 1720, 1689, 1532; LC/MS: Rt = 9.24 min (C_{18} Alltima, 10 – 90% MeCN, 15 min run); HRMS Calcd. for $[C_{24}H_{28}N_2O_5 + H]^+$ 425.20710, found 425.20706.

Fmoc-L-Ala-D-isoGln(O*t*-Bu)-NH_2 (13)

To a stirred solution of compound **12** (2.0 g, 4.8 mmol) in DCM (40 mL, 0.73 M) was added DBU (0.71 mL, 4.8 mmol). After 20 min HOBt (2.9 g, 21 mmol) was added. Subsequently Fmoc-Ala-OH (1.8 g, 5.7 mmol), EDC (1.1 g, 5.7 mmol) and DiPEA

(4.7 mL, 28 mmol) were added. The resulting solution was stirred for 18 h, washed with 1 M HCl (3 × 5 mL), sat. $NaHCO_3$ (3 × 5 mL), brine (3 × 5 mL), dried ($NaSO_4$) and concentrated in vacuo. Precipitation from EtOAc/PE resulted in compound **16** as a white solid (1.93 g, 3.89 mmol, 82%). R_f = 0.8 (8 : 2 EtOAc : PE); $[\alpha]_D$ = 0.8 (c = 1, $CHCl_3$); ^{1}H NMR (400 MHz, DMSO-D_6) δ 8.04 (d, J = 8.2 Hz, 1H, NH), 7.90 (d, J = 7.5 Hz, 2H, CH, Fmoc), 7.73 (t, J = 6.6 Hz, 2H, CH, Fmoc), 7.61 (d, J = 7.0 Hz, 1H, NH), 7.47 – 7.32 (m, 4H, CH, Fmoc), 7.26 (s, 1H, NH_2), 7.14 (s, 1H, NH_2), 4.34 – 4.12 (m, 4H, CH, α i-D-Gln, CH_2, Fmoc, CH, Fmoc), 4.08 – 4.04 (m, J = 16.4, 14.2, 7.1 Hz, 1H, α CH Ala), 2.18 (t, J = 7.8 Hz, 2H, γ CH_2, i-D-Gln), 1.99 – 1.94 (m, J = 14.4, 7.7 Hz, CH_2, β i-D-Gln), 1.72 – 1.69 (m, 1H CH_2, β i-D-Gln), 1.36 (s, 9H, CH_3, ^{t}Bu), 1.26 – 1.14 (m, 3H, CH_3, Ala); ^{13}C NMR (100 MHz, DMSO-D_6) δ 173.1 (C=O), 172.6 (C=O), 171.6 (C=O), 155.9 (C=O), 143.9 (C_q, Fmoc), 140.7 (C_q, Fmoc), 127.7 (CH, Fmoc), 127.1 (CH, Fmoc), 125.3 (CH, Fmoc), 120.1 (CH, Fmoc), 79.7 (C_q, ^{t}Bu), 65.7 (CH_2, Fmoc), 51.4 (CH, α i-D-Gln), 50.3 (CH, α Ala), 46.6 (CH, Fmoc), 31.2 (CH_2, γ i-D-Gln), 27.7 (CH_3, ^{t}Bu), 27.2 (CH_2, β i-D-Gln), 18.0 (CH_3, Ala); IR (cm^{-1}): 3286, 1641, 1541, 1257; LC/MS: Rt = 6.09 min (C_{18} Alltima, 0 – 50% MeCN, 15 min run); HRMS Calcd. for $[C_{27}H_{33}N_3O_6 + H]^+$ 496.24421, found 496.24396.

Muramyl dipeptide derivative 14

To a stirred solution of compound **13** (50 mg, 0.1 mmol) in DMF (2 mL, 0.08 M) was added DBU (14 µL, 90 µmol). After 10 min HOBt (56 mg, 0.42 mmol) was added. A mixture of compound **10** (35 mg, 77 µmol) in DMF (0.5 mL, 0.15 M), HATU (29 mg, 7.7 µmol) and DiPEA (80 µL, 0.46 mmol) was added to the solution. The resulting solution was stirred for 18 h at rt. The solution was diluted 10 times (DCM), washed with 1 M HCl (3 × 5 mL), sat. $NaHCO_3$ (3 × 5 mL), brine (3 × 5 mL), dried ($NaSO_4$) and concentrated. The crude compound was purified by flash chromatography (98:2 → 9:1 DCM : MeOH) to obtain title compound **14** as an amorphous solid (50 mg, 70 µmol, 70%). R_f = 0.6 (9 : 1 $CHCl_3$: MeOH); $[\alpha]_D$ = -11.4 (c = 0.44, 1 : 1 MeOH : $CHCl_3$); ^{1}H NMR (400 MHz, MeOD) δ 7.54 (d, J = 4.9 Hz, 1H, NHAc), 7.47 (m, 2H, CH, Ar), 7.42 – 7.31 (m, 3H, CH, Ar), 5.58 (s, 1H, CH, benzylidene acetal), 4.59 (d, J = 7.8 Hz, 1H, CH, H-1), 4.41 – 4.31 (m, 2H, CH, α i-D-Gln, CH_2, H-6), 4.31 – 4.21 (m, 1H, CH, α Ala), 4.14 (q, J = 6.7 Hz, 1H, CH, lactic acid), 3.99 – 3.89 (m, 1H, CH_2, $C_3H_6N_3$), 3.89 – 3.74 (m, 3H, CH, H-2, CH, H-3, CH_2, $C_3H_6N_3$), 3.71 – 3.55 (m, 2H, CH, H-4, CH_2, H-6), 3.52 – 3.42 (m, 1H, CH, H-5), 3.39 (t, J = 6.6 Hz, 2H, CH_2,

$C_3H_6N_3$), 2.35 – 2.31 (m, 2H, CH_2, γ i-D-Gln), 2.25 – 2.11 (m, 1H, CH_2, β i-D-Gln), 1.99 (s, 3H, CH_3, NAc), 1.95 – 1.75 (m, 3H, CH_2, $C_3H_6N_3$, CH_2, β i-D-Gln), 1.44 (s, 9H, CH_3, ^{t}Bu), 1.40 (d, J = 7.1 Hz, 3H, CH_3, lactic acid), 1.36 (d, J = 6.7 Hz, 3H, CH_3, Ala); ^{13}C NMR (100 MHz, MeOD) δ 175.0 (C=O), 174.8 (C=O), 173.7 (C=O), 173.2 (C=O), 172.7 (C=O), 137.6 (C_q, Ar), 129.5 (CH, Ar), 128.7 (CH, Ar), 126.4 (CH, Ar), 102.1 (CH, benzylidene acetal), 101.9 (CH, C-1), 81.6 (C_q, ^{t}Bu), 81.5 (CH, C-4), 79.7 (CH, C-3), 78.3 (CH, lactic acid), 69.0 (CH_2, C-6), 66.8 (CH_2, $C_3H_6N_3$), 66.5 (CH, C-5), 56.3 (CH, C-2), 52.7 (CH, α i-D-Gln), 49.9 (CH, α Ala), 48.4 (CH_2, $C_3H_6N_3$), 32.2 (CH_2, γ i-D-Gln), 29.4 (CH_2, $C_3H_6N_3$), 28.2 (CH_3, ^{t}Bu), 27.5 (CH_2, β i-D-Gln), 23.2 (CH_3, NAc), 19.6 (CH_3, lactic acid), 17.7 (CH_3, Ala); IR (cm^{-1}): 3286, 2094, 1647, 1535; LC/MS: Rt = 7.62 min (C_{18} Alltima, 10 – 90% MeCN, 15 min run); HRMS Calcd. for [$C_{33}H_{49}N_7O_{11}$ + H]$^+$ 720.35628, found 720.35639.

Muramyl dipeptide derivative 15

Compound **14** (2.7 g, 3.8 mmol) was co-evaporated with DMF and dissolved in DMF (25 mL), diluted with THF (12 mL, 0.1 M) and the reaction mixture was stirred for 3 h with PMe_3 (7.5 mL, 1M in THF). The solution was concentrated in vacuo yielding the free amine (2.1 g, 3.0 mmol, 80%). R_f = 0.2 (8 : 2 $CHCl_3$: MeOH +2% AcOH); $[\alpha]_D$ = -19 (c = 0.19, 1 : 1 $CHCl_3$: MeOH); ^{1}H NMR (400 MHz, DMSO-D_6) δ 8.31 (s, 1H, NH), 8.12 (d, J = 8.2 Hz, 1H, NH), 8.02 (d, J = 9.1 Hz, 1H, NH), 7.94 (s, 1H, NH_2), 7.73 (t, J = 3.7 Hz, 1H, NH), 7.49 – 7.30 (m, 5H, CH, Ar), 7.11 (s, 1H, NH_2), 5.75 (s, 1H, CH, benzylidene acetal), 4.47 (d, J = 8.3 Hz, 1H, CH, H-1), 4.27 – 4.19 (m, 2H, CH, α i-D-Gln, CH_2, H-6), 4.18 – 4.04 (m, 2H, CH, lactic acid, CH, Ala), 3.82 – 3.72 (m, 3H, CH, H-2, CH_2, H-6, CH_2, $C_3H_6N_3$), 3.69 – 3.38 (m, 4H, CH, H-3, CH, H-4, CH, H-5, CH_2, $C_3H_6N_3$), 2.63 (t, J = 6.9 Hz, 2H, CH_2, $C_3H_6N_3$), 2.17 (t, J = 7.9 Hz, 2H, CH_2, γ i-D-Gln), 1.99 – 1.87 (m, 1H, CH_2, β i-D-Gln), 1.82 (s, 3H, NAc), 1.74 – 1.55 (m, 1H, CH_2, β i-D-Gln), 1.36 (s, 9H, CH_3, ^{t}Bu), 1.26 – 1.18 (m, 6H, CH_3, lactic acid, CH_3, Ala); ^{13}C NMR (100 MHz, DMSO-D_6) δ 173.17 (C=O), 172.14 (C=O), 171.86 (C=O), 171.67 (C=O), 137.63 (C_q, Ar), 128.73 (CH, Ar), 128.21 (CH, Ar), 127.90 (CH, Ar), 125.92 (CH, Ar), 101.54 (CH, C-1), 100.20 (CH, benzylidene acetal), 80.37 (CH, C-3), 79.77 (C_q, ^{t}Bu), 79.09 (CH, C-4), 77.38 (CH, lactic acid), 67.87 (CH_2, C-6), 66.91 (CH_2, $C_3H_6N_3$), 65.69 (CH, C-5), 54.73 (CH, C-2), 51.54 (CH, α i-D-Gln), 48.35 (CH, Ala), 37.84 (CH_2, $C_3H_6N_3$), 31.29 (CH_2, $C_3H_6N_3$), 31.07 (CH_2, β i-D-Gln), 27.78 (CH_3, ^{t}Bu), 27.22 (CH_2, γ i-D-Gln), 23.09 (CH_3, NAc), 19.08

(CH_3, lactic acid), 18.27 (CH_3, Ala); IR (cm^{-1}): 3267, 1724, 1639, 1539, 1369; LC/MS: Rt = 5.66 min (Alltima C_{18}, 10 – 90% MeCN, 15 min run); HRMS Calcd. for $[C_{33}H_{51}N_5O_{11} + H]^+$ 694.36578, found 694.36615.

To the primary amine (2.1 g, 2.9 mmol), dissolved in DMF (39 mL, 0.07 M), were added Fmoc-Glu-OAllyl (1.3 g, 3.2 mmol), HCTU (1.3 g, 3.2 mmol) and DiPEA (1.4 mL, 8.7 mmol). The solution was stirred for 18 h and concentrated in vacuo. Precipitation from $CHCl_3$ /MeOH/Et_2O yielded the MDP derivative **15** (1.8 g, 1.7 mmol, 57%). R_f = 0.8 (8 : 2 $CHCl_3$: MeOH); $[\alpha]_D$ = -23 (c = 0.28, 1 : 1 $CHCl_3$: MeOH); ^{1}H NMR (400 MHz, DMSO-D_6) δ 8.09 (d, J = 8.3 Hz, 1H, NH), 7.95 – 7.75 (m, 5H, CH, Ar, NH), 7.71 (d, J = 7.4 Hz, 2H, CH, Ar), 7.48 – 7.26 (m, 2H, CH, Fmoc), 7.09 (s, 1H, NH_2, i-D-Gln) 5.96 – 5.80 (m, 1H, CH, allyl), 5.68 (s, 1H, CH, benzylidene acetal), 5.35 – 5.16 (m, 2H, CH_2, allyl), 4.57 (d, J = 5.2 Hz, 2H, CH_2 allyl), 4.45 (d, J = 8.3 Hz, 1H, CH, H-1), 4.36 – 4.18 (m, 5H, CH_2, H-6, CH, lactic acid, CH_2, Fmoc, CH, Fmoc), 4.18 – 4.09 (m, 1H, CH, α Glu), 4.10 – 4.00 (m, 2H, CH, Ala, CH, α i-D-Gln), 3.81 – 3.71 (m, J = 18.4, 9.0 Hz, 3H, CH_2, $C_3H_6N_3$, CH_2, H-6, CH, H-2), 3.69 – 3.57 (m, 2H, CH, H-3, CH, H-4), 3.49 – 3.28 (under H_2O signal, CH_2, $C_3H_6N_3$, CH, H-5), 3.19 – 2.94 (m, 2H, CH_2, $C_3H_6N_3$), 2.24 – 2.10 (m, 4H, CH_2, γ i-D-Glu, CH_2, γ Glu), 2.05 – 1.86 (m, 2H, CH_2, β i-D-Glu, CH_2, β Glu), 1.91 – 1.73 (m, 4H, CH_3, NAc, CH_2, β i-D-Gln), 1.73 – 1.63 (m, 1H, CH_2, β Glu), 1.58 (t, J = 6.4 Hz, 2H, CH_2, $C_3H_6N_3$), 1.36 (s, 9H, CH_3, ^{t}Bu), 1.24 – 1.19 (m, J = 16.7, 6.8 Hz, 6H, CH_3, lactic acid, CH_3, Ala); ^{13}C NMR (100 MHz, DMSO-D_6) δ 173.1 (C=O), 172.1 (C=O), 171.9 (C=O), 171.8 (C=O), 171.6 (C=O), 171.1 (C=O), 169.9 (C=O), 156.2 (C=O), 143.8 (C_q), 140.8 (C_q), 137.6 (C_q), 132.4 (CH, allyl), 128.8 (CH, Ar), 128.2 (CH, Ar), 127.7 (CH, Ar), 127.2 (CH, Ar), 125.9 (CH, Ar), 125.3 (CH, Ar), 120.2 (CH, Ar), 117.8 (CH_2, allyl), 101.6 (CH, C-1), 100.2 (CH, benzylidene acetal), 80.4 (CH, C-3), 79.7 (C_q, ^{t}Bu), 79.1 (CH, C-4), 77.4 (CH, lactic acid), 67.8 (CH_2, H-6), 66.9 (CH_2, $C_3H_6N_3$), 65.8 (CH_2, Fmoc), 65.7 (CH, C-5), 64.9 (CH_2, allyl), 54.7 (CH, C-2), 53.6 (CH, α i-D-Gln), 51.5 (CH, α Glu), 48.7 (CH, Fmoc), 46.7 (CH, Ala), 35.6 (CH_2, $C_3H_6N_3$), 31.6 (CH_2, γ i-D-Gln), 31.3 (CH_2, γ Glu), 29.3 (CH_2, $C_3H_6N_3$), 27.8 (CH_3, ^{t}Bu), 27.2 (CH_2, β, Glu), 26.7 (CH_2, β i-D-Gln), 23.1 (CH_3, NAc), 19.1 (CH_3, lactic acid), 18.2 (CH_3, Ala); IR (cm^{-1}): 3278, 1728, 1639, 1539, 1369; LC/MS: Rt = 9.20 min (Alltima C_{18}, 10 – 90% MeCN, 15 min run); HRMS Calcd. for $[C_{56}H_{72}N_6O_{16} + H]^+$ 1085.50776, found 1085.50731.

Muramyl dipeptide derivative 16

To compound **15** (0.2 g, 0.2 mmol) dissolved in DMF (4 mL, 0.05 M) was added AcOH (50 µL, 0.85 mmol), Bu_3SnH (0.1 mL, 0.4 mmol) and $Pd(PPH_3)_4$ (8 mg, 7 µmol). The resulting solution was stirred for 1.5 h. Crude compound **15** was precipitated by adding Et_2O. A second precipitation ($CHCl_3$/MeOH/Et_2O) yielded compound **21** (0.14 g, 0.13 mmol, 72%). R_f = 0.3 (8 : 2 $CHCl_3$: MeOH); $[\alpha]_D$ = -20.0 (c = 0.75, $CHCl_3$: MeOH); ^{1}H NMR (600 MHz, DMSO-D_6) δ 8.07 (d, J = 8.2 Hz, 1H, NH), 7.95 – 7.85 (m, 3H, CH, CHPh, NH), 7.78 (s, 1H, NH), 7.72 (d, J = 7.3 Hz, 2H, NH), 7.66 – 7.58 (m, 3H, CH, Ar), 7.58 – 7.52 (m, 1H, NH), 7.46 – 7.24 (m, 14H, CH, Ar), 7.07 (s, 1H, NH), 5.68 (s, 1H, CH, benzylidene acetal), 4.46 (d, J = 8.3 Hz, 1H, CH, H-1), 4.32 – 4.17 (m, 5H, CH_2, H-6, CH_2, $C_3H_6N_3$, CH_2, Fmoc, CH, Ala, CH, α Glu), 4.15 – 4.11 (m, 1H, CH, α i-D-Gln), 4.06 (q, J = 6.5 Hz, 1H, CH, lactic acid), 3.94 – 3.92 (m, 1H, CH, Fmoc), 3.82 – 3.69 (m, 3H, CH, H-2, CH_2, H-6, CH_2, $C_3H_6N_3$), 3.66 – 3.60 (m, 2H, CH, H-3, CH, H-4), 3.55 – 3.33 (m, 2H, CH, H-5, CH_2, $C_3H_6N_3$), 3.10 – 2.99 (m, 2H, CH_2, $C_3H_6N_3$), 2.21 – 2.15 (m, 4H, CH_2, γ i-D-Gln, CH_2, γ Glu), 2.03 – 1.86 (m, 2H, CH_2 β i-D-Gln, CH_2 β Glu), 1.92 – 1.72 (m, 4H, CH_3, NAc, CH_2, β Glu), 1.68 – 1.57 (m, 1H, CH_2, β i-D-Gln), 1.56 – 1.51 (m, 2H, CH_2 $C_3H_6N_3$), 1.36 (s, 9H, CH_3, ^{t}Bu), 1.24 (d, J = 6.9 Hz, 3H, CH_3, lactic acid), 1.20 (d, J = 6.6 Hz, 3H, CH_3, Ala); ^{13}C NMR (151 MHz, DMSO-D_6) δ 173.1 (C=O), 172.1 (C=O), 171.8 (C=O), 171.6 (C=O), 171.4 (C=O), 169.9 (C=O), 156.1 (C=O), 143.8 (C_q, Fmoc), 140.7 (C_q, Fmoc), 137.6 (C_q, benzylidene acetal), 132.1 (CH, Ar), 131.5 (CH, Ar), 131.5 (CH, Ar), 128.8 (CH, Ar), 128.8 (CH, Ar), 128.8 (CH, Ar), 128.1 (CH, Ar), 127.7 (CH, Ar), 127.1 (CH, Ar), 125.9 (CH, Ar), 125.3 (CH, Ar), 120.1 (CH, Ar), 101.5 (CH, C-1), 100.1 (CH, benzylidene acetal), 80.3 (CH, C-4), 79.7 (C_q, ^{t}Bu), 79.1 (CH, C-3), 77.3 (CH, lactic acid), 67.8 (CH_2, C-6), 66.9 (CH_2, $C_3H_6N_3$), 65.7 (CH_2, Fmoc), 65.6 (CH, C-5), 54.7 (CH, C-2), 53.6 (CH, Fmoc), 51.5 (CH, α i-D-Gln), 48.3 (CH, Ala), 46.7 (CH, α Glu), 35.5 (CH_2, $C_3H_6N_3$), 31.8 (CH_2, γ Glu), 31.2 (CH_2, γ i-D-Gln), 29.3 (CH_2, $C_3H_6N_3$), 27.7 (CH_3 ^{t}Bu), 27.2 (CH_2, β Glu, CH_2, β i-D-Gln), 23.0 (CH_3, NAc), 19.0 (CH_3, lactic acid), 18.2 (CH_3, Ala); IR (cm^{-1}): 3282, 1720, 1639, 1539, 1369; LC/MS: Rt = 8.85 min (Alltima C_{18}, 10 – 90% MeCN, 15 min run); HRMS Calcd. for $[C_{53}H_{68}N_6O_{16} + H]^+$ 1045.47646, found 1045.47762.

Muramyl dipeptide derivative 17

Compound **14** (0.21 g, 0.29 mmol) was suspended in 60% AcOH in H_2O (3.0 mL, 0.1 M) and stirred with neopentylglycol (60 mg, 0.58 mmol) at 65 °C for 3 h. The solution was diluted with H_2O, concentrated in vacuo and co-evaporated (toluene). Purification by flash chromatography (9:1 DCM/MeOH) resulted in compound **17** as a white solid (0.17 g, 0.26 mmol, 88%). R_f = 0.2 (9 : 1 $CHCl_3$: MeOH); $[\alpha]_D$ = 3.0 (c = 0.2, 1: 1 $CHCl_3$: MeOH);

^{1}H NMR (600 MHz, DMSO-D_6) δ 4.31 – 4.20 (m, 2H, CH, H-1, CH, lactic acid), 4.18 – 4.08 (m, 2H, CH, Ala, CH, α i-D-Gln), 3.78 – 3.75 (m, 1H, CH_2, $C_3H_6N_3$), 3.72 – 3.66 (m, 1H, CH_2, H-6), 3.60 (q, J = 9.2 Hz, 1H, CH, H-2), 3.54 – 3.43 (m, 2H, CH_2, H-6, CH_2. $C_3H_6N_3$), 3.41 – 3.26 (m, 3H, CH, H-3, CH_2, $C_3H_6N_3$), 3.26 – 3.21 (m, 1H, CH, H-4), 3.16 -1.13 (m, 1H, CH, H-5), 2.19 (t, 2H, J =5.2 Hz, CH_2, γ i-D-Gln), 1.97 – 1.91 (m, 1H, CH_2, β i-D-Gln), 1.78 (s, 3H, HNAc), 1.74 – 1.67 (m, 3H, CH_2, β i-D-Gln, CH_2, $C_3H_6N_3$), 1.39 (s, 9H, CH_3, ^{t}Bu), 1.26 – 1.24 (m, 6H, CH_3, lactic acid, CH_3, Ala); ^{13}C NMR (100 MHz, DMSO-D_6) δ = 173.1 (C=O), 172.3 (C=O), 172.1 (C=O), 171.6 (C=O), 169.3 (C=O), 100.8 (CH, C-1), 82.2 (CH, C-3), 76.8 (CH, C-5), 76.7 (CH_2, C-6), 69.3 (CH, C-4), 65.2 (CH_2, $C_3H_6N_3$), 60.8 (CH_2, C-6), 51.5 (CH, Ala), 48.2 (CH, α i-D-Gln), 47.5 (CH_2, $C_3H_6N_3$), 31.2 (CH_2, γ i-D-Gln), 28.5 (CH_2, $C_3H_6N_3$), 27.7 (CH_3, ^{t}Bu), 27.1 (CH_2, β i-D-Gln), 23.0 (CH_3, HNAc), 19.0 (CH_3, lactic acid), 18.1 (CH_3, Ala); IR (cm^{-1}): 3278, 2098, 1643, 1539, 1369; LC/MS: Rt = 4.52 min (C_{18} Alltima, 10 – 90% MeCN, 15 min run); HRMS Calcd. for $[C_{26}H_{45}N_7O_{11} + H]^+$ 632,32498, found 632,32516.

Muramyl dipeptide derivative 18

Compound **18** (0.21 g, 0.33 mmol) was co-evaporated with pyridine, dissolved in warm pyridine (0.92 mL, 11 mmol) and diluted with 1,4-dioxane (3 mL, 0.1 M). Ac_2O (0.27 mL, 2.9 mmol) was added and the solution was stirred for 48 h. The reaction mixture was quenched with MeOH and concentrated in vacuo and co-evaporated with toluene to yield crude compound **18**. R_f = 0.7 (9 : 1 $CHCl_3$: MeOH + 1% AcOH); $[\alpha]_D$ = 1.5 (c = 0.65, 1 : 1 $CHCl_3$: MeOH); ^{1}H NMR (400 MHz, MeOD) δ 4.92 (t, J = 9.4 Hz, 1H, CH, H-4), 4.45 (d, J = 8.1 Hz, 1H, CH, H-1), 4.30 (dd, J = 9.3, 4.6 Hz, 1H, CH, α i-D-Gln), 4.20 (dd, J = 12.2, 4.8 Hz, 1H, CH_2, H-6), 4.16 – 4.04 (m, 2H, CH_2, H-6, CH, lactic acid), 3.98 (q, J = 6.7 Hz, 1H, CH, Ala), 3.92 – 3.83 (m, 1H, CH_2, $C_3H_6N_3$), 3.78 -3.71 (m, 2H, CH, H-2, CH, H-3), 3.63 -3.59 (m,

1H, CH, H-5), 3.56 – 3.52 (m, 1H, CH_2, $C_3H_6N_3$), 3.33 (t, J = 6.6 Hz, 2H, CH_2, $C_3H_6N_3$), 2.30 – 2.25 (m, 2H, CH_2, γ i-D-Gln), 2.22 – 2.09 (m, 1H, CH_2, β i-D-Gln), 2.07 (s, 3H,CH_3, Ac), 2.05 (s, 3H, CH_3, Ac), 1.88 (s, 3H, CH_3, NAc), 1.85-1.75 (m, 3H, CH_2, $C_3H_6N_3$, CH_2, β i-D-Gln), 1.50 – 1.537 (m, J = 6.2 Hz, 12H, CH_3, ^{t}Bu, CH_3, lactic acid), 1.23 (d, J = 6.8 Hz, 3H, CH_3, Ala);

^{13}C NMR (100 MHz, MeOD) δ 174.2 (C=O), 173.5 (C=O), 172.8 (C=O), 172.3 (C=O), 171.6 (C=O), 170.9 (C=O), 169.8 (C=O), 100.5 (CH, C-1), 80.6 (C_q, ^{t}Bu), 79.3 (CH, C-3), 77.7 (CH, lactic acid), 71.2 (CH, C-5), 69.2 (CH, C-4), 65.9 (CH_2, $C_3H_6N_3$), 62.0 (CH_2, C-6), 55.5 (CH, C-2), 51.8 (CH, α i-D-Gln), 49.3 (CH, Ala), 47.5 (CH_2, $C_3H_6N_3$), 31.3 (CH_2, γ i-D-Gln), 28.5 (CH_2, $C_3H_6N_3$), 27.3 (CH_3, ^{t}Bu), 26.4 (CH_2, β i-D-Gln), 22.9 (CH_3, NAc), 20.9 (CH_3, Ac), 20.8 (CH_3, Ac), 19.1 (CH_3, lactic acid), 17.3 (CH_3, Ala); IR (cm^{-1}): 3275, 2098, 1639, 1091, 694; LC/MS: Rt = 6.42 min (Alltima C_{18}, 10 –90% MeCN, 15 min run); HRMS Calcd. for $[C_{30}H_{49}N_7O_{13} + H]^+$ 716.34611 found 716.34653.

Muramyl dipeptide derivative 19

Compound **18** (73 mg, 0.11 mmol) was dissolved in a mixture of 20% TFA in DCM (1 mL, 0.1 M) and stirred for 2.5 h at ambient temperature. The compound was precipitated using Et_2O. The resulting solid was purified by flash column chromatography (9:1 $CHCl_3$/MeOH + 1% AcOH) yielding compound **19** (54 mg, 80 µmol, 82%). R_f = 0.2 (9 : 1 $CHCl_3$: MeOH + 1% AcOH); $[\alpha]_D$ = -5.2 (c = 0.27, 1 : 1 $CHCl_3$: MeOH); ^{1}H NMR (400 MHz, MeOD) δ 4.96 (t, J = 9.6 Hz, 1H, CH, H-4), 4.46 (d, J = 8.4 Hz, 1H, CH, H-1), 4.37 – 4.35 (m, 1H, CH, α i-D-Gln), 4.26 (dd, J = 12.3, 4.6 Hz, 1H, CH_2, H-6), 4.19 (q, J = 7.1 Hz, 1H, CH, lactic acid), 4.11 (dd, J = 12.3, 2.1 Hz, 1H, CH_2, H-6), 4.08 – 4.03 (m, 1H, CH, Ala), 3.96 – 3.86 (m, 2H, CH_2, $C_3H_3N_3$, CH, H-2), 3.77 – 3.67 (m, 2H, CH, H-3, CH, H-5), 3.70 – 3.59 (m, 1H, CH_2, $C_3H_3N_3$), 3.41 – 3.33 (m, 2H, CH_2, $C_3H_3N_3$), 2.38 (t, J = 7.6 Hz, 2H, CH_2, γ i-D-Gln), 2.30 – 2.19 (m 1H, CH_2, β i-D-Gln), 2.12 (s, 3H, Ac), 2.07 (s, 3H, CH_3, Ac), 1.97 – 1.88 (m, 4H, CH_3, NAc, CH_2, β i-D-Gln), 1.88 – 1.72 (m, 2H, CH_2, $C_3H_3N_3$), 1.41 (d, J = 7.1 Hz, 3H, CH_3, lactic acid), 1.27 (d, J = 6.7 Hz, 3H, CH_3, Ala); ^{13}C NMR (100 MHz, MeOD) δ 176.4 (C=O), 175.9 (C=O), 175.0 (C=O), 174.7 (C=O), 173.4 (C=O), 172.6 (C=O), 171.7 (C=O), 102.0 (CH, C-1), 80.8 (CH, C-3), 79.1 (CH, lactic acid), 72.4 (CH, C-5), 70.5 (CH, C-4), 67.3 (CH_2, $C_3H_3N_3$), 63.2 (CH_2, C-6), 56.5 (CH, C-2), 53.3 (CH, α i-D-Gln), 50.6 (CH, Ala), 48.4 (CH_2, $C_3H_3N_3$), 31.0 (CH_2, γ i-D-Gln),

29.7 (CH_2, $C_3H_3N_3$), 27.6 (CH_2, β i-D-Gln), 23.1 (CH_3, NAc), 21.1 (CH_3, Ac), 20.9 (CH_3, Ac), 19.4 (CH_3, lactic acid), 17.5 (CH_3, Ala); IR (cm^{-1}): 3294, 2098, 1654, 1535; LC/MS: Rt = 4.85 min (Alltima C_{18}, 10 – 90% MeCN, 15 min run); HRMS Calcd. for $[C_{26}H_{41}N_7O_{13} + H]^+$, 660.28351 found 660.28379.

Azidopropyl muramyl dipeptide 20

Compound **19** (15 mg, 23 µmol) was dissolved in a solution of 7 M ammonia in MeOH (1.5 mL). The solution was stirred for 5 h at ambient temperature. The reaction mixture was concentrated and purified over HW40 gel filtration chromatography (0.15 M, ammonium acetate). After lyophilization compound **25** was obtained as a white solid (12 mg, 20 µmol, 87%). R_f = 0.2 (8 : 2 $CHCl_3$: MeOH + 2% AcOH); $[\alpha]_D$ = -12.5 (c = 0.02, 1 : 1 $CHCl_3$: MeOH); ^1H NMR (400 MHz, D_2O) δ 4.56 (d, J = 8.5 Hz, 1H, CH, H-1), 4.47 – 4.43 (m, 1H, CH, α i-D-Gln), 4.38 – 4.26 (m, 2H, CH, lactic acid, CH, Ala), 4.09 – 4.03 (m, 1H, CH_2, $C_3H_6N_3$), 4.01 (d, J = 1.9 Hz, 1H, CH_2, H-6), 3.93 – 3.87 (m, 1H, CH, H-2), 3.84 (d, J = 5.5 Hz, 1H, CH_2, H-6), 3.82 – 3.72 (m, 1H, CH_2, $C_3H_6N_3$), 3.65 – 3.52 (m, 3H, H-3, CH, H-4, CH, H-5), 3.31 – 3.27 (m, 2H, CH_2, $C_3H_6N_3$), 2.50 (t, J = 7.3 Hz, 2H, CH_2, γ i-D-Gln), 2.33 – 2.20 (m, 2H, CH_2, β i-D-Gln), 2.11 – 2.01 (m, 4H, CH_2, β i-D-Gln, 3H, CH_3, NAc), 1.81 -1.72 (m, 2H, CH_2, $C_3H_6N_3$), 1.53 (d, J = 7.2 Hz, 3H, CH_3, lactic acid), 1.46 (d, J = 6.8 Hz, 3H, CH_3, Ala). ^{13}C NMR (151 MHz, D_2O) δ 179.5 (C=O), 177.1 (C=O), 176.7 (C=O), 176.1 (C=O), 175.0 (C=O), 102.2 (CH, C-1), 83.7 (CH, C-3), 79.3 (CH, lactic acid), 76.5 (CH, C-5), 69.8 (CH, C-4), 68.1 (CH_2, $C_3H_6N_3$), 61.6 (CH_2, C-6), 56.0 (CH, C-2), 53.9 (CH, α i-D-Gln), 50.8 (CH, Ala), 49.0 (CH_2, $C_3H_6N_3$), 32.4 (CH_2, γ i-D-Gln), 29.1 (CH_2, β i-D-Gln), 27.6 (CH_2, $C_3H_6N_3$), 23.2 (CH_3, NAc), 19.7 (CH_3 lactic acid), 17.5 (CH_3, Ala); IR (cm^{-1}): 3310, 2101, 1645, 1464; LC/MS: Rt = 8.07 min (Alltima C_{18}, 0 – 20% MeCN, 15 min run); HRMS Calcd. for $[C_{22}H_{37}N_7O_{11} + H]^+$, 576.26238 found 576.26243.

Automated synthesis of MDP-peptide conjugates

General procedure for automated solid-phase synthesis

The solid-phase peptide synthesis was performed on 50 µmol or 25 µmol scale according to established methods [1] on an ABI 433A (Applied Biosystems) automated instrument applying an Fmoc based protocol starting from Tentagel-S-

RAM resin (loading 0.23 mmol/g). The consecutive steps performed in each cycle for HCTU chemistry on 50 µmol scale:

1. Deprotection of the Fmoc-group with 20% piperidine in NMP for 15 min; 2) NMP wash; 3) Coupling of the appropriate amino acid using a five-fold excess. Generally, the Fmoc amino acid (0.25 mmol) was dissolved in 0.25 M HCTU in NMP (1 mL), the resulting solution was transferred to the reaction vessel followed by 0.5 mL of 1.0 M DiPEA in NMP to initiate the coupling. The reaction vessel was shaken for 30 min; 4) NMP wash; 5) capping with 0.5 M acetic anhydride in NMP in presence of 0.5 mmol DiPEA; 6) NMP wash; 7) DCM wash.

The consecutive steps performed in each cycle for HATU chemistry:

1. Deprotection of the Fmoc-group with 20% piperidine in NMP for 15 min; 2) NMP wash; 3) Coupling of the appropriate amino acid using a two-fold or five-fold excess. Generally, the Fmoc amino acid (0.1 mmol or 0.25 mmol) and HATU (0.15 mmol or 0.2 mmol) was dissolved in 1.0 M DiPEA in NMP (0.25 mL or 0.5 mL). The resulting solution was pre-activated for 1 min and transferred to the reaction vessel to initiate the coupling. The reaction vessel was shaken for 60 min; 4) NMP wash; 5) capping with 0.5 M acetic anhydride in NMP in presence of 0.5 mmol DiPEA; 6) NMP wash; 7) DCM wash.

Aliquots of resin of the obtained sequences were checked by HPLC using an analytical Alltima C_{18} column (4.6 × 50 mm, 5 µm particle size, flow 1.0 mL/min.). The Fmoc amino acids applied in the synthesis were: Fmoc-Ala-OH, Fmoc-Asn(Trt)-OH, Fmoc-Asp(O*t*-Bu)-OH, Fmoc-Arg(Pbf)-OH, Fmoc-Gln(Trt)-OH, Fmoc-Glu(O*t*-Bu)-OH, Fmoc-Gly-OH, Fmoc-Glu(O*t*-Bu)-OH, Fmoc-Ile-OH, Fmoc-Leu-OH, Fmoc-Lys(Boc)-OH, Fmoc-Lys(MMt)-OH, Fmoc-Phe-OH, Fmoc-Ser(*t*-Bu)-OH and Fmoc-Val-OH.

General procedure for cleavage from the resin, deprotection and purification

The resin was washed with NMP, DCM and dried after the last synthesis step. Next it was treated with 5 mL cleavage cocktail (95% TFA, 2.5% TIS and 2.5% H_2O) for 104 min. The suspension was filtered, the resin was washed with neat TFA and the product was precipitated with Et_2O out of the TFA solution. The suspension of the

product in Et_2O was centrifuged, Et_2O removed and the precipitate was washed with Et_2O (3×). The final precipitate was air dried and dissolved in AcOH/H_2O (1:1) or MeCN/H_2O/*t*-BuOH (1:1:1) followed by RP-HPLC purification.

3-Azidopropyl-MurNAc-Ala-D-isoGln-Asp-Glu-Val-Ser-Gly-Leu-Glu-Gln-Leu-Glu-Ser-Ile-Ile-Asn-Phe-Glu-Lys-Leu-Ala-Ala-Ala-Ala-Ala-Lys-NH_2 (2)

Tentagel S Ram resin loaded with H-Asp(O*t*-Bu)-Glu(O*t*-Bu)-Val-Ser(*t*-Bu)-Gly-Leu-Glu(O*t*-Bu)-Gln(Trt)-Leu-Glu(O*t*-Bu)-Ser(*t*-Bu)-Ile-Ile-Asn(Trt)-Phe-Glu-Lys(Boc)-Leu-Ala-Ala-Ala-Ala-Ala-Lys(Boc) (25 µmol) was elongated with Fmoc-D-isoGln-OH and Fmoc-Ala-OH using a standard HCTU/Fmoc cycle. The synthesis was completed with a double coupling using two-fold excess of compound **10**, pre-activated with HATU and DiPEA. After treatment with the standard cleavage cocktail for 60 min. the suspension was filtered and the product was precipitated with Et_2O. After purification by RP-HPLC, compound **2** and hydrolyzed compound **22** were isolated. Compound **2** was obtained in 1.3 mg (0.41 µmol, 2%); LC/MS: Rt = 6.22 min (C_{18} Alltima, 10 - 90% MeCN, 15 min run); ESI-MS: m/z 3103.60 $[M+H]^+$; HRMS Calcd. for $[C_{134}H_{220}N_{36}O_{48} + H]^{2+}$ 1552.30276, found 1552.30469; Compound **22** was isolated in 1.5 mg (0.38 µmol, 2%); LC/MS: Rt = 6.05 min (C_{18} Alltima, 10 - 90% MeCN, 15 min run); ESI-MS: m/z 3020.55 $[M+H]^+$; HRMS Calcd. for $[C_{131}H_{215}N_{33}O_{48} + H]^{2+}$ 1510.77860, found 1510.78070

H-Asp-Glu-Val-Ser-Gly-Leu-Glu-Gln-Leu-Glu-Ser-Ile-Ile-Asn-Phe-Glu-Lys-Leu-Ala-Ala-Ala-Ala-Ala-Lys-(D-isoGln-Ala-3-azidopropyl-MurNAc)NH_2 (3)

Tentagel S Ram resin loaded with H-Asp(O*t*-Bu)-Glu(O*t*-Bu)-Val-Ser(*t*-Bu)-Gly-Leu-Glu(O*t*-Bu)-Gln(Trt)-Leu-Glu(O*t*-Bu)-Ser(*t*-Bu)-Ile-Ile-Asn(Trt)-Phe-Glu-Lys(Boc)-Leu-Ala-Ala-Ala-Ala-Ala-Lys(Boc) (25 µmol) was treated 1 M Boc_2O in NMP for 15 min followed by addition of 2 equiv of DiPEA. After one hour the resin was washed with NMP and DCM. The resin was treated with a cleavage cocktail of 3% TFA in DCM followed by a coupling sequence of Fmoc-D-isoGln-OH and Fmoc-Ala-OH with a standard HCTU/Fmoc cycle. The synthesis was completed with a double coupling of a two-fold excess of compound **10** pre-activated with HATU and DiPEA. After treatment with the standard cleavage cocktail for 60 min. the suspension was filtered and the product was precipitated using Et_2O. After HPLC purification, both compound **3** and hydrolyzed compound **25** were isolated.

Compound **3** was obtained in 1.2 mg (0.34 µmol, 2%); LC/MS: Rt = 6.16 min (C_{18} Alltima, 10 - 90% MeCN, 15 min run); ESI-MS: m/z 3103.60 $[M+H]^+$; HRMS Calcd. for $[C_{134}H_{220}N_{36}O_4 + H]^{2+}$ 1552.30276, found 1552.30434. Compound **25** was obtained in 1.0 mg (0.39 µmol, 2%); LC/MS: Rt = 6.02 min (C_{18} Alltima, 10 - 90% MeCN, 15 min run); ESI-MS: m/z 3020.55 $[M+H]^+$; HRMS Calcd. for $[C_{131}H_{215}N_{33}O_{48} + H]^{2+}$ 1510.77860, found 1510.78070.

H-Glu(3-azidopropyl-MurNAc-Ala-D-isoGln)-Asp-Glu-Val-Ser-Gly-Leu-Glu-Gln-Leu-Glu-Ser-Ile-Ile-Asn-Phe-Glu-Lys-Leu-Ala-Ala-Ala-Ala-Ala-Lys-NH_2 (4)

Resin **21** was treated with a mixture of compound **16** (53 mg, 50 µmol), HCTU (21 mg, 50 µmol) and DiPEA (18 µL, 0.1 mmol) in NMP (0.5 mL) for 18 h. The resin was washed and treated with a solution of 20% piperidine in NMP followed by a wash step (NMP, DCM, Et_2O). Treatment with the cleavage cocktail for 60 min and purification resulted in compound **4** (1.8 mg, 0.53 µmol, 2%), LC/MS: Rt = 8.78 min (C_{18} Alltima, 10 - 50% MeCN, 15 min run); ESI-MS: m/z 3206.65 $[M+H]^+$; HRMS Calcd. for $[C_{139}H_{229}N_{35}O_{51} + H]^{2+}$ 1603.82882, found 1603.82944.

H-Asp-Glu-Val-Ser-Gly-Leu-Glu-Gln-Leu-Glu-Ser-Ile-Ile-Asn-Phe-Glu-Lys-Leu-Ala-Ala-Ala-Ala-Ala-Lys-D-isoGln(3-azidopropyl-MurNAc-Ala-D-isoGln)NH_2 (5)

40 µmol Sieber Amide resin (0.2 mmol/g) was treated with 20% piperidine in NMP (3 × 3 min), washed with NMP and treated with a mixture of compound **16** (107 mg, 102 µmol), DiPEA (67 µL, 406 µmol) and HCTU (76 mg, 184 µmol) in 4:1 NMP/DMSO. The resulting suspension was shaken for 18 h, washed with NMP, DCM and Et_2O. An aliquot of resin (5 mg) was treated with 20% piperidine (1 mL) for 20 min. The solution was diluted with EtOH (25 mL) and absorbance at 300 nm was measured. The Fmoc-test revealed a 56% loading. The resin was treated with a capping solution (0.5 M Ac_2O, 0.05 M DiPEA, NMP, 3 × 15 min) and elongated with the standard Fmoc based SPPS protocol to H-Asp(O*t*-Bu)-Glu(O*t*-Bu)-Val-Ser(*t*-Bu)-Gly-Leu-Glu(O*t*-Bu)-Gln(Trt)-Leu-Glu(O*t*-Bu)-Ser(*t*-Bu)-Ile-Ile-Asn(Trt)-Phe-Glu-Lys(Boc)-Leu-Ala-Ala-Ala-Ala-Ala-Lys(Boc) concluding with a final Fmoc deprotection. 20 µmol resin was treated with standard cleavage conditions. After

purification compound **5** and hydrolyzed analogue **28** were isolated. Compound **5** was obtained in 4.8 mg (1.4 μmol, 6%). LC/MS: Rt = 5.70 min (C_{18} Alltima, 10 - 90% MECN, 15 min run); ESI-MS: m/z 3206.65 $[M+H]^+$; HRMS Calcd. for $[C_{139}H_{229}N_{35}O_{51} + H]^{2+}$ 1603.82882, found 1603.82943. Compound **28** was isolated in 2.8 mg (0.94 μmol, 4%); LC/MS: Rt = 5.82 min (C_{18} Alltima, 10 - 90% MeCN, 15 min run); ESI-MS: m/z 2731.45 $[M+H]^+$; HRMS Calcd. for $[C_{120}H_{198}N_{31}O_{41} + H]^{3+}$ 911.15363, found 911.15694.

Immunological assays

Cell culture

The D1 cell line is a growth factor-dependent immature spleen-derived DC cell line from C57BL/6 (H-2_b) mice. D1 cells were cultured as described [2]. The B3Z hybridoma is cultured in complete IMDM medium supplemented with 500 μg/mL hygromycin [3]. NOD2-HEK293 cells were cultured in complete IMDM medium, supplemented with 10 μg/ml blasticidin.

NOD2-HEK293 activation

The human NOD2-receptor expressing the HEK293 cell-line was obtained from Invivogen (Toulouse, France). Test compounds were titrated in a 96-wells plate and approximately 50.000 NOD2-HEK293 cells were subsequently added per well. After 24 hours of incubation at 37 °C, the supernatant was taken from all wells. The amount of IL-8 produced by the NOD2-HEK293 cells is a measure for NOD2-mediated activation. The concentration of IL-8 in the supernatant was determined using an IL-8 ELISA-kit (Sanquin, Amsterdam, The Netherlands).

In vitro DC maturation assay

Test compounds were titrated in a 96-wells plate (Corning, Amsterdam, The Netherlands) in complete IMDM medium. Next, D1 cells from C57BL/6 mice were harvested and counted, and subsequently transferred to the 96-wells plates containing the test compound titrations, using approximately 40.000 cells per well. After 24 hours of incubation at 37 °C, supernatant was taken from the wells for ELISA analysis (BioLegend, San Diego, USA) in which the amount of produced IL-12p40 was measured.

In vitro antigen presentation assay

B3Z is a $CD8^+$ T-cell hybridoma specific for the H-$2K^b$ CTL-epitope SIINFEKL of ovalbumin. B3Z expresses the lacZ reporter gene of Escherichia coli, which is under the regulation of the NFAT element from the IL-2 promoter. Therefore, TCR triggering of this T-cell leads to transcription of the lacZ reporter gene, the gene product of which is able to convert the chromogenic substrate CPRG (Chlorophenolred-β-D-galactopyranoside). This conversion is measured by absorbance spectrophotometry at a wavelength of 590 nm [3].

References

(1) Chan, W. C.; White, P. D. *Fmoc solid phase peptide synthesis*; Oxford University Press Inc.: New York, 2000.

(2) Winzler, C.; Rovere, P.; Zimmermann, V. S.; Davoust, J.; Rescigno, M.; Citterio, S.; Ricciardi-Castagnoli, P. *Adv. Exp. Med. Biol.* **1997**, *417*, 59-64.

(3) Sanderson, S.; Shastri, N. *Int. Immunol.* **1994**, *6*, 369-376.
