# Supplementary material for: Design, automated synthesis and immunological evaluation of NOD2-ligand–antigen conjugates
Source: Beilstein J Org Chem. 2014 Jun 26;10:1445–53. doi: 10.3762/bjoc.10.148 (PMC4077378; doi:10.3762/bjoc.10.148)
Supplement: File 2 — NMR spectra. [file Beilstein_J_Org_Chem-10-1445-s002.pdf]

Supporting Information File 2

for

Design, automated synthesis and immunological evaluation of NOD2-ligand–antigen conjugates

Marian M. J. H. P. Willems[1], Gijs G. Zom[2], Nico Meeuwenoord[1], Ferry A. Ossendorp[2], Herman S. Overkleeft[1], Gijsbert A. van der Marel[1], Jeroen D. C. Codée[1*] and Dmitri V. Filippov[1*]

Address: [1]Leiden Institute of Chemistry, Leiden University, P.O. Box 9502, 2300 RA Leiden, The Netherlands and [2]Department of Immunohematology and Blood Transfusion, Leiden University Medical Centre, P. O. Box 9600, 2300 RC Leiden, The Netherlands

Email: Jeroen D. C. Codée - jcodee@chem.leidenuniv.nl; Dmitri V. Filippov - filippov@chem.leidenuniv.nl

*Corresponding author

NMR spectra

Contents:

Compound	**8**	^{1}H NMR 400 MHz CD_3CD	p S2-S3
Compound	**8**	^{13}C NMR 100 MHz CD_3CD	p S4
Compound	**9**	^{1}H NMR 400 MHz DMSO-d_6	p S5
Compound	**9**	^{13}C NMR 100 MHz DMSO-d_6	p S6
Compound	**10**	^{1}H NMR 400 MHz CD_3CD	p S7
Compound	**10**	^{13}C NMR 100 MHz CD_3CD	p S8
Compound	**12**	^{1}H NMR 400 MHz DMSO-d_6	p S9
Compound	**12**	^{13}C NMR 100 MHz DMSO-d_6	p S10
Compound	**13**	^{1}H NMR 400 MHz DMSO-d_6	p S11
Compound	**13**	^{13}C NMR 100 MHz DMSO-d_6	p S12
Compound	**14**	^{1}H NMR 400 MHz CD_3CD	p S13-S14
Compound	**14**	^{13}C NMR 100 MHz CD_3CD	p S15
Compound	**15**	^{1}H NMR 400 MHz DMSO-d_6	p S16
Compound	**15**	^{13}C NMR 100 MHz DMSO-d_6	p S17
Compound	**16**	^{1}H NMR 600 MHz DMSO-d_6	p S18
Compound	**16**	^{13}C NMR 150 MHz DMSO-d_6	p S19
Compound	**17**	^{1}H NMR 600 MHz DMSO-d_6	p S20
Compound	**17**	^{13}C NMR 150 MHz DMSO-d_6	p S21
Compound	**17**	^{1}H NMR 400 MHz D_2O	p S22-S23
Compound	**18 (crude)**	^{1}H NMR 400 MHz CD_3CD	p S24
Compound	**18 (crude)**	^{13}C NMR 100 MHz CD_3CD	p S25
Compound	**19**	^{1}H NMR 400 MHz CD_3CD	p S26
Compound	**19**	^{13}C NMR 100 MHz CD_3CD	p S27
Compound	**20**	^{1}H NMR 400 MHz D_2O	p S28
Compound	**20**	^{13}C NMR 100 MHz CD_3CD	p S29

S2

1h NMR av400liq
mw034

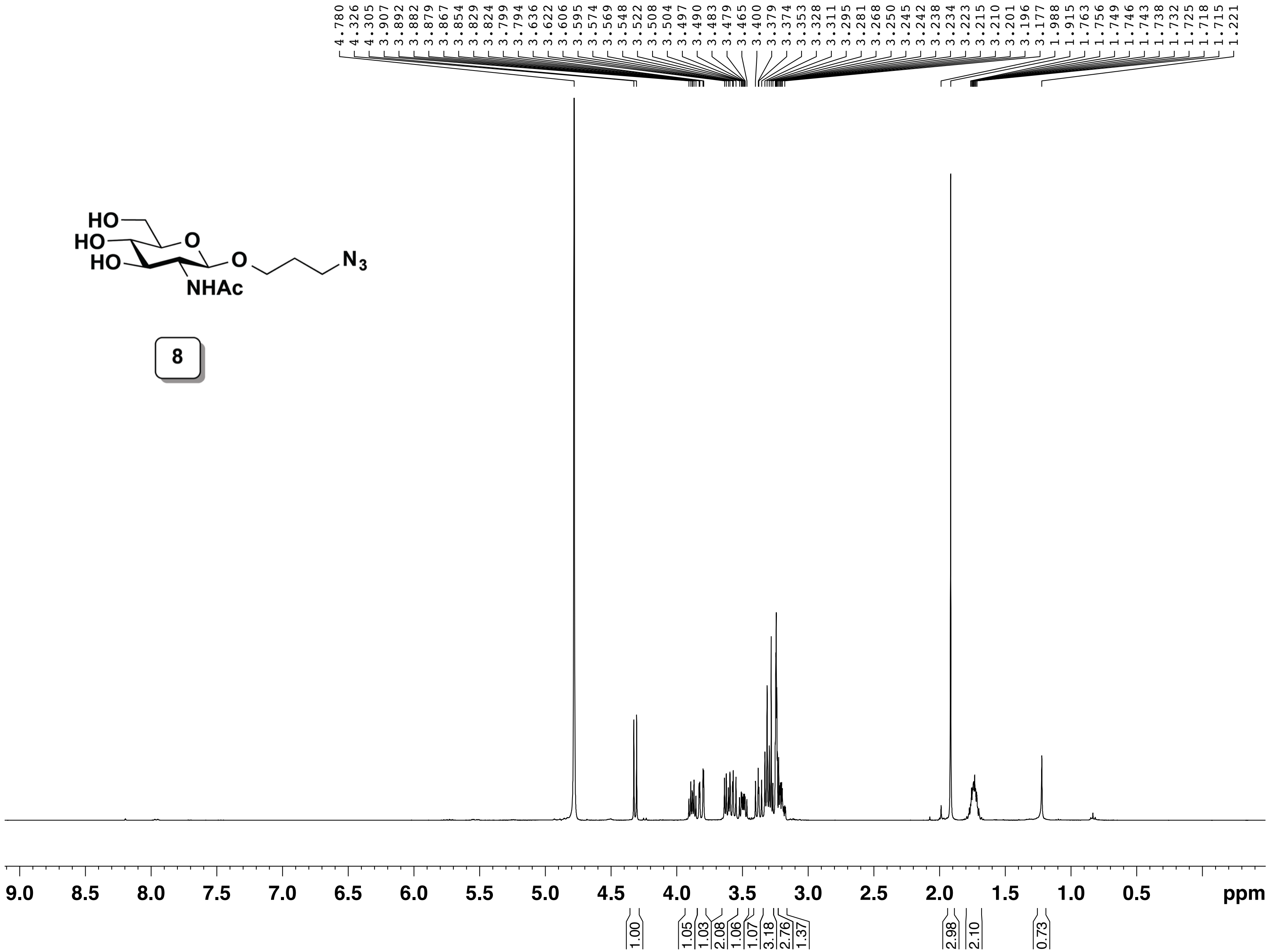

NAME	0807marian
EXPNO	57
PROCNO	1
Date_	20080716
Time	9.58
INSTRUM	spect
PROBHD	5 mm PABBO BB-
PULPROG	zg30
TD	65536
SOLVENT	MeOD
NS	16
DS	0
SWH	8223.685 Hz
FIDRES	0.125483 Hz
AQ	3.9846387 sec
RG	203
DW	60.800 use
DE	6.50 use
TE	298.0 K
D1	1.00000000 sec
TD0	1

======== CHANNEL f1 =======

NUC1	1H
P1	14.10 use
PL1	0.00 dB
PL1W	8.41481972 W
SFO1	400.2324716 MHz
SI	65536
SF	400.2300373 MHz
WDW	EM
SSB	0
LB	0.30 Hz
GB	0
PC	1.00

S3

1h NMR av400liq
mw034

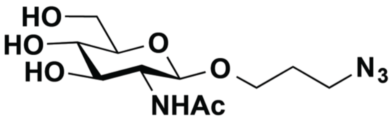

8

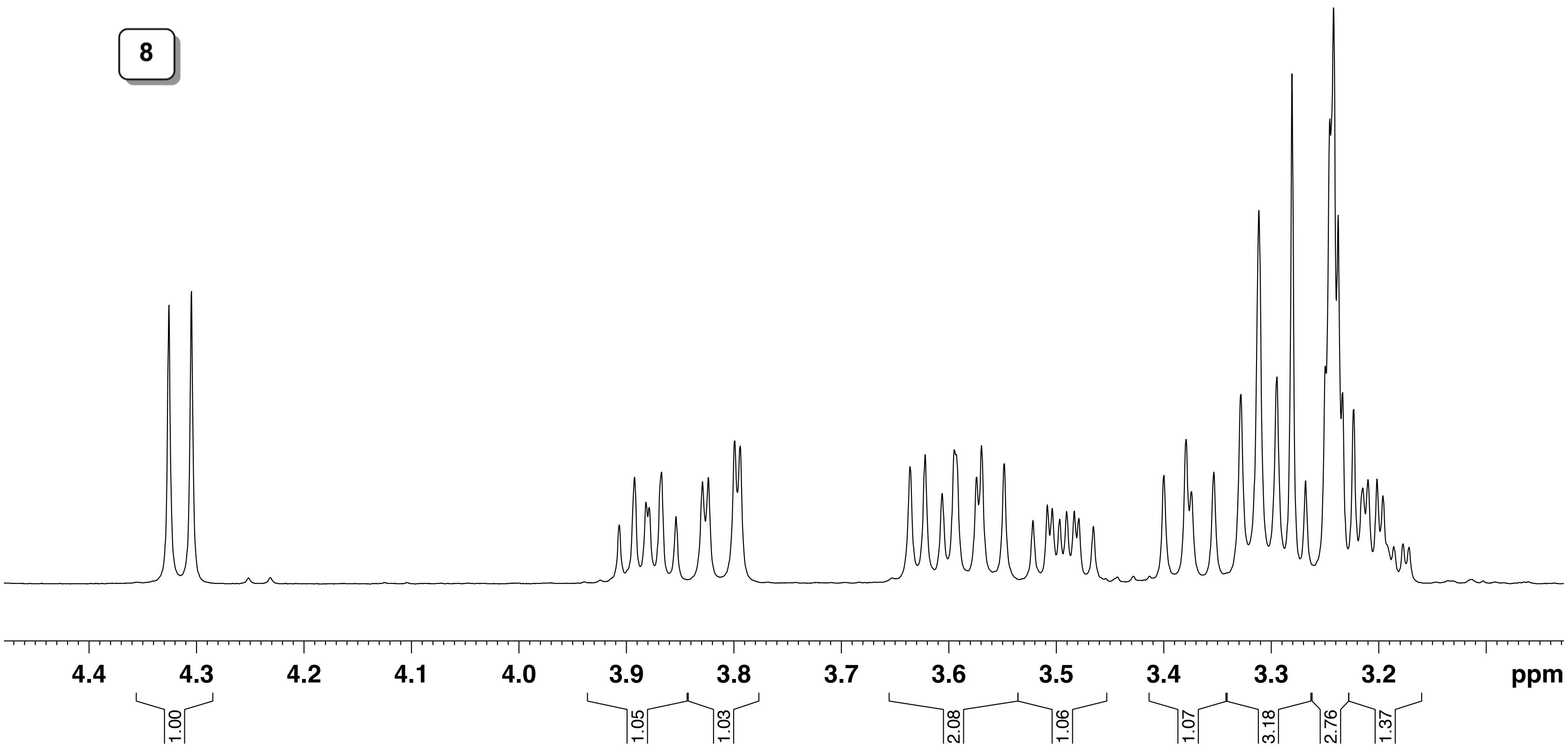

NAME	0807marian
EXPNO	57
PROCNO	1
Date_	20080716
Time	9.58
INSTRUM	spect
PROBHD	5 mm PABBO BB-
PULPROG	zg30
TD	65536
SOLVENT	MeOD
NS	16
DS	0
SWH	8223.685 Hz
FIDRES	0.125483 Hz
AQ	3.9846387 sec
RG	203
DW	60.800 use
DE	6.50 use
TE	298.0 K
D1	1.00000000 sec
TD0	1

======== CHANNEL f1 ========

NUC1	1H
P1	14.10 use
PL1	0.00 dB
PL1W	8.41481972 W
SFO1	400.2324716 MHz
SI	65536
SF	400.2300373 MHz
WDW	EM
SSB	0
LB	0.30 Hz
GB	0
PC	1.00

S4

mw034 13CNMR

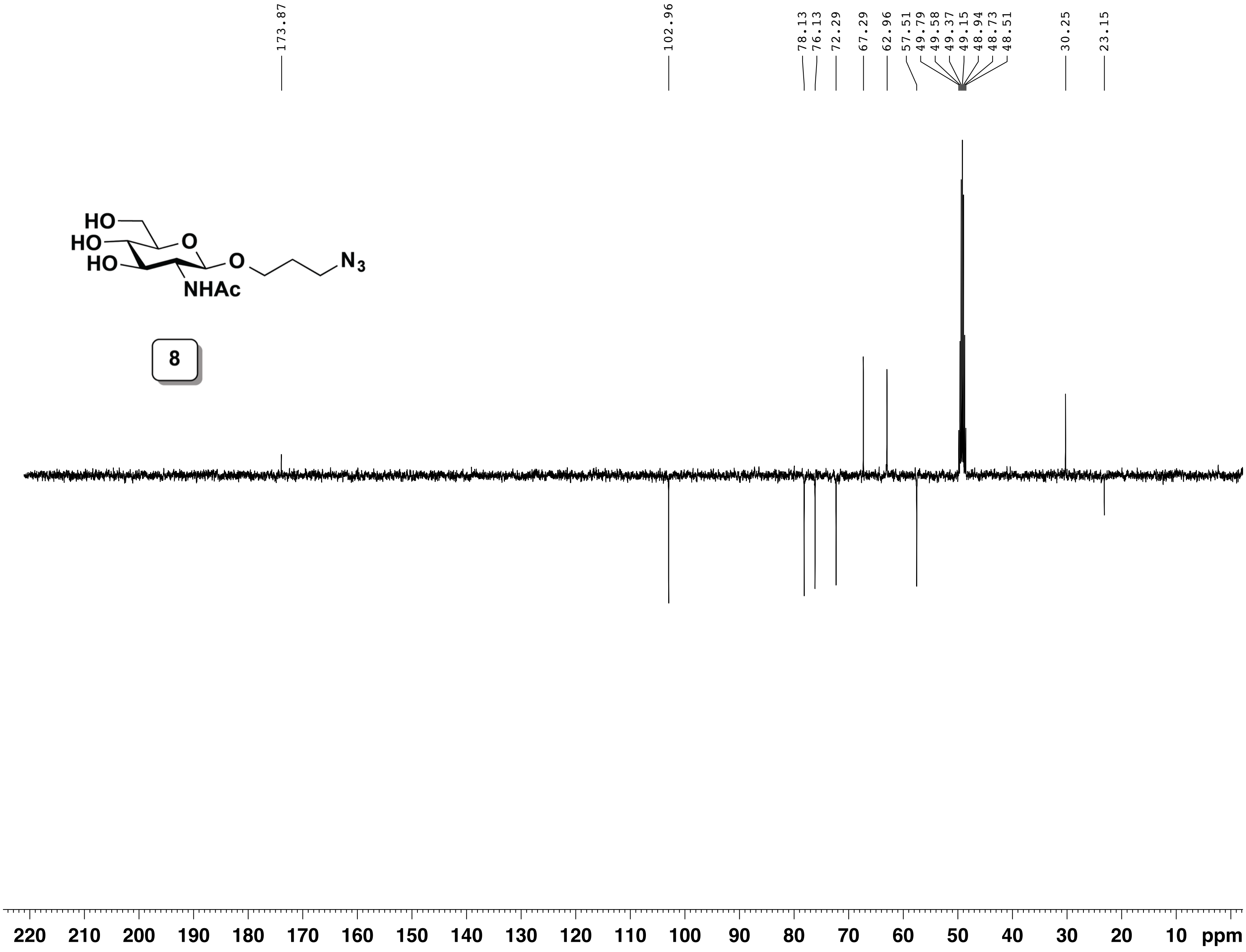

NAME	0807marian
EXPNO	58
PROCNO	1
Date_	20080716
Time	10.05
INSTRUM	spect
PROBHD	5 mm PABBO BB-
PULPROG	jmod
TD	65536
SOLVENT	MeOD
NS	221
DS	1
SWH	24038.461 H
FIDRES	0.366798 H
AQ	1.3631988 s
RG	2050
DW	20.800 u
DE	6.00 u
TE	298.9 K
CNST2	155.0000000
CNST11	1.0000000
D1	1.50000000 s
D20	0.00645161 s
TD0	1

======== CHANNEL f1 =====

NUC1	13C
P1	9.10 u
P2	18.20 u
PL1	-1.00 d
PL1W	44.27188873 W
SFO1	100.6479773 M

======== CHANNEL f2 =====

CPDPRG2	waltz16
NUC2	1H
PCPD2	80.00 u
PL2	0.00 d
PL12	15.00 d
PL2W	8.41481972 W
PL12W	0.26609996 W
SFO2	400.2316009 M
SI	32768
SF	100.6377580 M
WDW	EM
SSB	0
LB	2.50 H
GB	0
PC	1.40

S5

1h NMR av400liq mw173

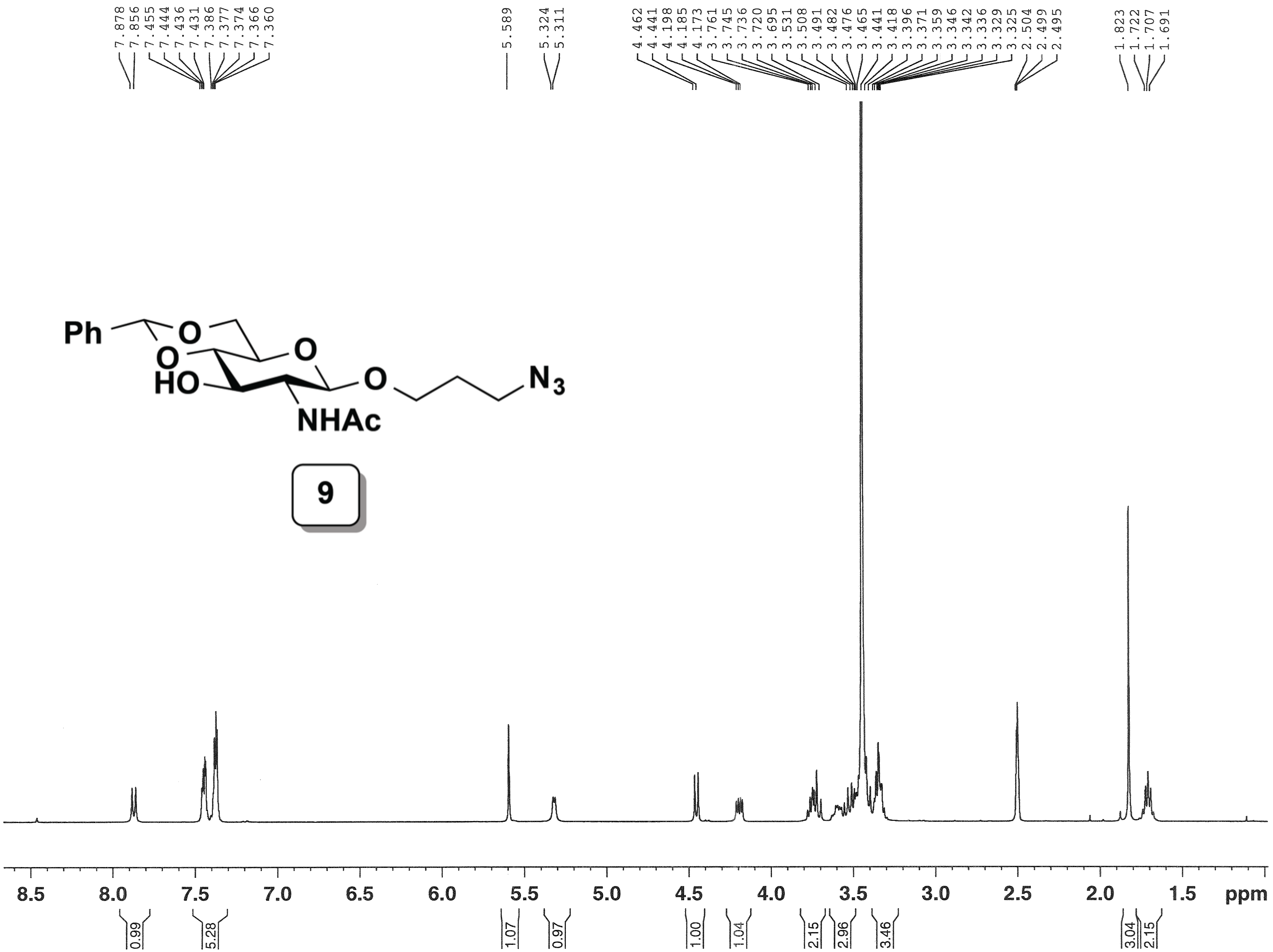

NAME	0906Marian
EXPNO	14
PROCNO	1
Date_	20090612
Time	9.54
INSTRUM	spect
PROBHD	5 mm PABBO BB-
PULPROG	zg30
TD	65536
SOLVENT	DMSO
NS	8
DS	0
SWH	8223.685 Hz
FIDRES	0.125483 Hz
AQ	3.9846387 sec
RG	114
DW	60.800 usec
DE	6.50 usec
TE	295.9 K
D1	1.00000000 sec
TD0	1

======== CHANNEL f1 ========

NUC1	1H
P1	14.10 usec
PL1	0.00 dB
PL1W	8.41481972 W
SFO1	400.2324716 MHz
SI	65536
SF	400.2300059 MHz
WDW	EM
SSB	0
LB	0.30 Hz
GB	0
PC	1.00

9

S6

13C APT av400liq mw173

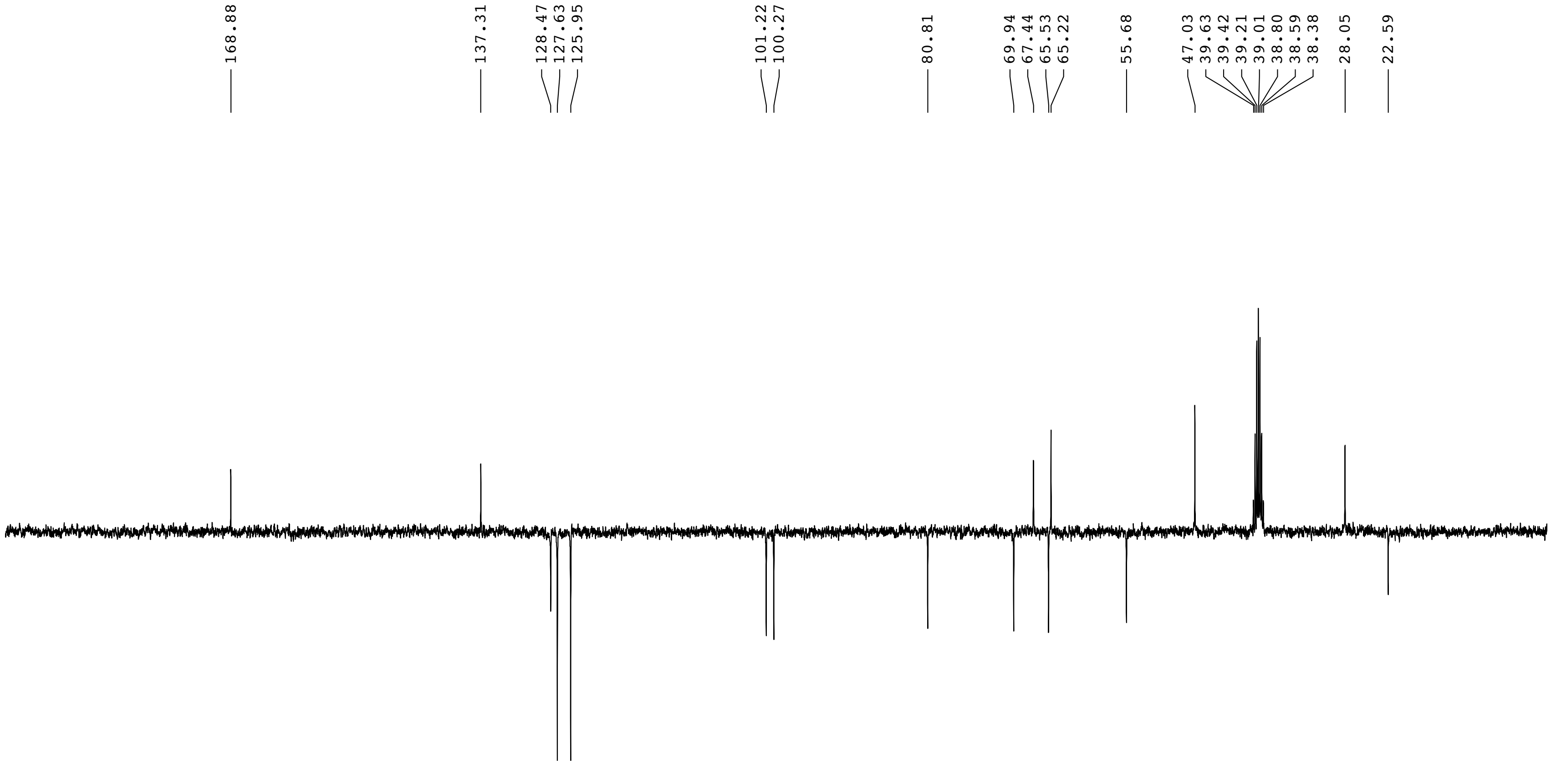

NAME	0906Marian
EXPNO	13
PROCNO	1
Date_	20090612
Time	9.49
INSTRUM	spect
PROBHD	5 mm PABBO BB-
PULPROG	jmod
TD	65536
SOLVENT	DMSO
NS	88
DS	1
SWH	24038.461 H
FIDRES	0.366798 H
AQ	1.3631988 s
RG	2050
DW	20.800 u
DE	6.00 u
TE	295.9 K
CNST2	155.0000000
CNST11	1.0000000
D1	1.50000000 s
D20	0.00645161 s
TD0	1

======== CHANNEL f1 =====

NUC1	13C
P1	9.10 u
P2	18.20 u
PL1	-1.00 d
PL1W	44.27188873 W
SFO1	100.6479773 M

======== CHANNEL f2 =====

CPDPRG2	waltz16
NUC2	1H
PCPD2	80.00 u
PL2	0.00 d
PL12	15.00 d
PL2W	8.41481972 W
PL12W	0.26609996 W
SFO2	400.2316009 M
SI	32768
SF	100.6380019 M
WDW	EM
SSB	0
LB	2.50 H
GB	0
PC	1.40

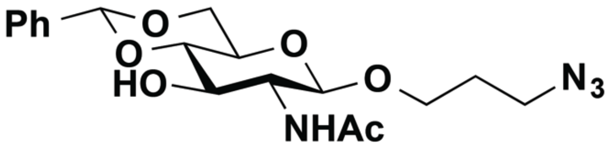

9

S7

1h NMR av400liq BvB 7

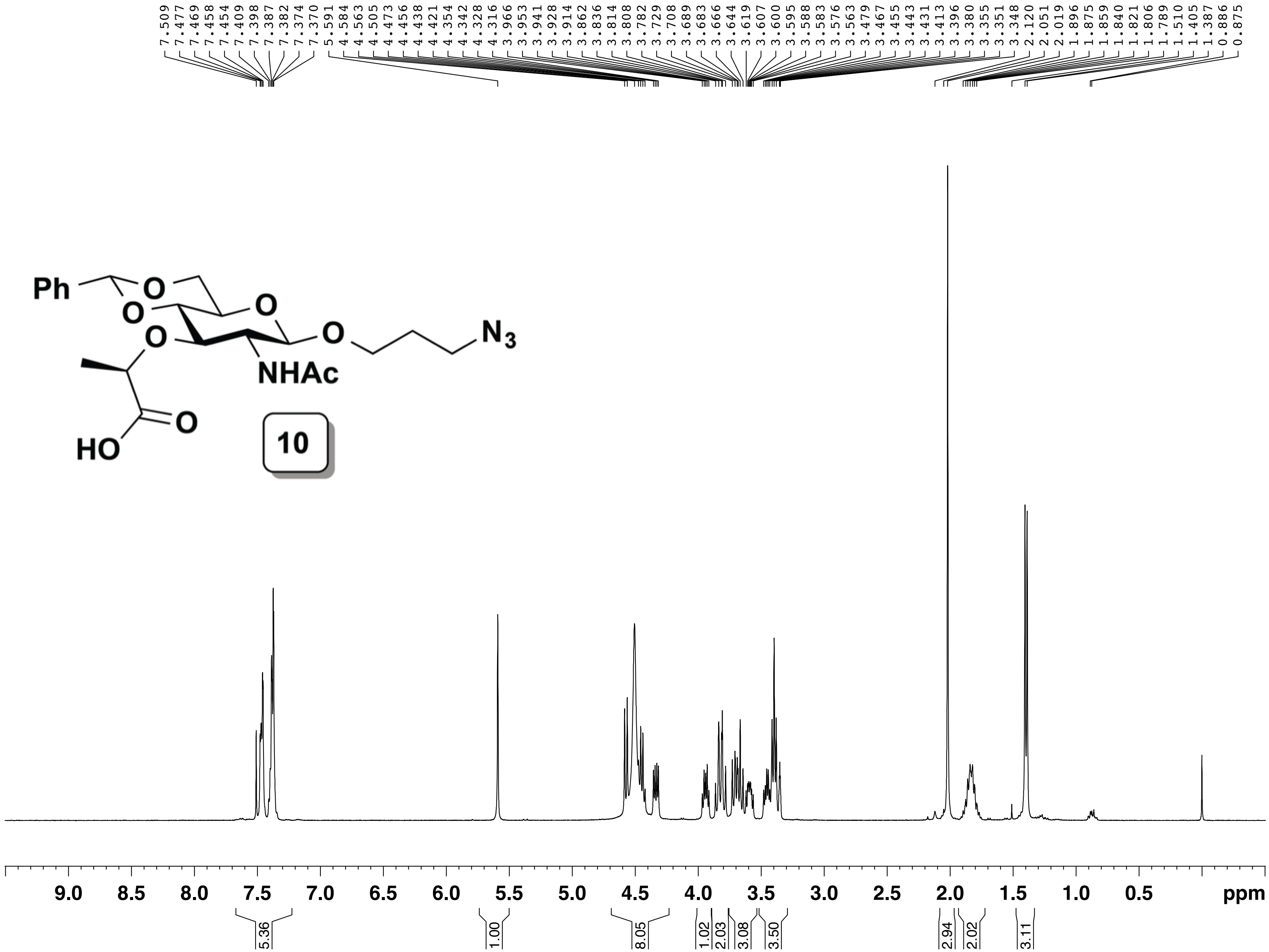

10

NAME	1006Bianca
EXPNO	17
PROCNO	1
Date_	20100603
Time	16.40
INSTRUM	spect
PROBHD	5 mm PABBO BB-
PULPROG	zg30
TD	65536
SOLVENT	MeOD
NS	8
DS	0
SWH	8223.685 Hz
FIDRES	0.125483 Hz
AQ	3.9846387 sec
RG	161
DW	60.800 use
DE	6.50 use
TE	300.3 K
D1	1.00000000 sec
TD0	1

======== CHANNEL f1 =======

NUC1	1H
P1	15.00 use
PL1	-3.00 dB
PL1W	16.78977203 W
SFO1	400.2324716 MHz
SI	65536
SF	400.2299939 MHz
WDW	EM
SSB	0
LB	0.30 Hz
GB	0
PC	1.00

BvB 7
biosynAPTfast MeOD /opt/DATA nmrafd 4

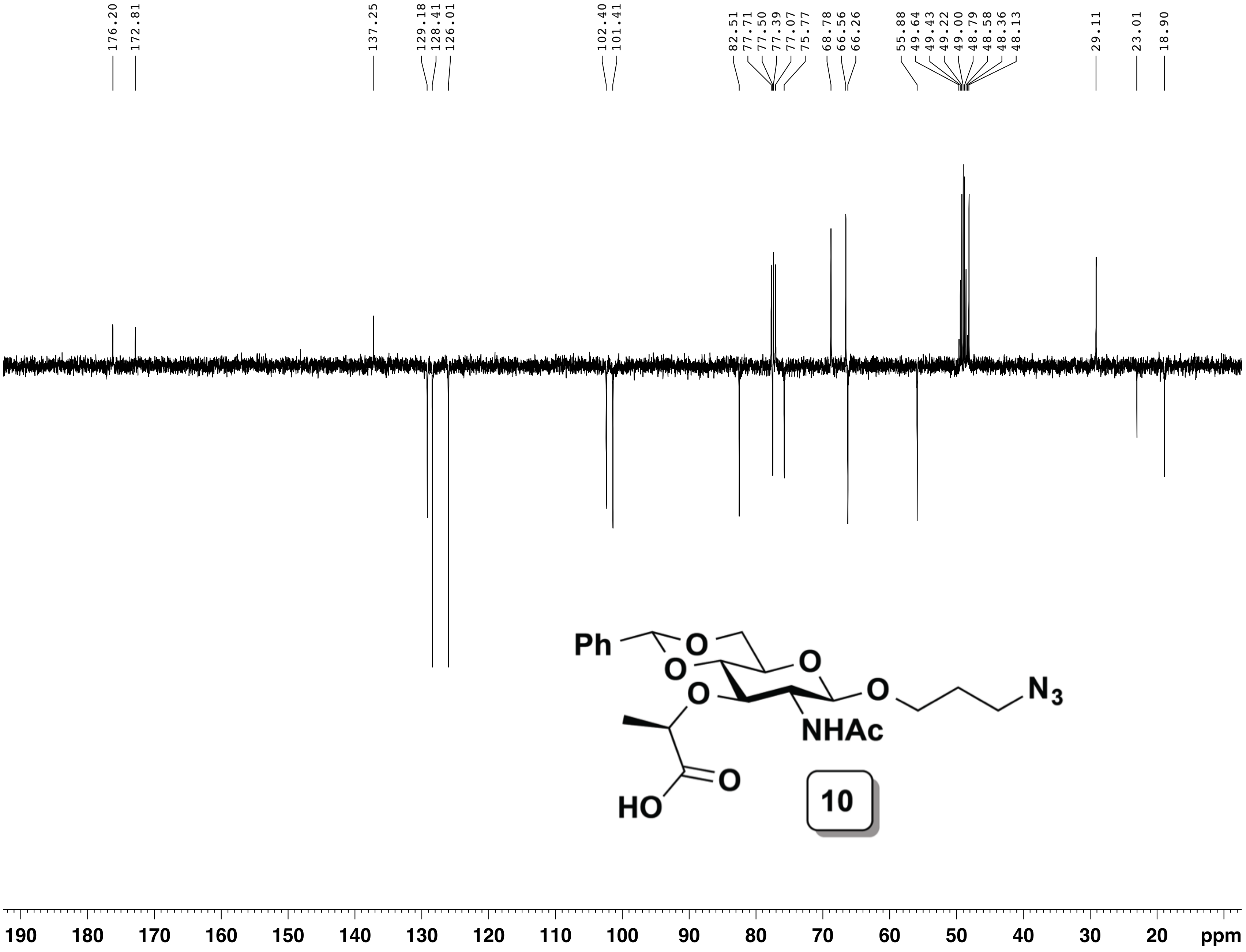

NAME	1006Bianca	
EXPNO	10	
PROCNO	1	
Date_	20100602	
Time	23.52	
INSTRUM	spect	
PROBHD	5 mm PABBO BB-	
PULPROG	jmod	
TD	65536	
SOLVENT	MeOD	
NS	256	
DS	1	
SWH	24038.461	H
FIDRES	0.366798	H
AQ	1.3631988	se
RG	2050	
DW	20.800	u
DE	6.00	u
TE	301.2	K
CNST2	155.0000000	
CNST11	1.0000000	
D1	1.50000000	se
D20	0.00645161	se
TD0	1	
======== CHANNEL f1 ======		
NUC1	13C	
P1	9.10	u
P2	18.20	u
PL1	-1.00	d
PL1W	44.27188873	W
SFO1	100.6479773	M
======== CHANNEL f2 ======		
CPDPRG2	waltz16	
NUC2	1H	
PCPD2	80.00	u
PL2	-3.00	d
PL12	11.89	d
PL2W	16.78977203	W
PL12W	0.54455876	W
SFO2	400.2316009	M
SI	32768	
SF	100.6378953	M
WDW	EM	
SSB	0	
LB	1.00	H
GB	0	
PC	1.40	

S9

1h NMR av400liq mw308

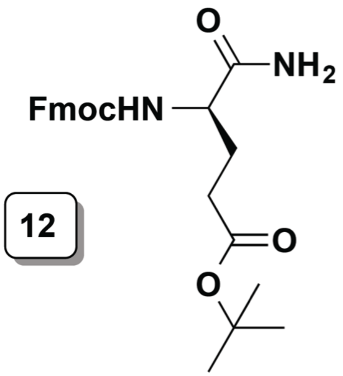

12

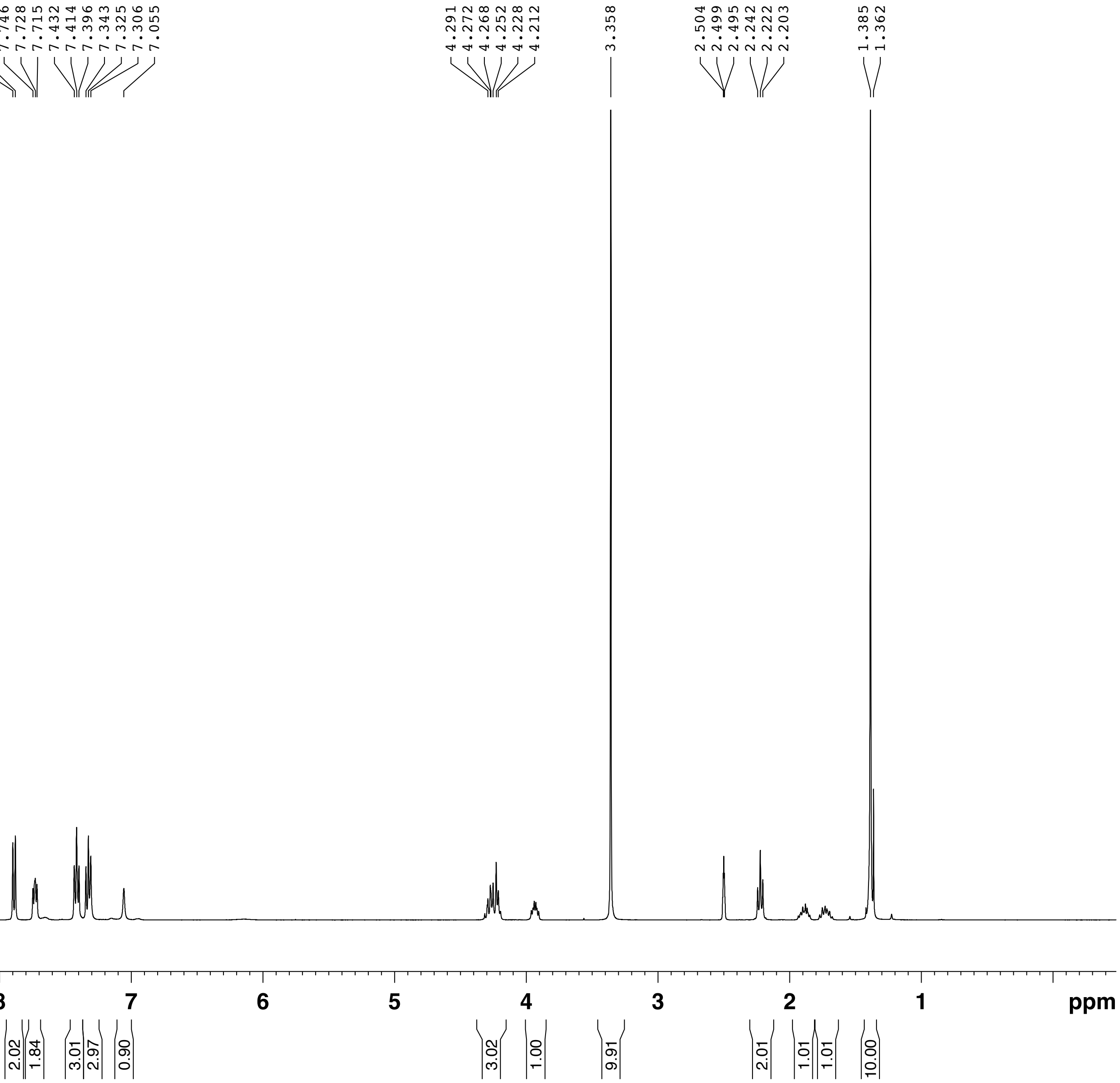


```
NAME          1009Marian
EXPNO                  6
PROCNO                 1
Date_           20100906
Time               13.12
INSTRUM            spect
PROBHD   5 mm PABBO BB-
PULPROG             zg30
TD                 65536
SOLVENT             DMSO
NS                    14
DS                     0
SWH             8223.685 Hz
FIDRES          0.125483 Hz
AQ             3.9846387 sec
RG                   144
DW                60.800 use
DE                  6.50 use
TE                 295.8 K
D1            1.00000000 sec
TD0                    1

======== CHANNEL f1 =======
NUC1                  1H
P1                 14.40 use
PL1                 0.00 dB
PL1W          8.41481972 W
SFO1         400.2324716 MHz
SI                 65536
SF           400.2300051 MHz
WDW                   EM
SSB                    0
LB                  0.30 Hz
GB                     0
PC                  1.00
```

S10

13C APT av400liq mw308

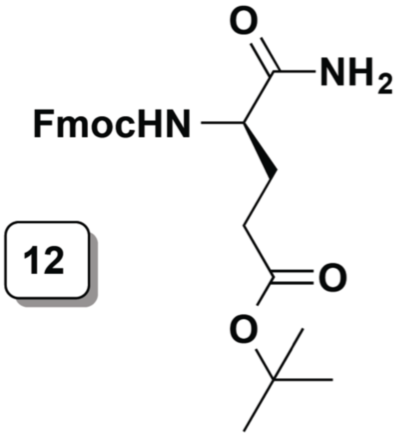

12

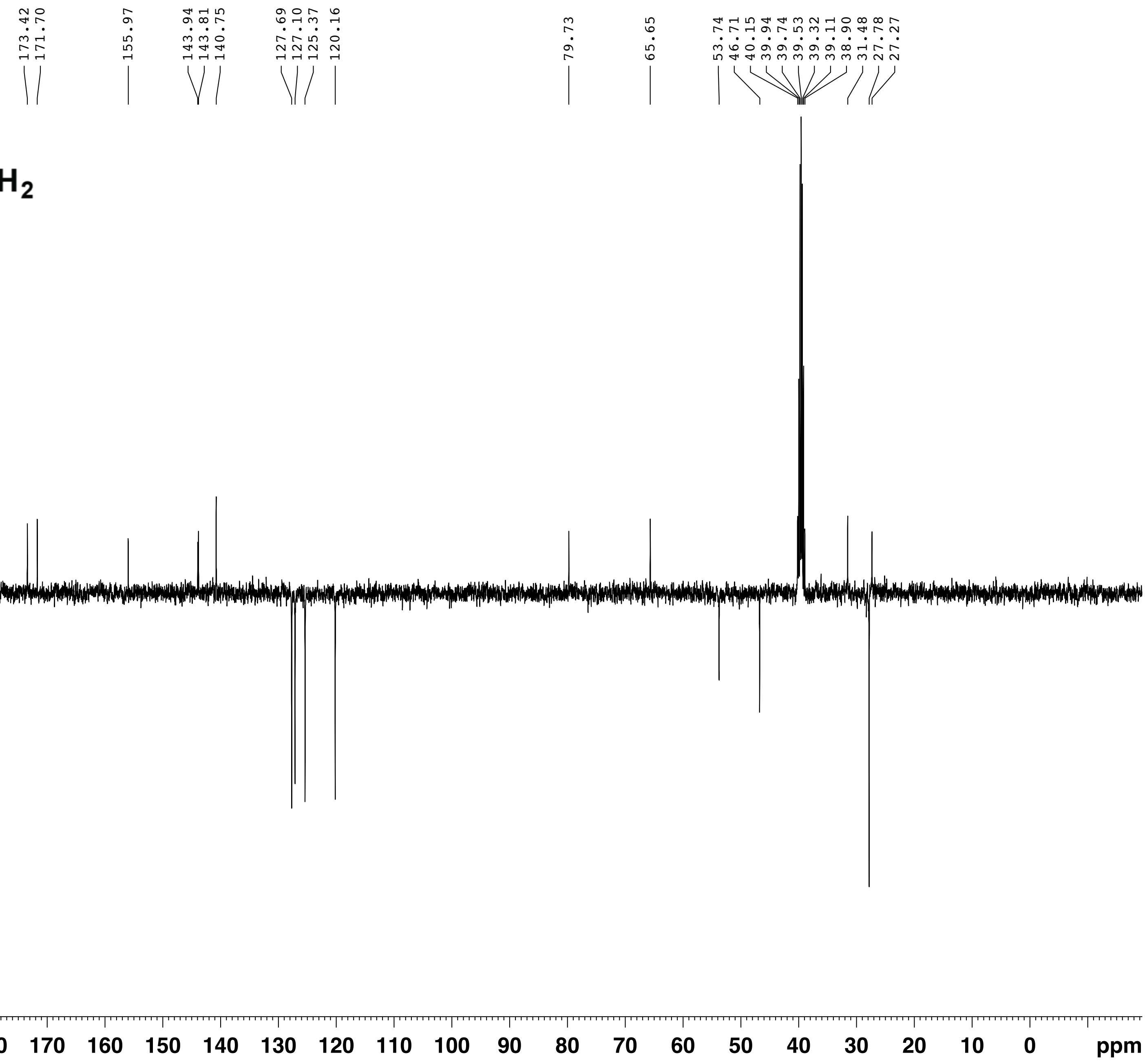

NAME	1009Marian
EXPNO	7
PROCNO	1
Date_	20100906
Time	13.15
INSTRUM	spect
PROBHD	5 mm PABBO BB-
PULPROG	jmod
TD	65536
SOLVENT	DMSO
NS	114
DS	0
SWH	24038.461 Hz
FIDRES	0.366798 Hz
AQ	1.3631988 se
RG	2050
DW	20.800 us
DE	6.00 us
TE	296.5 K
CNST2	155.0000000
CNST11	1.0000000
D1	1.50000000 se
D20	0.00645161 se
TD0	1
======== CHANNEL f1 ======	
NUC1	13C
P1	9.10 us
P2	18.20 us
PL1	-1.00 dB
PL1W	44.27188873 W
SFO1	100.6479773 MH
======== CHANNEL f2 ======	
CPDPRG2	waltz16
NUC2	1H
PCPD2	80.00 us
PL2	0.00 dB
PL12	14.89 dB
PL2W	8.41481972 W
PL12W	0.27292591 W
SFO2	400.2316009 MH
SI	32768
SF	100.6379571 MH
WDW	EM
SSB	0
LB	2.50 Hz
GB	0
PC	1.40

1h NMR av400liq mw313

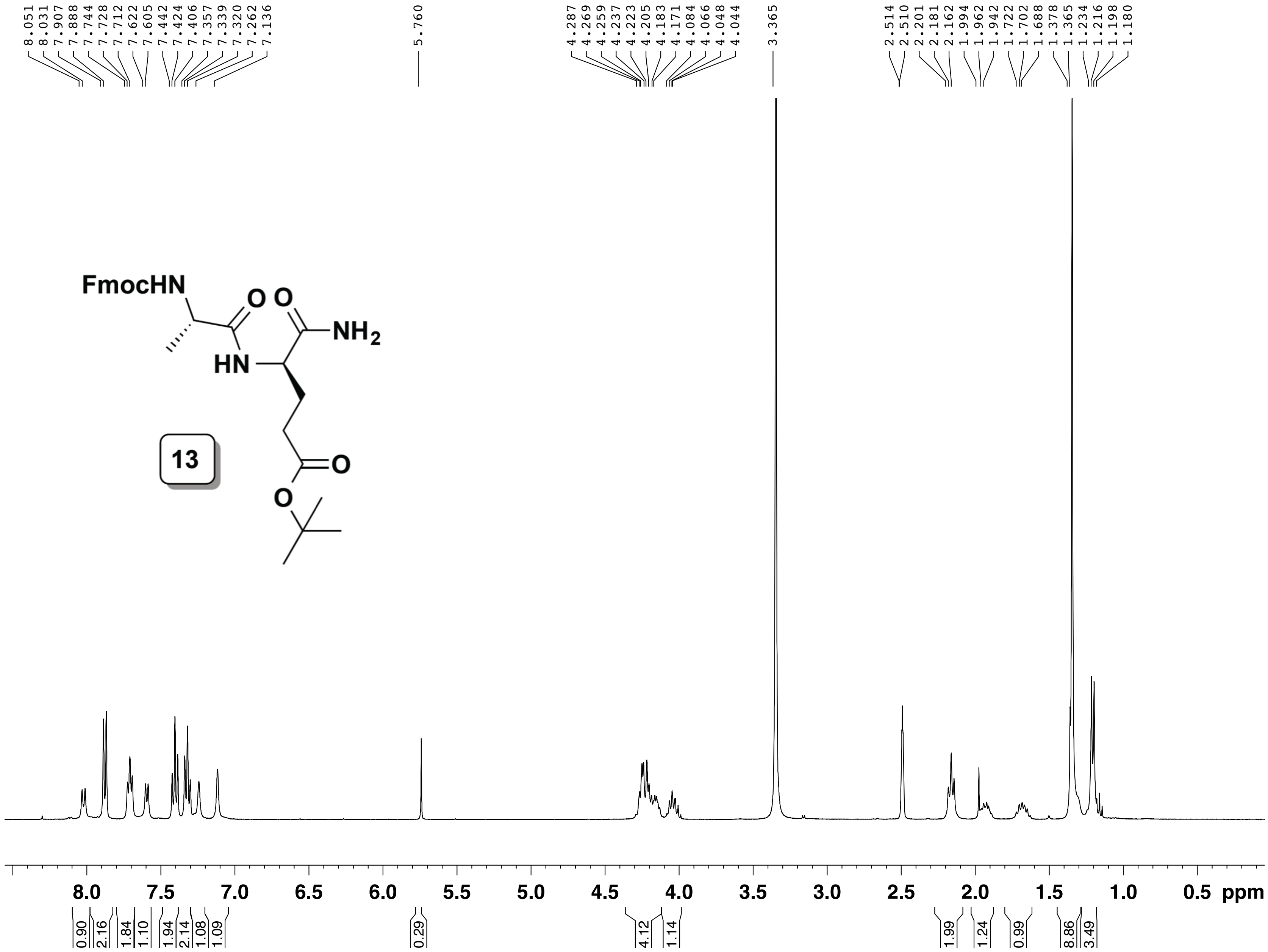

13

NAME	1009Marian
EXPNO	27
PROCNO	1
Date_	20100913
Time	13.26
INSTRUM	spect
PROBHD	5 mm PABBO BB-
PULPROG	zg30
TD	65536
SOLVENT	DMSO
NS	8
DS	0
SWH	8223.685 Hz
FIDRES	0.125483 Hz
AQ	3.9846387 sec
RG	144
DW	60.800 use
DE	6.50 use
TE	297.1 K
D1	1.00000000 sec
TD0	1

======== CHANNEL f1 =======

NUC1	1H
P1	14.40 use
PL1	0.00 dB
PL1W	8.41481972 W
SFO1	400.2324716 MHz
SI	65536
SF	400.2300086 MHz
WDW	EM
SSB	0
LB	0.30 Hz
GB	0
PC	1.00

13C APT av400liq mw313

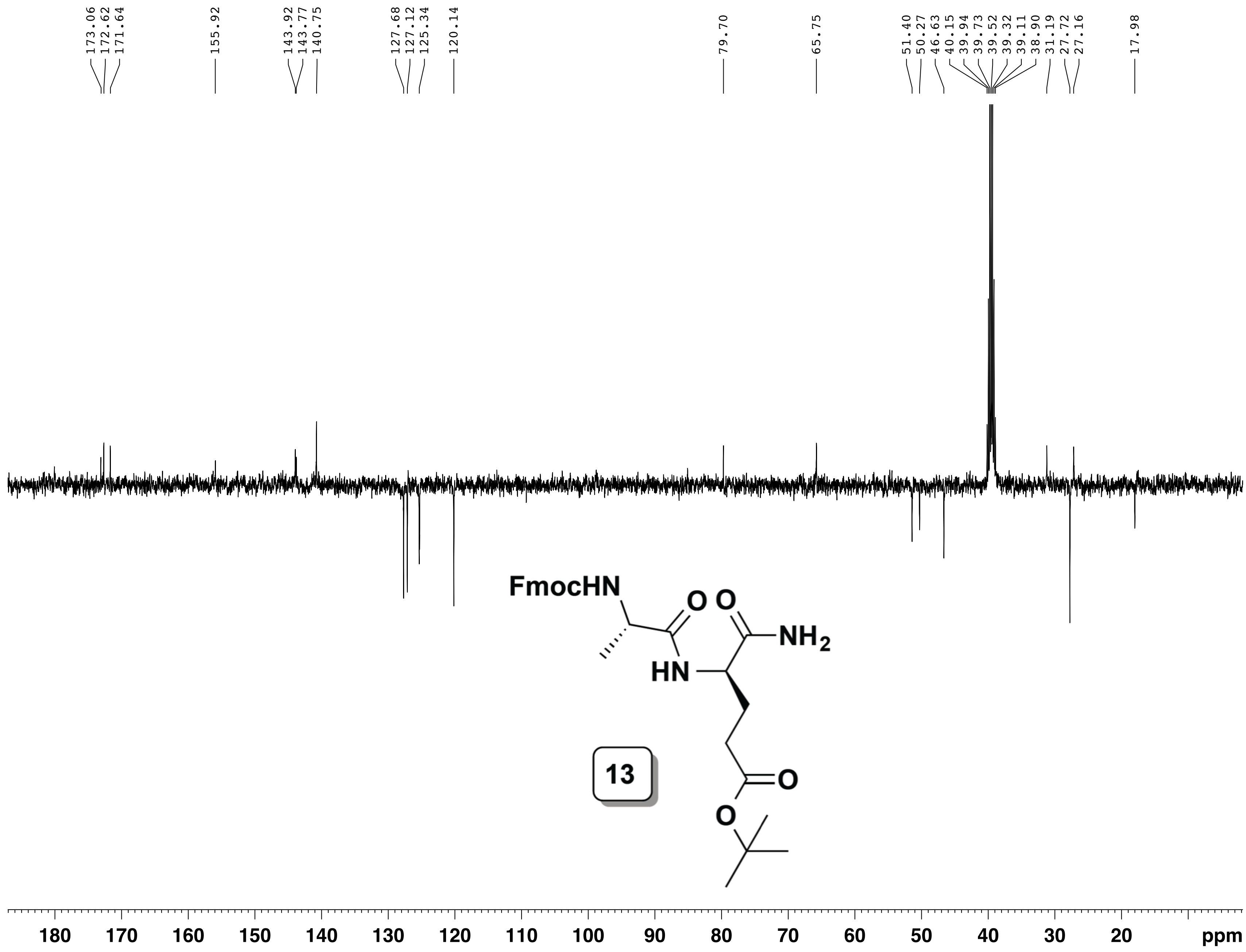

NAME	1009Marian
EXPNO	28
PROCNO	1
Date_	20100913
Time	13.29
INSTRUM	spect
PROBHD	5 mm PABBO BB-
PULPROG	jmod
TD	65536
SOLVENT	DMSO
NS	81
DS	0
SWH	24038.461 H
FIDRES	0.366798 H
AQ	1.3631988 s
RG	2050
DW	20.800 u
DE	6.00 u
TE	297.9 K
CNST2	155.0000000
CNST11	1.0000000
D1	1.50000000 s
D20	0.00645161 s
TD0	1
======== CHANNEL f1 =====	
NUC1	13C
P1	9.10 u
P2	18.20 u
PL1	-1.00 d
PL1W	44.27188873 W
SFO1	100.6479773 M
======== CHANNEL f2 =====	
CPDPRG2	waltz16
NUC2	1H
PCPD2	80.00 u
PL2	0.00 d
PL12	14.89 d
PL2W	8.41481972 W
PL12W	0.27292591 W
SFO2	400.2316009 M
SI	32768
SF	100.6379578 M
WDW	EM
SSB	0
LB	2.50 H
GB	0
PC	1.40

mw295
biosyn1Hfast MeOD /opt/DATA nmrafd 1

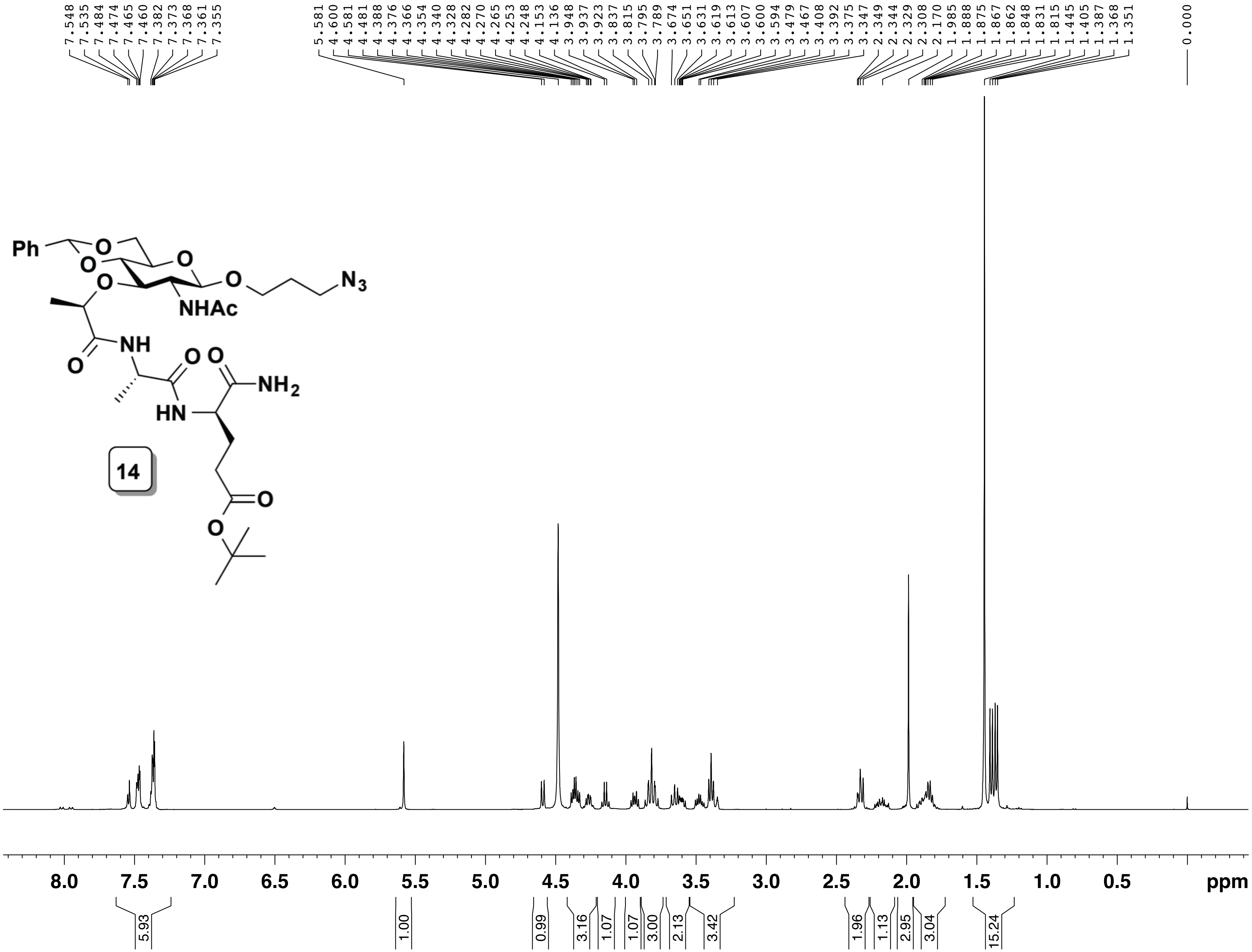

NAME	1008Marian
EXPNO	84
PROCNO	1
Date_	20100825
Time	17.24
INSTRUM	spect
PROBHD	5 mm PABBO BB-
PULPROG	zg30
TD	65536
SOLVENT	MeOD
NS	16
DS	1
SWH	8223.685 Hz
FIDRES	0.125483 Hz
AQ	3.9846387 sec
RG	80.6
DW	60.800 use
DE	6.50 use
TE	300.0 K
D1	1.00000000 sec
TD0	1

======== CHANNEL f1 =======

NUC1	1H
P1	14.10 use
PL1	0.00 dB
PL1W	8.41481972 W
SFO1	400.2324716 MHz
SI	65536
SF	400.2299953 MHz
WDW	EM
SSB	0
LB	0.30 Hz
GB	0
PC	1.00

mw295
biosyn1Hfast MeOD /opt/DATA nmrafd 1

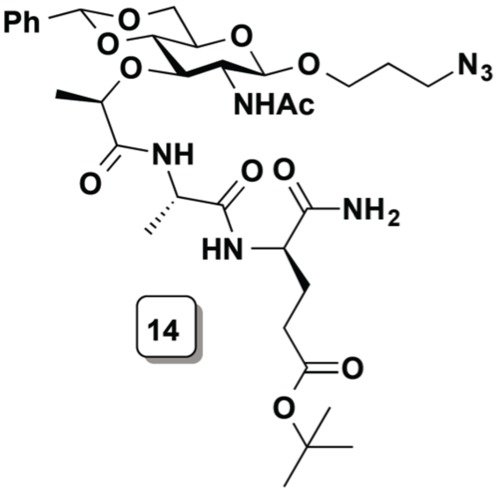

14

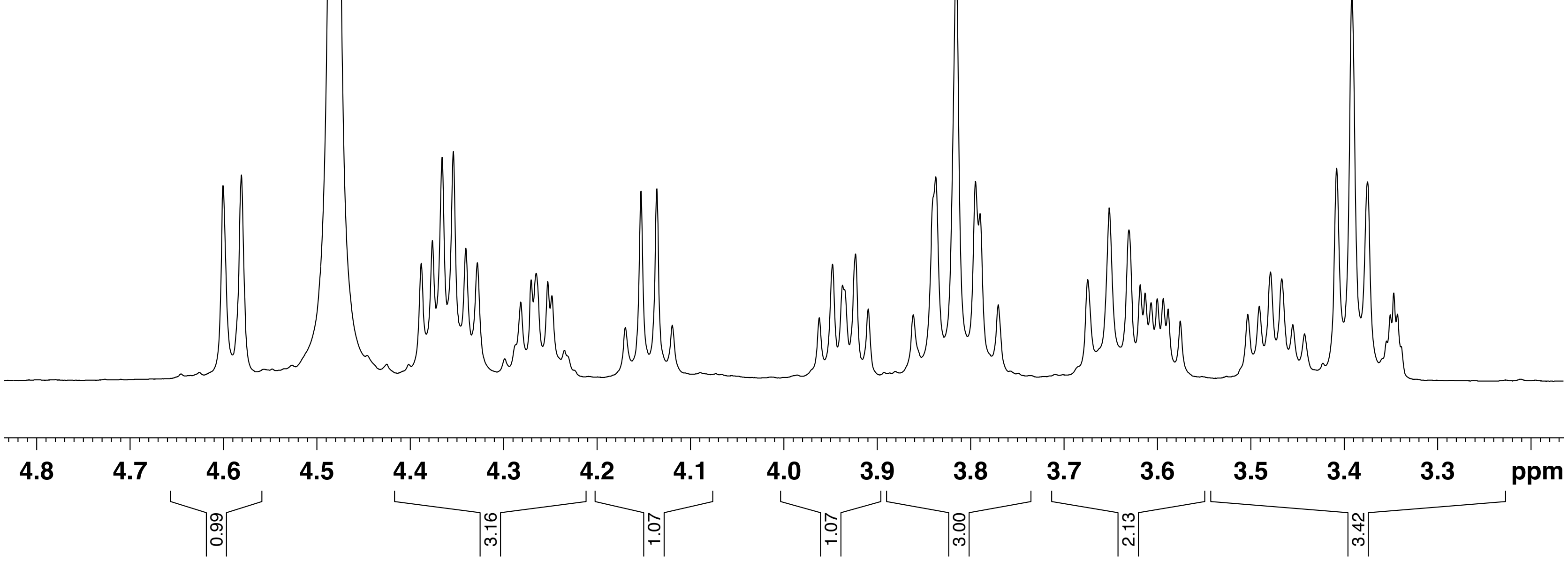

NAME	1008Marian
EXPNO	84
PROCNO	1
Date_	20100825
Time	17.24
INSTRUM	spect
PROBHD	5 mm PABBO BB-
PULPROG	zg30
TD	65536
SOLVENT	MeOD
NS	16
DS	1
SWH	8223.685 Hz
FIDRES	0.125483 Hz
AQ	3.9846387 sec
RG	80.6
DW	60.800 use
DE	6.50 use
TE	300.0 K
D1	1.00000000 sec
TD0	1

======== CHANNEL f1 =======

NUC1	1H
P1	14.10 use
PL1	0.00 dB
PL1W	8.41481972 W
SFO1	400.2324716 MHz
SI	65536
SF	400.2299953 MHz
WDW	EM
SSB	0
LB	0.30 Hz
GB	0
PC	1.00

mw295
biosynAPTfast MeOD /opt/DATA nmrafd 1

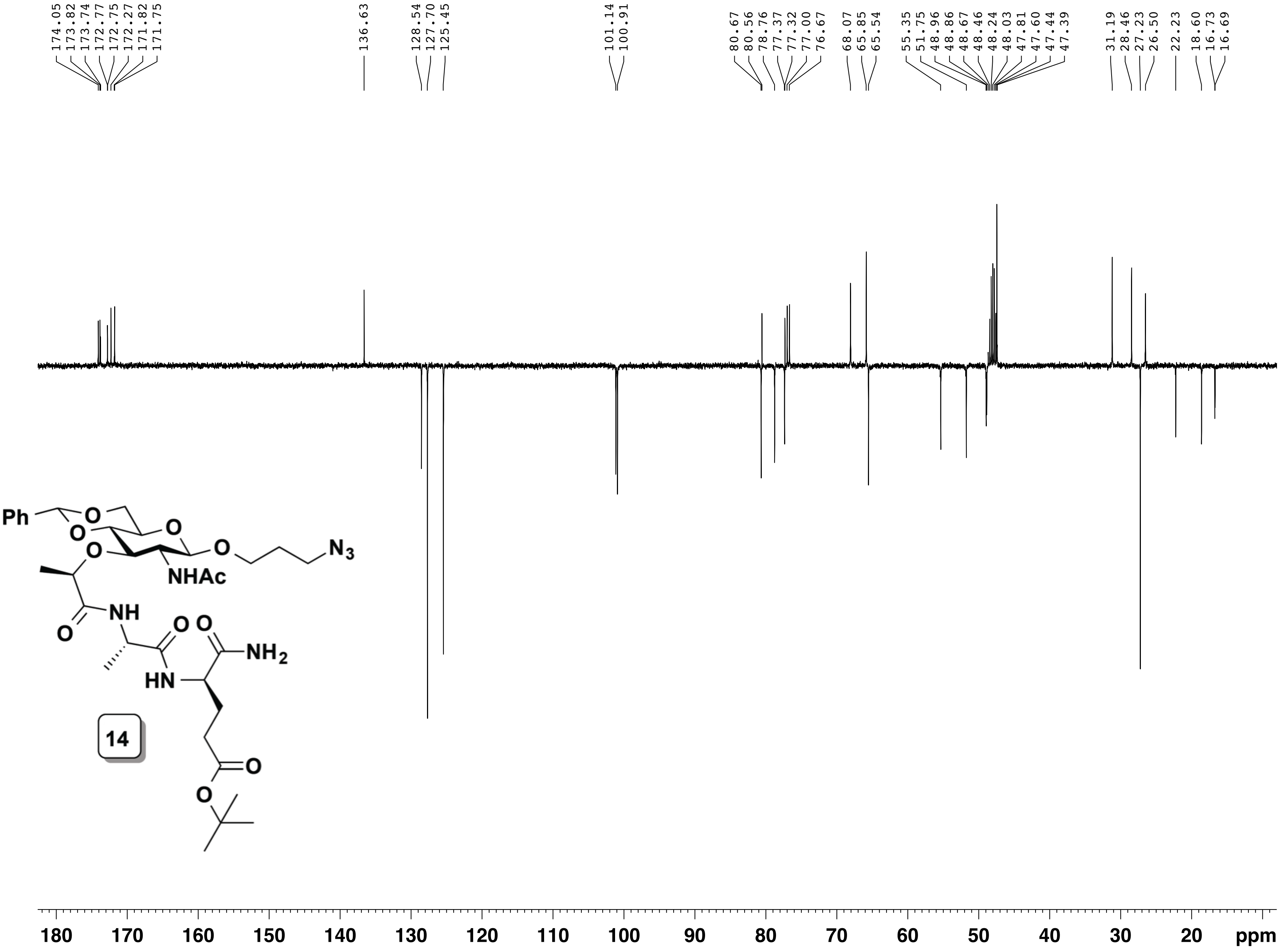

NAME	1008Marian
EXPNO	85
PROCNO	1
Date_	20100825
Time	17.50
INSTRUM	spect
PROBHD	5 mm PABBO BB-
PULPROG	jmod
TD	65536
SOLVENT	MeOD
NS	512
DS	1
SWH	24038.461 H
FIDRES	0.366798 H
AQ	1.3631988 s
RG	2050
DW	20.800 u
DE	6.00 u
TE	300.2 K
CNST2	155.0000000
CNST11	1.0000000
D1	1.50000000 s
D20	0.00645161 s
TD0	1
======== CHANNEL f1 =====	
NUC1	13C
P1	9.10 u
P2	18.20 u
PL1	-1.00 d
PL1W	44.27188873 W
SFO1	100.6479773 M
======== CHANNEL f2 =====	
CPDPRG2	waltz16
NUC2	1H
PCPD2	80.00 u
PL2	0.00 d
PL12	15.00 d
PL2W	8.41481972 W
PL12W	0.26609996 W
SFO2	400.2316009 M
SI	32768
SF	100.6379580 M
WDW	EM
SSB	0
LB	1.00 H
GB	0
PC	1.40

mw396
biosyn1Hfast DMSO /opt/DATA nmrafd 8

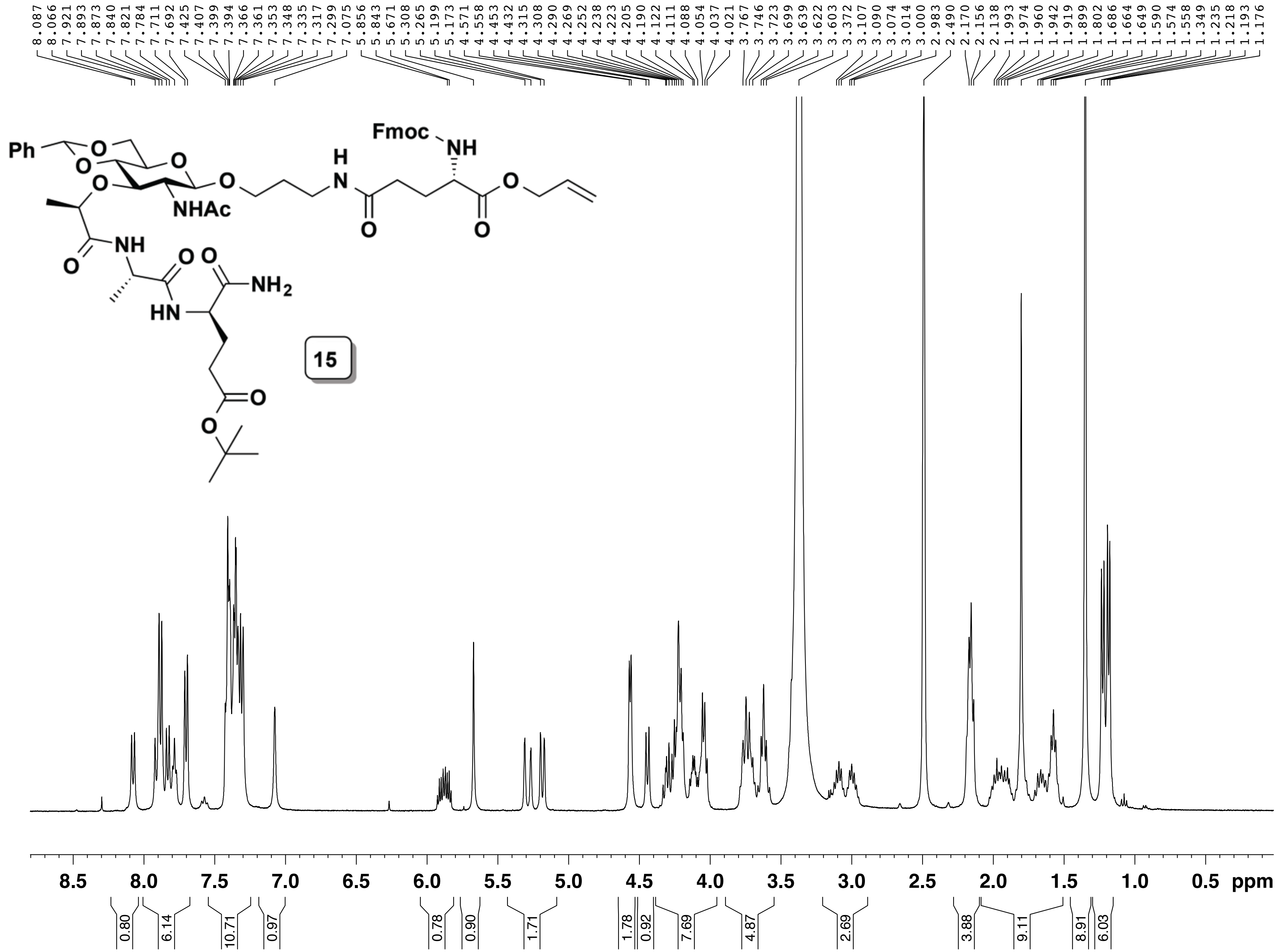

NAME	1103Marian
EXPNO	69
PROCNO	1
Date_	20110331
Time	2.16
INSTRUM	spect
PROBHD	5 mm PABBO BB-
PULPROG	zg30
TD	65536
SOLVENT	DMSO
NS	64
DS	1
SWH	8223.685 Hz
FIDRES	0.125483 Hz
AQ	3.9846387 sec
RG	144
DW	60.800 use
DE	6.50 use
TE	295.4 K
D1	1.00000000 sec
TD0	1

======== CHANNEL f1 =======

NUC1	1H
P1	14.40 use
PL1	0.00 dB
PL1W	8.41481972 W
SFO1	400.2324716 MHz
SI	65536
SF	400.2300085 MHz
WDW	EM
SSB	0
LB	0.30 Hz
GB	0
PC	1.00

mw342
biosynAPTfast DMSO /opt/DATA nmrafd 8

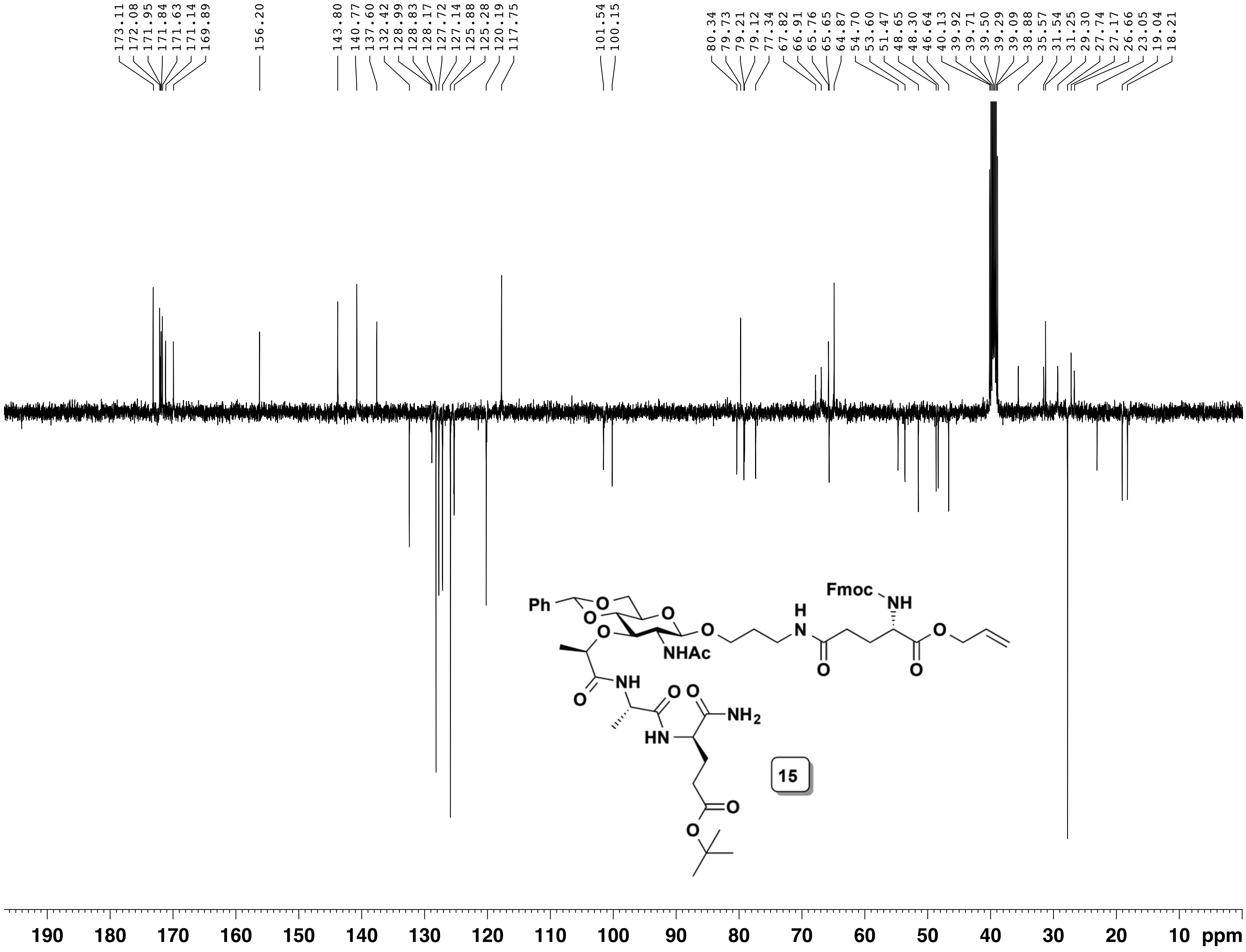

NAME	1011Marian
EXPNO	33
PROCNO	1
Date_	20101117
Time	4.32
INSTRUM	spect
PROBHD	5 mm PABBO BB-
PULPROG	jmod
TD	65536
SOLVENT	DMSO
NS	3072
DS	1
SWH	24038.461 H
FIDRES	0.366798 H
AQ	1.3631988 s
RG	2050
DW	20.800 u
DE	6.00 u
TE	295.0 K
CNST2	155.0000000
CNST11	1.0000000
D1	1.50000000 s
D20	0.00645161 s
TD0	1

======== CHANNEL f1 ======

NUC1	13C
P1	9.10 u
P2	18.20 u
PL1	-1.00 d
PL1W	44.27188873 W
SFO1	100.6479773 M

======== CHANNEL f2 ======

CPDPRG2	waltz16
NUC2	1H
PCPD2	80.00 u
PL2	0.00 d
PL12	15.00 d
PL2W	8.41481972 W
PL12W	0.26609996 W
SFO2	400.2316009 M
SI	32768
SF	100.6379561 M
WDW	EM
SSB	0
LB	1.00 H
GB	0
PC	1.40

presat GP dmx600   MW-401 in dmso(d6)   300K

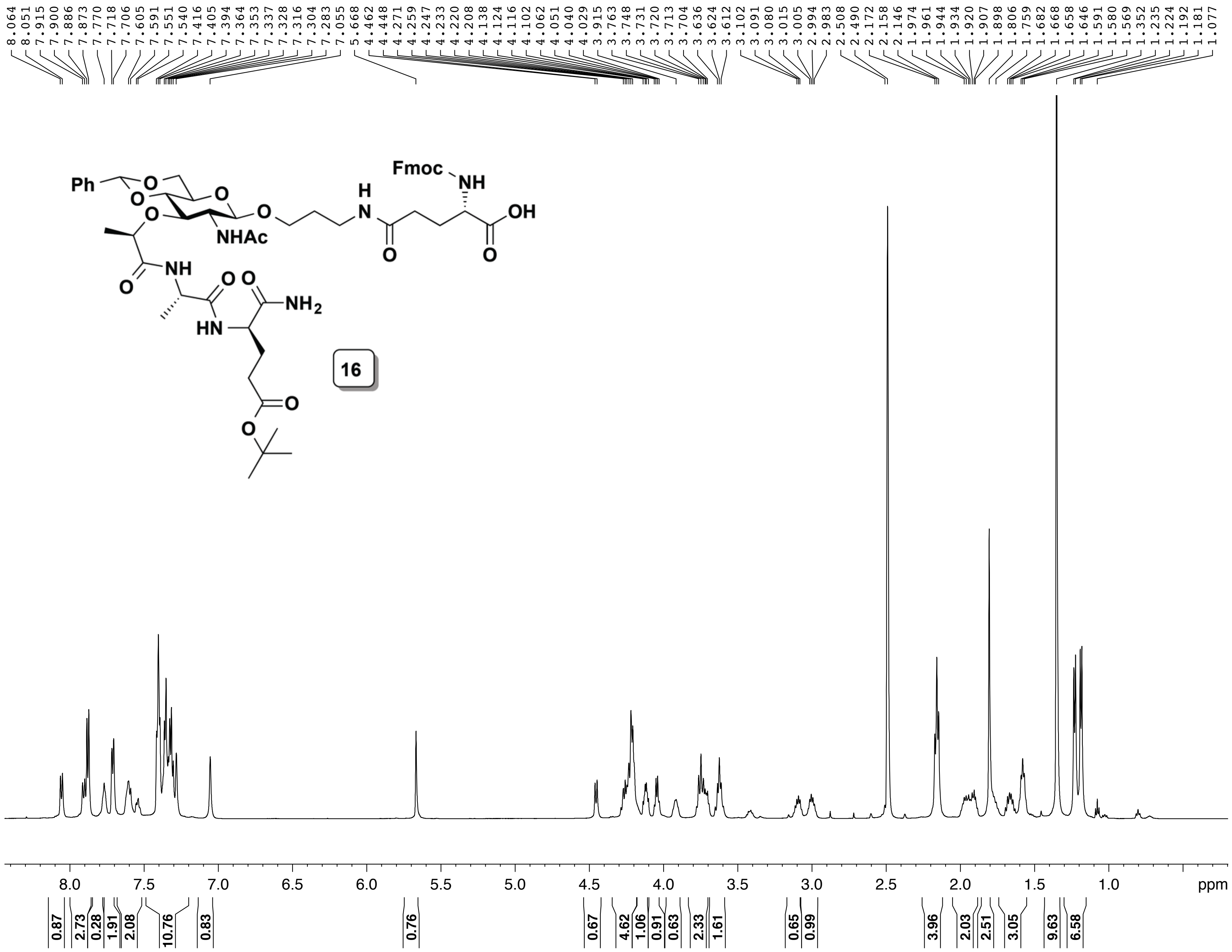

16

```
Current Data Parameters
NAME     biosyn042011Marian
EXPNO                 3
PROCNO                1

F2 - Acquisition Parameters
Date_          20110411
Time              10.06
INSTRUM           av600
PROBHD   5 mm CPTCI 1H-
PULPROG      noesygppr1d
TD                65536
SOLVENT            DMSO
NS                   16
DS                    4
SWH           12019.230 Hz
FIDRES         0.183399 Hz
AQ            2.7262976 sec
RG                   32
DW               41.600 use
DE                10.00 use
TE                305.5 K
D1           4.00000000 sec
D8           0.01000000 sec
D12          0.00002000 sec
D16          0.00020000 sec
ZGOPTNS

======== CHANNEL f1 =======
NUC1                 1H
P0                 8.76 use
P1                 8.76 use
PLW1         5.00029993 W
PLW9         0.00000316 W
SFO1        600.1320258 MHz

====== GRADIENT CHANNEL ===
GPNAM[1]      SMSQ10.100
GPNAM[2]      SMSQ10.100
GPZ1              50.00 %
GPZ2             -10.00 %
P16             1000.00 use

F2 - Processing parameters
SI                65536
SF          600.1300144 MHz
WDW                  EM
SSB      0
LB                 0.30 Hz
GB       0
PC                 1.00
```

13C APT av-600 MW-401 in dmso(d6) 300K

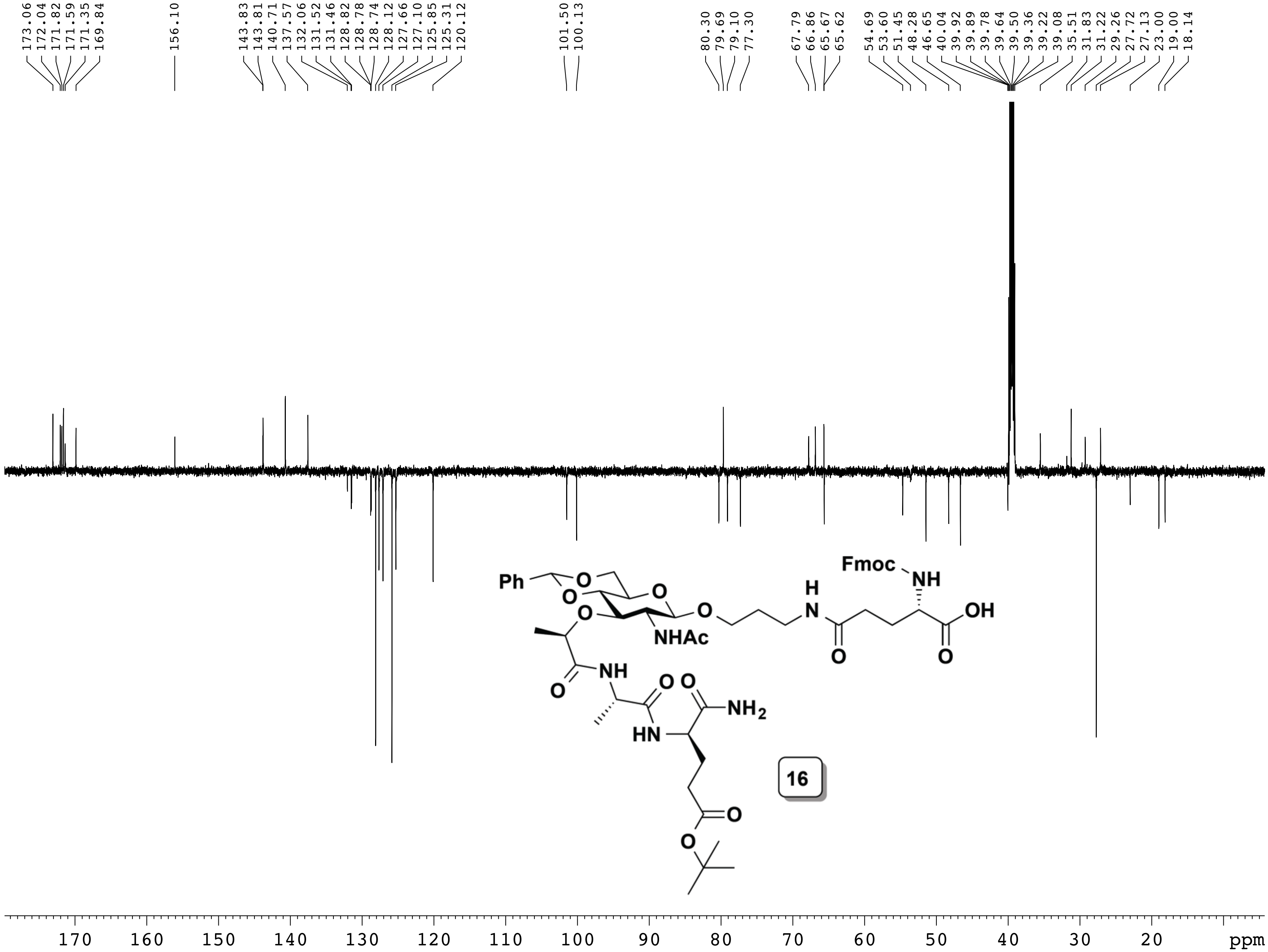

Current Data Parameters
NAME biosyn042011Maria
EXPNO 2
PROCNO 1

F2 - Acquisition Parameter
Date_ 20110411
Time 9.08
INSTRUM av600
PROBHD 5 mm CPTCI 1H-
PULPROG jmod
TD 131072
SOLVENT DMSO
NS 777
DS 0
SWH 34722.223 Hz
FIDRES 0.264910 Hz
AQ 1.8874367 se
RG 2050
DW 14.400 us
DE 7.40 us
TE 305.5 K
CNST2 155.0000000
CNST11 1.0000000
D1 1.50000000 se
D20 0.00645161 se

======== CHANNEL f1 ======
NUC1 13C
P1 12.50 us
P2 25.00 us
PLW1 105.80000305 W
SFO1 150.9185940 MH

======== CHANNEL f2 ======
CPDPRG[2 waltz16
NUC2 1H
PCPD2 70.00 us
PLW2 5.00000000 W
PLW12 0.06530600 W
SFO2 600.1324000 MH

F2 - Processing parameters
SI 131072
SF 150.9028792 MH
WDW EM
SSB 0
LB 1.00 Hz
GB 0
PC 1.40

mw197 in dmso(d6)
PROTON DMSO /opt/topspin nmrafd 3

17

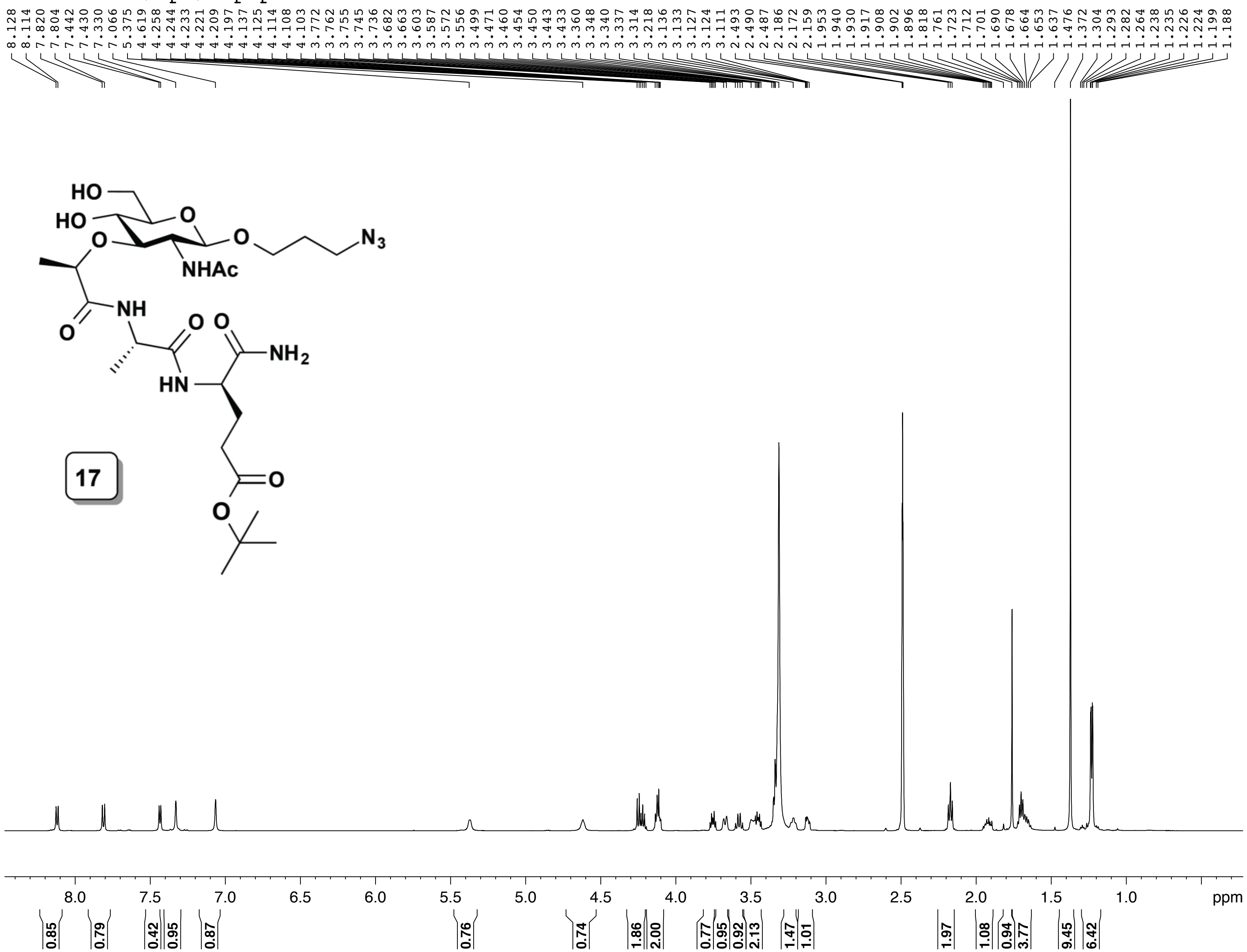


```
Current Data Parameters
NAME     biosyn112009Maria
EXPNO                   11
PROCNO                   1

F2 - Acquisition Parameter
Date_           20091110
Time                9.39
INSTRUM           dmx600
PROBHD   5 mm CPTCI 1H-
PULPROG             zg30
TD                 65536
SOLVENT             DMSO
NS                    64
DS                     2
SWH          12376.237 Hz
FIDRES        0.188846 Hz
AQ           2.6476543 se
RG                   128
DW              40.400 us
DE                8.50 us
TE               300.0 K
D1          1.00000000 se
TD0                    1

======== CHANNEL f1 =====
NUC1                  1H
P1                9.30 us
PL1              -6.00 dB
SFO1      600.1337060 MH

F2 - Processing parameters
SI                 32768
SF        600.1300129 MH
WDW                   EM
SSB      0
LB                0.30 Hz
GB       0
PC                 1.00
```

mw197 in dmso(d6)
C13APT DMSO /opt/topspin nmrafd 3

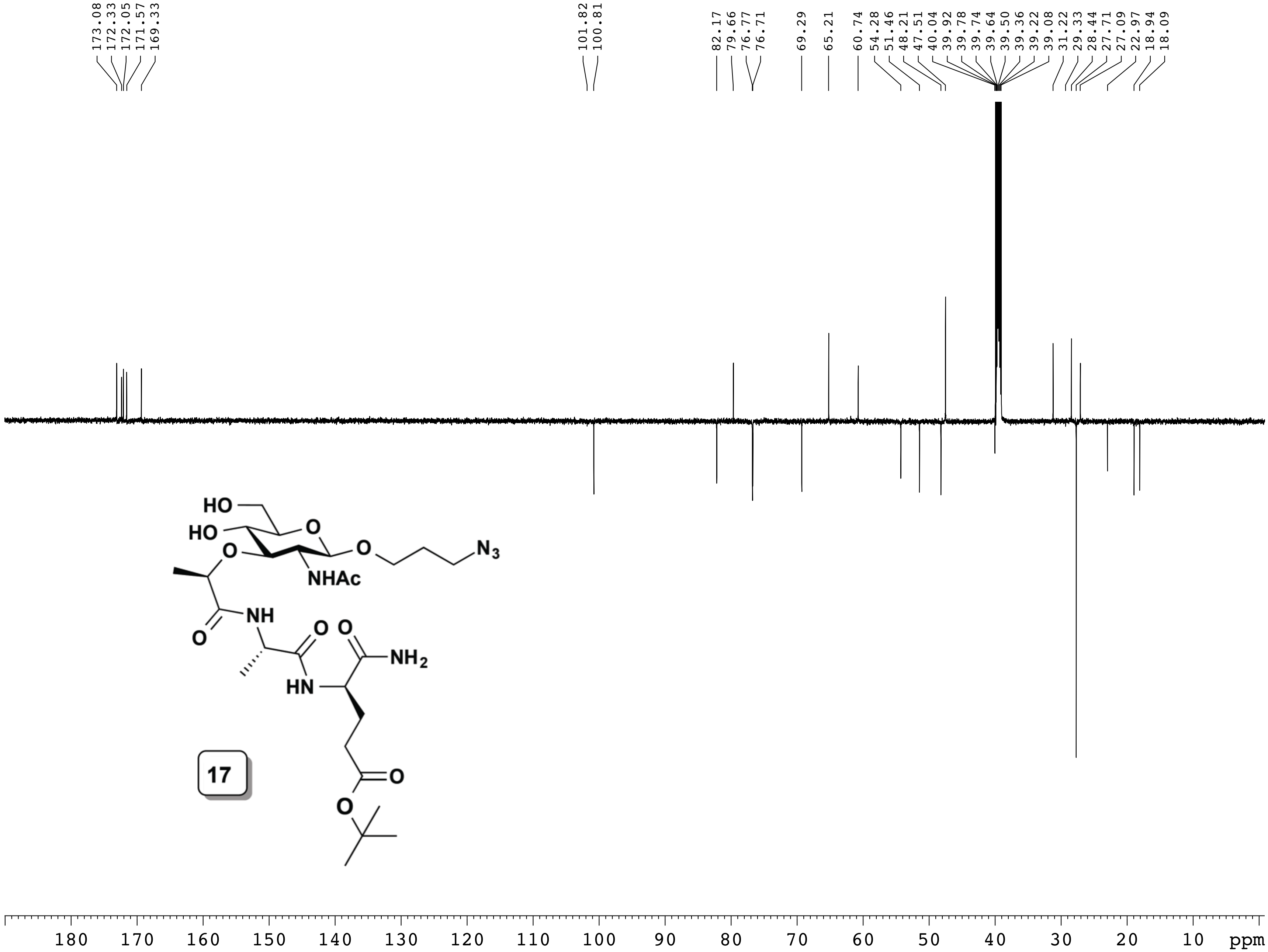

Current Data Parameters	
NAME	biosyn112009Maria
EXPNO	15
PROCNO	1
**F2 - Acquisition Parameter**	
Date_	20091110
Time	14.16
INSTRUM	dmx600
PROBHD	5 mm CPTCI 1H-
PULPROG	jmod
TD	65536
SOLVENT	DMSO
NS	8192
DS	4
SWH	35971.223 Hz
FIDRES	0.548877 Hz
AQ	0.9109504 se
RG	8192
DW	13.900 us
DE	8.50 us
TE	300.0 K
CNST2	145.0000000
CNST11	1.0000000
D1	2.00000000 se
d20	0.00689655 se
DELTA	0.00001592 se
TD0	8
======== CHANNEL f1 ======	
NUC1	13C
P1	12.50 us
p2	25.00 us
PL1	-6.00 dE
SFO1	150.9178988 MH
======== CHANNEL f2 ======	
CPDPRG[2	waltz16
NUC2	1H
PCPD2	80.00 us
PL2	-6.00 dE
PL12	12.69 dE
SFO2	600.1324005 MH
**F2 - Processing parameters**	
SI	32768
SF	150.9028829 MH
WDW	EM
SSB	0
LB	1.00 Hz
GB	0
PC	1.40

noesypr1d mw83 MDP-tBu

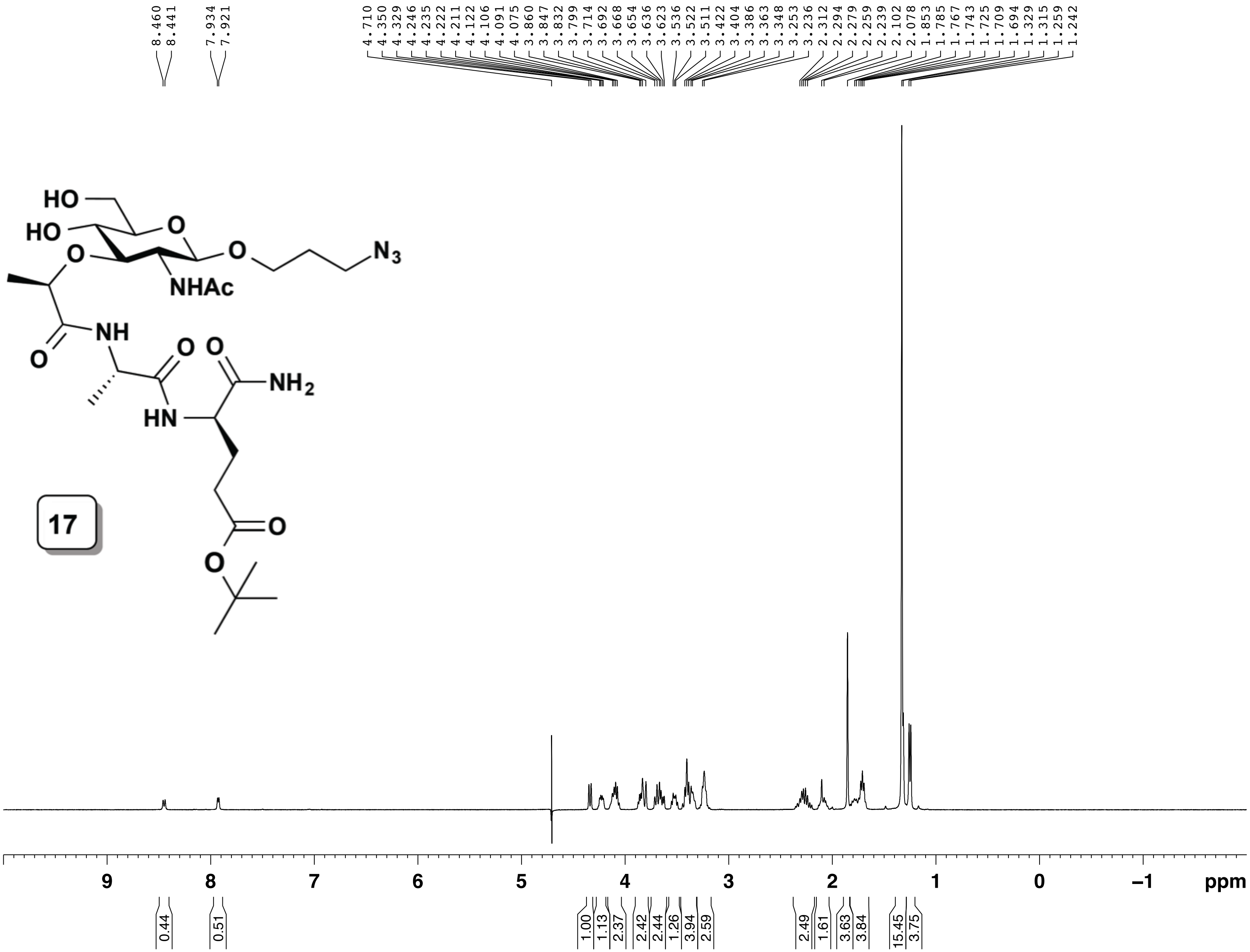

17

NAME	0810Marian
EXPNO	23
PROCNO	1
Date_	20081028
Time	10.03
INSTRUM	spect
PROBHD	5 mm PABBO BB-
PULPROG	noesypr1d
TD	32768
SOLVENT	D2O
NS	55
DS	4
SWH	6410.256 Hz
FIDRES	0.195625 Hz
AQ	2.5559540 sec
RG	812
DW	78.000 use
DE	6.00 use
TE	293.1 K
D1	1.00000000 sec
D8	0.05000000 sec
D11	0.03000000 sec
D12	0.00002000 sec
TD0	1

======== CHANNEL f1 =======

NUC1	1H
P1	14.10 use
PL1	0.00 dB
PL9	51.00 dB
PL1W	8.41481972 W
PL9W	0.00006684 W
SFO1	400.2318811 MHz
SI	65536
SF	400.2299980 MHz
WDW	EM
SSB	0
LB	0.30 Hz
GB	0
PC	1.00

noesypr1d mw83 MDP-tBu

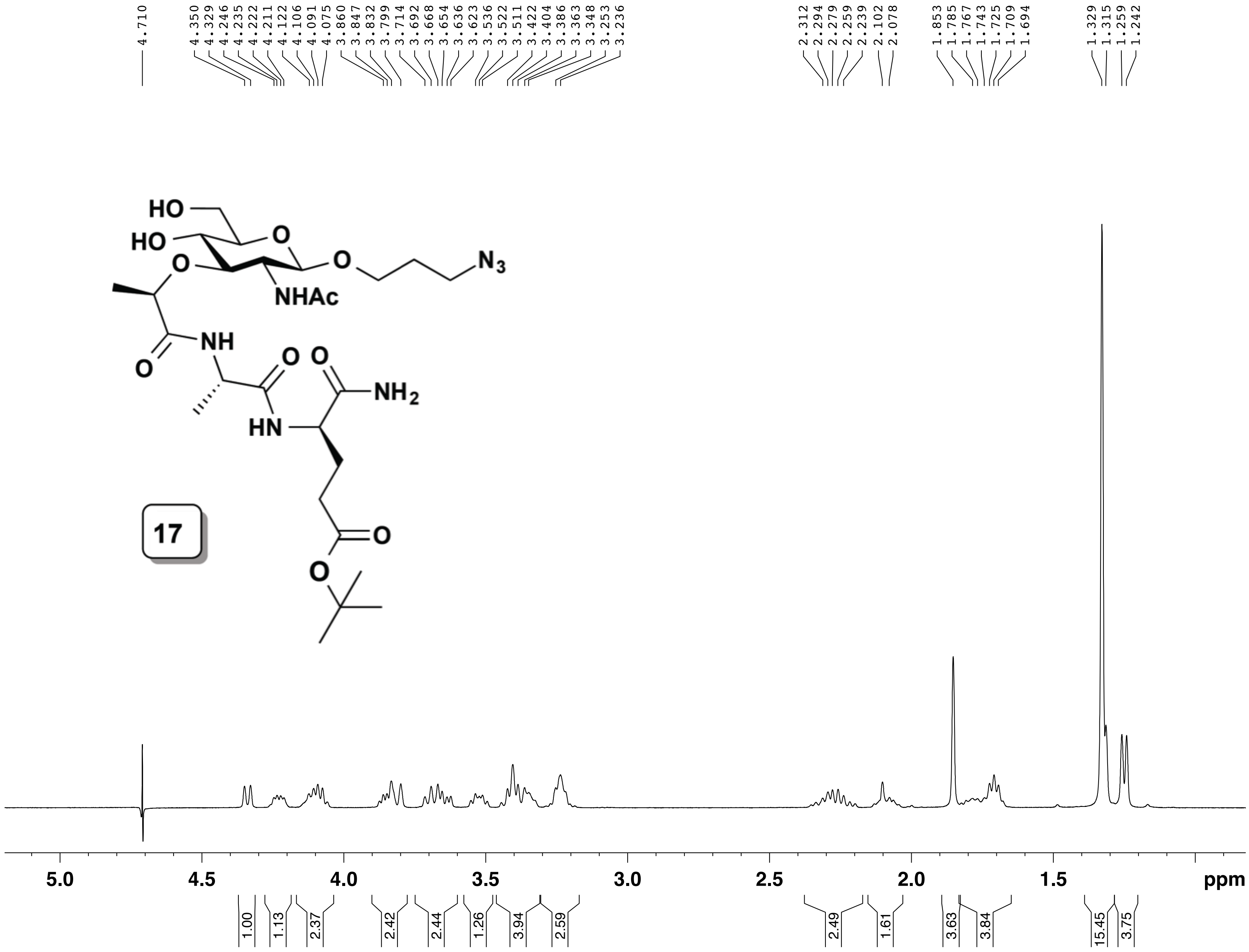


```
NAME          0810Marian
EXPNO                 23
PROCNO                 1
Date_           20081028
Time               10.03
INSTRUM            spect
PROBHD   5 mm PABBO BB-
PULPROG       noesypr1d
TD                 32768
SOLVENT              D2O
NS                    55
DS                     4
SWH            6410.256 Hz
FIDRES         0.195625 Hz
AQ            2.5559540 sec
RG                   812
DW                78.000 use
DE                  6.00 use
TE                 293.1 K
D1            1.00000000 sec
D8            0.05000000 sec
D11           0.03000000 sec
D12           0.00002000 sec
TD0                    1

======== CHANNEL f1 =======
NUC1                  1H
P1                 14.10 use
PL1                 0.00 dB
PL9                51.00 dB
PL1W          8.41481972 W
PL9W          0.00006684 W
SFO1         400.2318811 MHz
SI                 65536
SF           400.2299980 MHz
WDW                   EM
SSB                    0
LB                  0.30 Hz
GB                     0
PC                  1.00
```

mw465
biosyn1Hfast MeOD /opt/DATA nmrafd 2

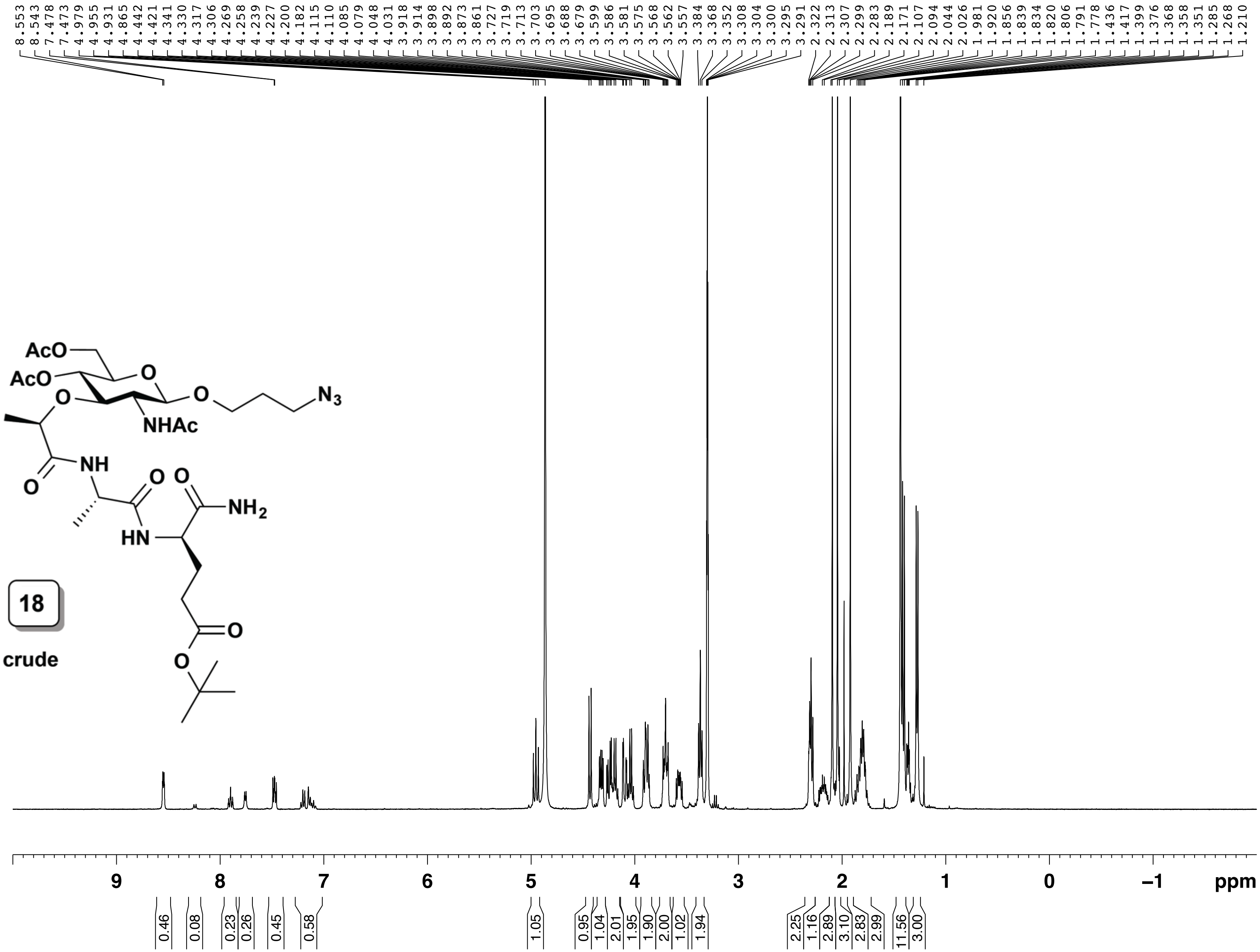

18

crude

NAME	1108Marian
EXPNO	121
PROCNO	1
Date_	20110829
Time	18.55
INSTRUM	spect
PROBHD	5 mm PABBO BB-
PULPROG	zg30
TD	65536
SOLVENT	MeOD
NS	16
DS	1
SWH	8223.685 Hz
FIDRES	0.125483 Hz
AQ	3.9846387 sec
RG	203
DW	60.800 use
DE	6.50 use
TE	296.0 K
D1	1.00000000 sec
TD0	1

======== CHANNEL f1 =======

NUC1	1H
P1	14.40 use
PL1	0.00 dB
PL1W	8.41481972 W
SFO1	400.2324716 MHz
SI	65536
SF	400.2300142 MHz
WDW	EM
SSB	0
LB	0.30 Hz
GB	0
PC	1.00

mw465
biosynAPTfast MeOD /opt/DATA nmrafd 2

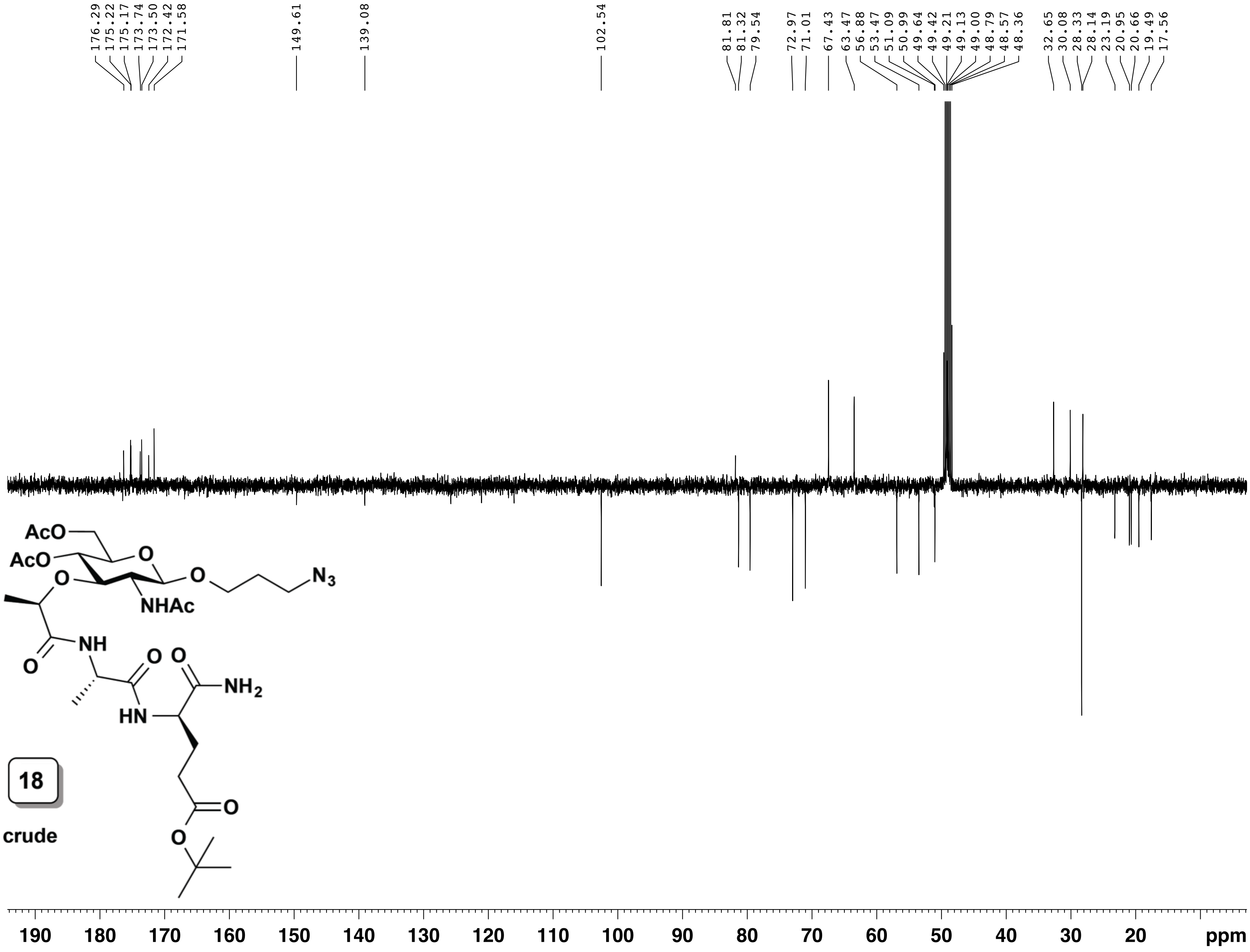

18

crude

NAME	1108Marian
EXPNO	122
PROCNO	1
Date_	20110829
Time	19.22
INSTRUM	spect
PROBHD	5 mm PABBO BB-
PULPROG	jmod
TD	65536
SOLVENT	MeOD
NS	512
DS	1
SWH	24038.461 H:
FIDRES	0.366798 H:
AQ	1.3631988 se
RG	2050
DW	20.800 us
DE	6.00 us
TE	296.8 K
CNST2	155.0000000
CNST11	1.0000000
D1	1.50000000 se
D20	0.00645161 se
TD0	1
======== CHANNEL f1 =====	
NUC1	13C
P1	9.10 us
P2	18.20 us
PL1	-1.00 dE
PL1W	44.27188873 W
SFO1	100.6479773 MI
======== CHANNEL f2 =====	
CPDPRG2	waltz16
NUC2	1H
PCPD2	80.00 us
PL2	0.00 dE
PL12	14.89 dE
PL2W	8.41481972 W
PL12W	0.27292591 W
SFO2	400.2316009 MI
SI	32768
SF	100.6377734 MI
WDW	EM
SSB	0
LB	1.00 H:
GB	0
PC	1.40

mw332
biosyn1Hfast MeOD /opt/DATA nmrafd 1

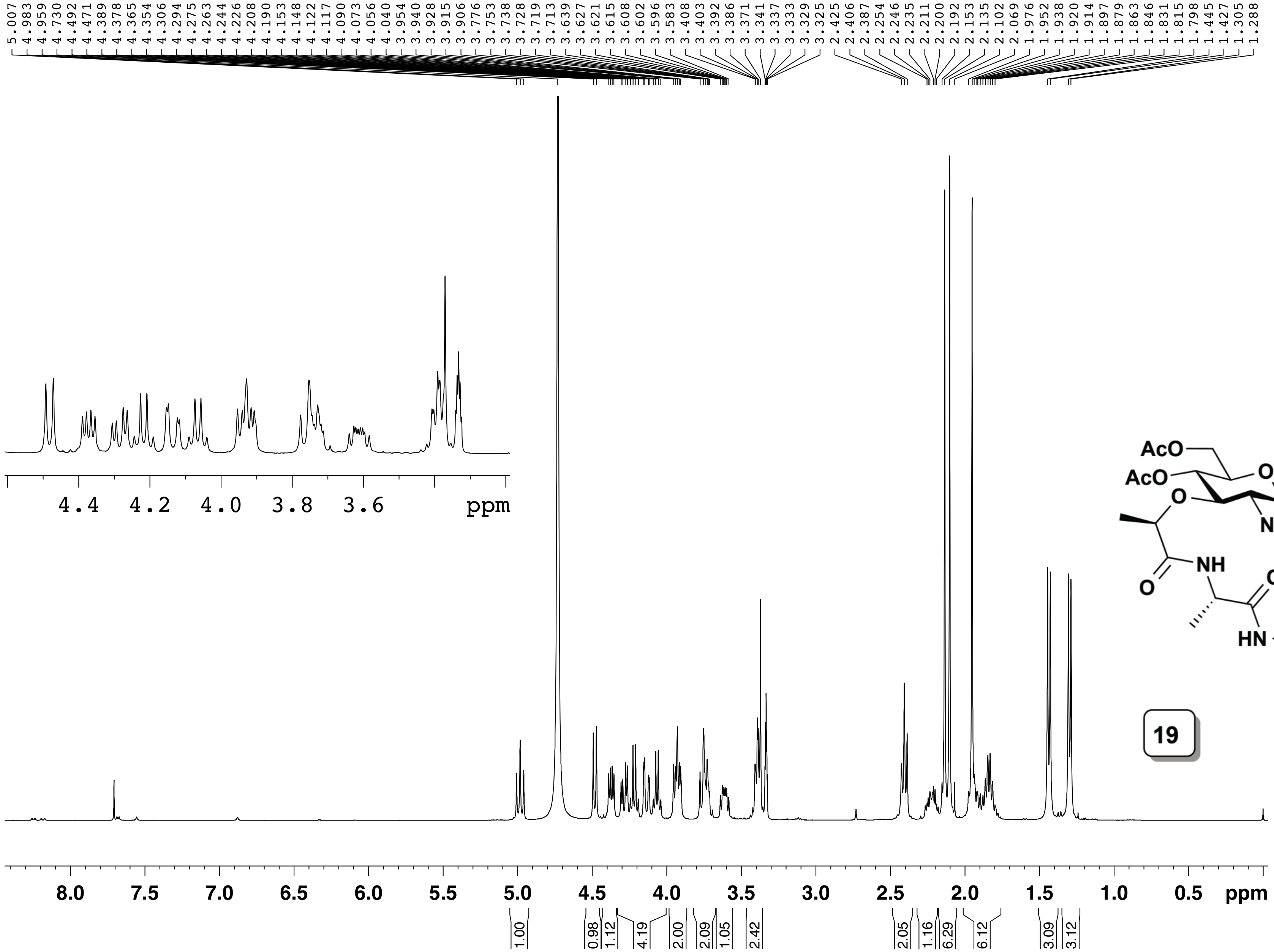

NAME	1010Marian
EXPNO	23
PROCNO	1
Date_	20101015
Time	17.22
INSTRUM	spect
PROBHD	5 mm PABBO BB-
PULPROG	zg30
TD	65536
SOLVENT	MeOD
NS	64
DS	1
SWH	8223.685 Hz
FIDRES	0.125483 Hz
AQ	3.9846387 sec
RG	101
DW	60.800 use
DE	6.50 use
TE	295.4 K
D1	1.00000000 sec
TD0	1

======== CHANNEL f1 =======

NUC1	1H
P1	14.10 use
PL1	0.00 dB
PL1W	8.41481972 W
SFO1	400.2324716 MHz
SI	65536
SF	400.2300008 MHz
WDW	EM
SSB	0
LB	0.30 Hz
GB	0
PC	1.00

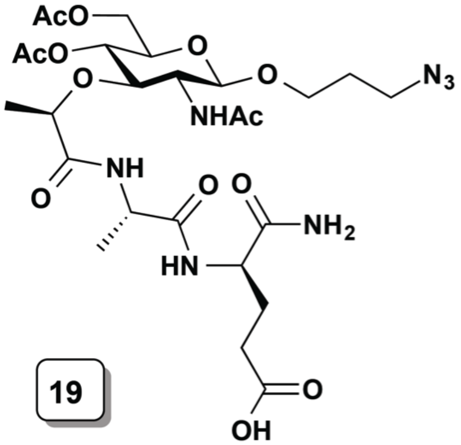

19

mw332
biosynAPTfast MeOD /opt/DATA nmrafd 1

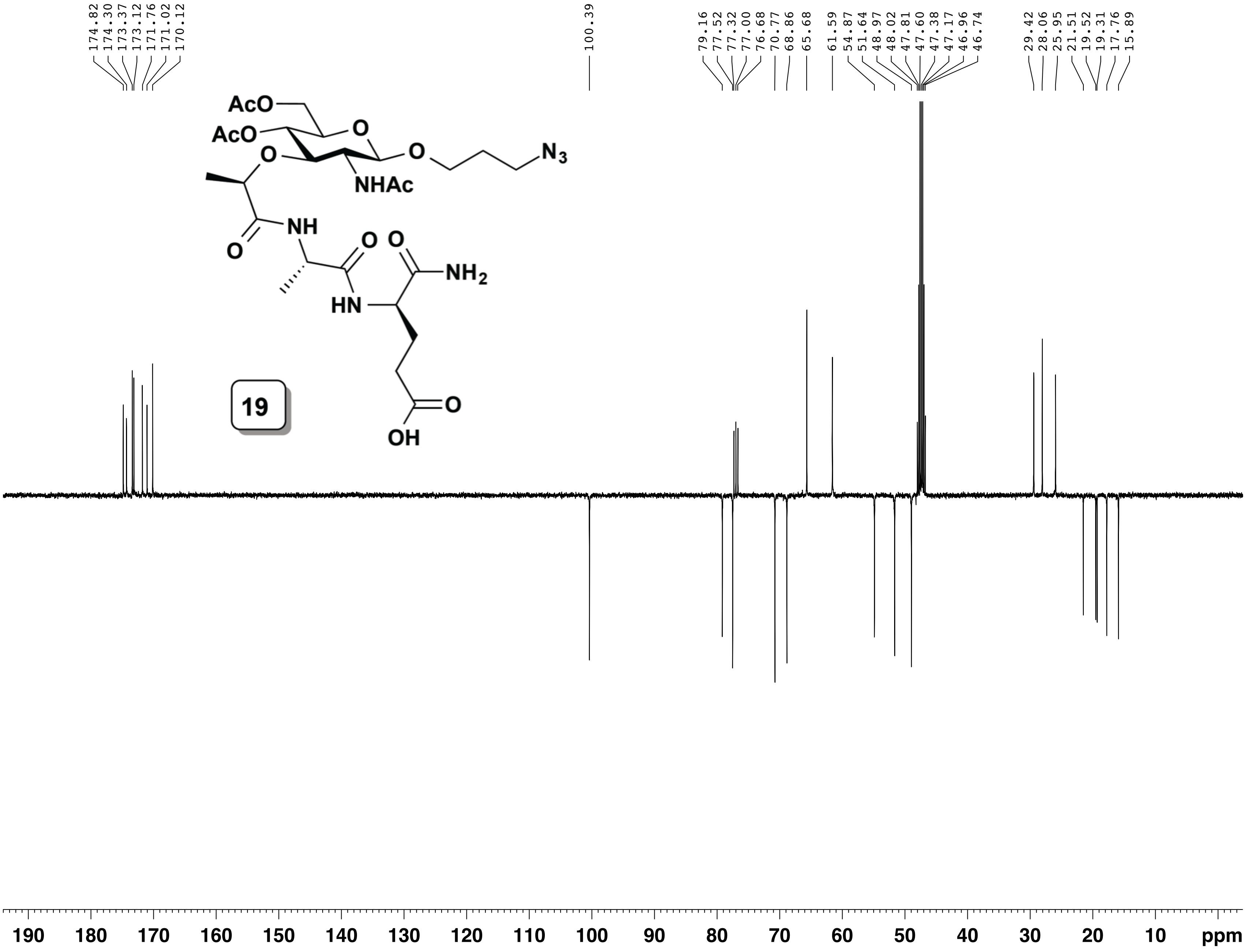

NAME	1010Marian
EXPNO	24
PROCNO	1
Date_	20101015
Time	19.53
INSTRUM	spect
PROBHD	5 mm PABBO BB-
PULPROG	jmod
TD	65536
SOLVENT	MeOD
NS	3072
DS	1
SWH	24038.461 H
FIDRES	0.366798 H
AQ	1.3631988 s
RG	2050
DW	20.800 u
DE	6.00 u
TE	296.7 K
CNST2	155.0000000
CNST11	1.0000000
D1	1.50000000 s
D20	0.00645161 s
TD0	1
======== CHANNEL f1 =====	
NUC1	13C
P1	9.10 u
P2	18.20 u
PL1	-1.00 d
PL1W	44.27188873 W
SFO1	100.6479773 M
======== CHANNEL f2 =====	
CPDPRG2	waltz16
NUC2	1H
PCPD2	80.00 u
PL2	0.00 d
PL12	15.00 d
PL2W	8.41481972 W
PL12W	0.26609996 W
SFO2	400.2316009 M
SI	32768
SF	100.6379823 M
WDW	EM
SSB	0
LB	1.00 H
GB	0
PC	1.40

1h NMR av400liq mw278 gezuiverd HB40

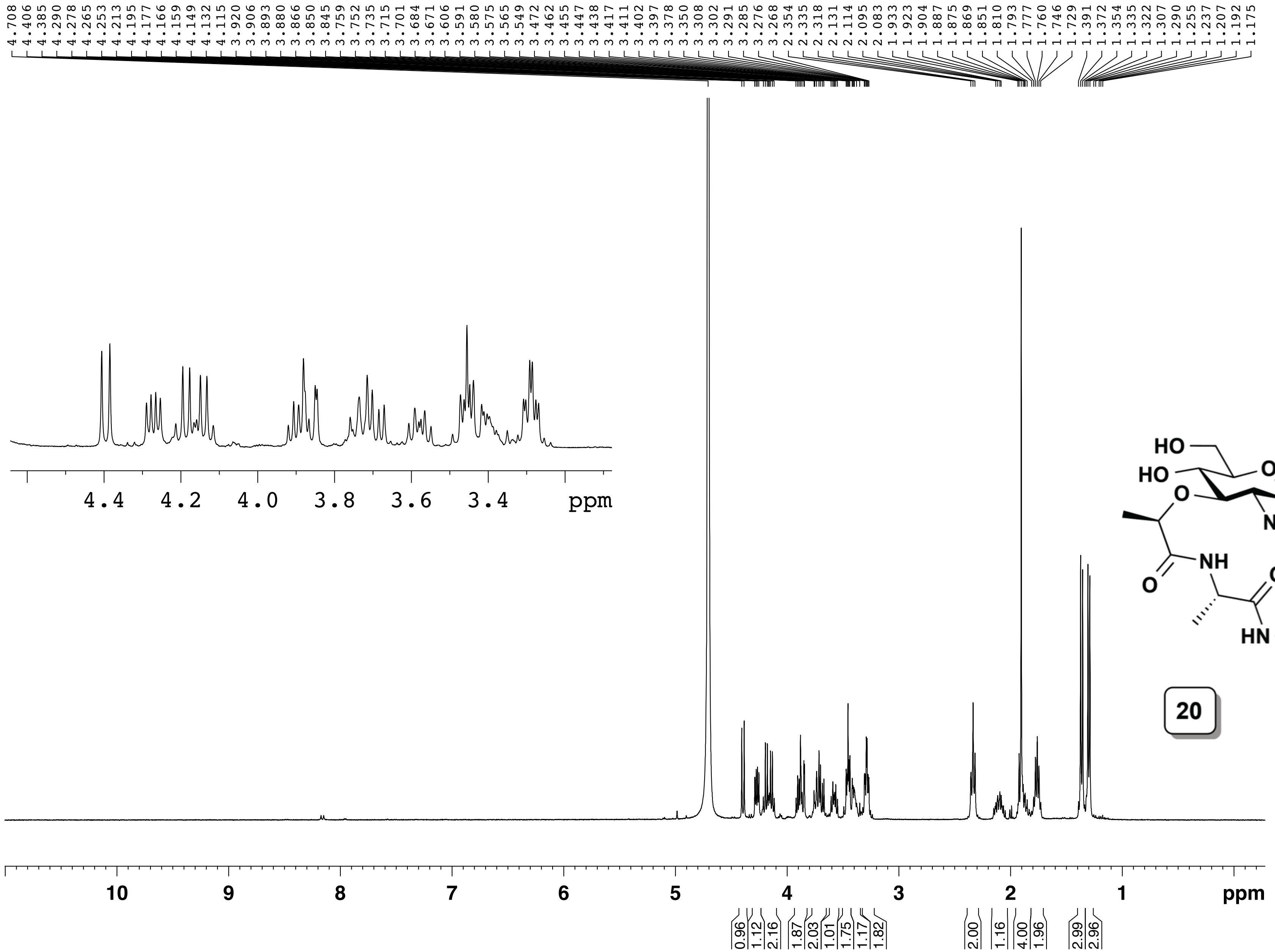

NAME	1006Marian
EXPNO	73
PROCNO	1
Date_	20100622
Time	16.34
INSTRUM	spect
PROBHD	5 mm PABBO BB-
PULPROG	zg30
TD	65536
SOLVENT	D2O
NS	152
DS	0
SWH	8223.685 Hz
FIDRES	0.125483 Hz
AQ	3.9846387 sec
RG	256
DW	60.800 use
DE	6.50 use
TE	297.6 K
D1	1.00000000 sec
TD0	1

======== CHANNEL f1 =======

NUC1	1H
P1	15.00 use
PL1	-3.00 dB
PL1W	16.78977203 W
SFO1	400.2324716 MHz
SI	65536
SF	400.2300000 MHz
WDW	EM
SSB	0
LB	0.30 Hz
GB	0
PC	1.00

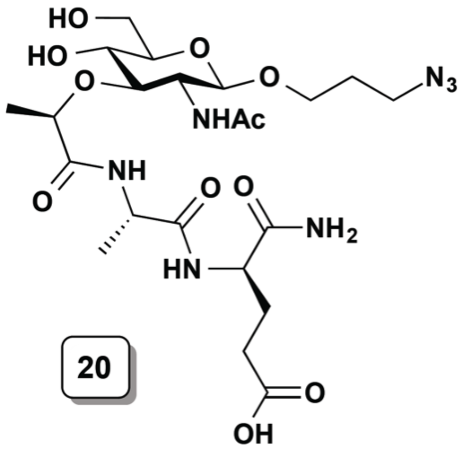

20

mw289
biosynAPTfast MeOD /opt/DATA nmrafd 5

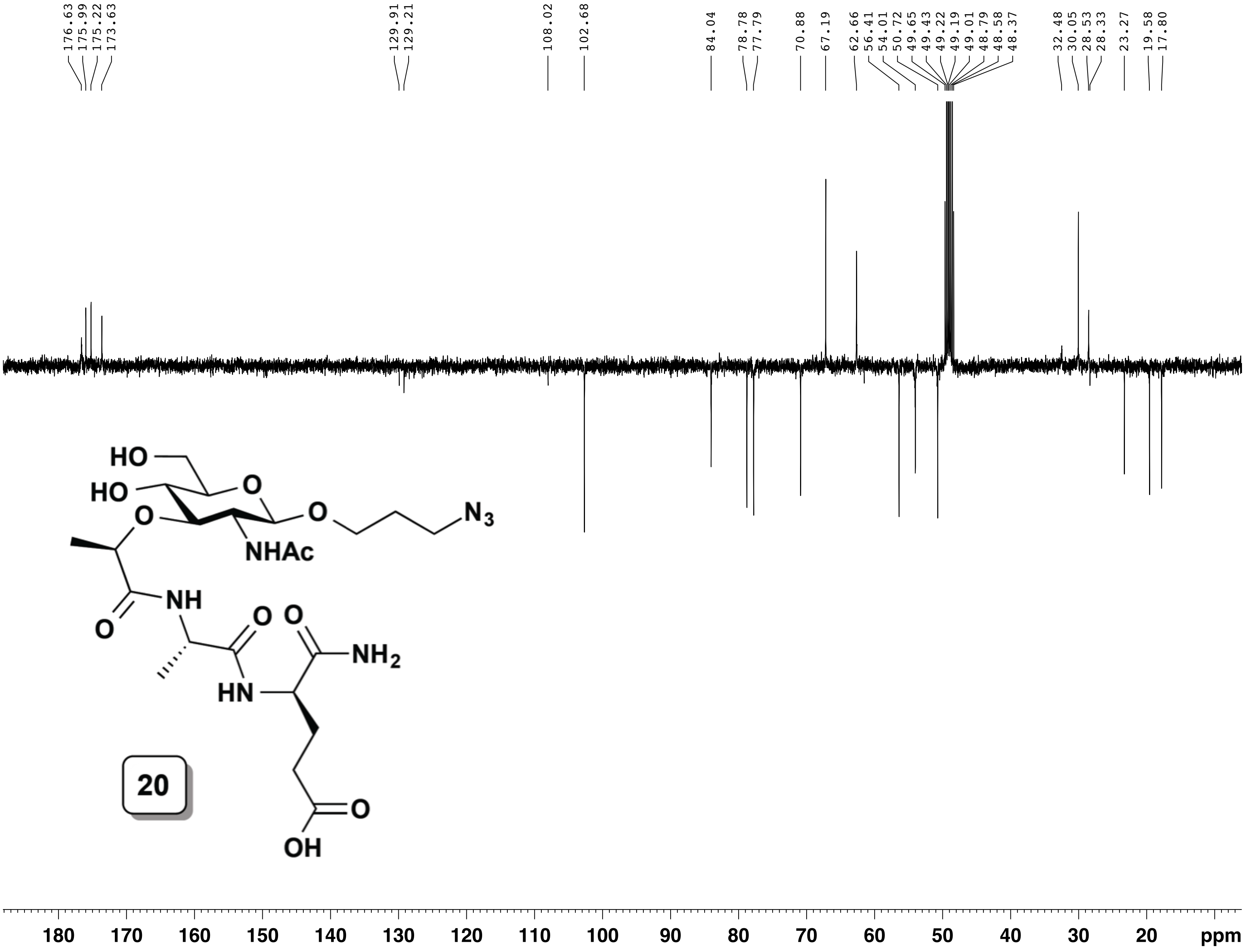

20

```
NAME           1008Marian
EXPNO                  17
PROCNO                  1
Date_            20100806
Time                23.03
INSTRUM             spect
PROBHD   5 mm PABBO BB-
PULPROG              jmod
TD                  65536
SOLVENT              MeOD
NS                    512
DS                      1
SWH             24038.461 H
FIDRES           0.366798 H
AQ              1.3631988 s
RG                   2050
DW                 20.800 u
DE                   6.00 u
TE                  297.8 K
CNST2         155.0000000
CNST11          1.0000000
D1             1.50000000 s
D20            0.00645161 s
TD0                     1

======== CHANNEL f1 =====
NUC1                  13C
P1                   9.10 u
P2                  18.20 u
PL1                 -1.00 d
PL1W          44.27188873 W
SFO1          100.6479773 M

======== CHANNEL f2 =====
CPDPRG2           waltz16
NUC2                   1H
PCPD2               80.00 u
PL2                  0.00 d
PL12                15.00 d
PL2W           8.41481972 W
PL12W          0.26609996 W
SFO2          400.2316009 M
SI                  32768
SF            100.6377751 M
WDW                    EM
SSB                     0
LB                   1.00 H
GB                      0
PC                   1.40
```
